# AML consolidation therapy: timing matters

**DOI:** 10.1007/s00432-023-05115-0

**Published:** 2023-08-03

**Authors:** Adrian-Manuel Reimann, Enrico Schalk, Felix Jost, Dimitrios Mougiakakos, Daniela Weber, Hartmut Döhner, Christian Récher, Pierre-Yves Dumas, Marc Ditzhaus, Thomas Fischer, Sebastian Sager

**Affiliations:** 1 Department of Mathematics, Otto von Guericke University (OVGU), Magdeburg, Germany; 2 Clinics of Hematology and Oncology, Otto von Guericke University (OVGU), Magdeburg, Germany; 3 Department of Internal Medicine III, University Hospital, Ulm, Germany; 4 Service d’Hématologie Clinique et de Thérapie Cellulaire, Centre Hospitalier Universitaire de Bordeaux, Bordeaux, France; 5 Service d’Hématologie, Institut Universitaire du Cancer de Toulouse Oncopole, Centre Hospitalier Universitaire, Toulouse, France; 6 R&D, Sanofi-Aventis Deutschland GmbH, Frankfurt, Germany

**Keywords:** Acute myeloid leukemia, Chemotherapy, Neutropenia, Myelosuppression, Digital twin, Mathematical modeling

## Abstract

**Purpose:**

Infections due to severe neutropenia are the most common therapy-associated causes of mortality in patients with acute myeloid leukemia (AML). New strategies to lessen the severity and duration of neutropenia are needed.

**Methods:**

Cytarabine is commonly used for AML consolidation therapy; we compared high- and intermediate-dose cytarabine administration on days 1, 2, and 3 (AC-123) versus days 1, 3, and 5 (AC-135) in consolidation therapy of AML. Recently, clinical trials demonstrated that high-dose AC-123 resulted in a shortened white blood cell (WBC) recovery time compared with high-dose AC-135. Our main hypothesis is that this is also the case for different cytarabine dosage, granulocyte colony-stimulating factor (G-CSF) administration, and cycle lengths. We analyzed 334 treatment schedules on virtual cohorts of digital twins.

**Results:**

Comparison of 32,565 simulated consolidation cycles resulted in a reduction in the WBC recovery time for AC-123 in 99.6% of the considered cycles (median reduction 3.5 days) without an increase in the number of leukemic blasts (lower value in 94.2% of all cycles), compared to AC-135.

**Conclusion:**

Our numerical study supports the use of AC-123 plus G-CSF as standard conventional AML consolidation therapy to reduce the risk for life-threatening infectious complications.

**Supplementary Information:**

The online version contains supplementary material available at 10.1007/s00432-023-05115-0.

## Introduction

Patients with acute myeloid leukemia (AML) are at high risk of infectious complications due to the nature of the disease and its treatment (Crawford et al. 2004; Nesher and Rolston 2014). Neutropenia, a major risk factor for infections in patients with AML, is characterized by an absolute neutrophil count (ANC) $<500/\upmu {\text {L}}$ that results in increased severity and frequency of infections (Bodey et al. 1966). Cytarabine (Ara-C) is a well-established chemotherapeutic agent used for remission induction and post-remission (i.e., consolidation) treatment of patients with AML (Döhner et al. 2017). However, the optimal Ara-C dosing and timing during consolidation are unclear (Döhner et al. 2017; Jaramillo and Schlenk 2021). We compared the following two common Ara-C therapy schedules: administration on days 1, 2, and 3 (AC-123); and on days 1, 3, and 5 (AC-135). High-dose ($3\frac{g}{m^2}$) and intermediate-dose (1–$1.5\frac{g}{m^2}$) Ara-C are often administered as 3-h intravenous infusions, referred to as HDAC-123 and IDAC-123 or as HDAC-135 and IDAC-135, based on the treatment schedule. The effect of HDAC-123 was compared to that of HDAC-135 in consolidation therapy for young adult patients with AML in a previous clinical trial (Jaramillo et al. 2017). The clinical trial is also considered the possible administration of granulocyte colony-stimulating factor (G-CSF) for accelerating white blood cell (WBC) reconstitution. Patients treated with HDAC-123 ($n=392$) had a shorter hematologic recovery time (by a median of 4 days) with WBC $>1000/\upmu {\text {L}}$ and neutrophils $>500/\upmu {\text {L}}$ than those treated with HDAC-135 ($n=176$) ($p<0.001$), which was further reduced by 2 days ($p<0.001$) by pegylated G-CSF (i.e., pegfilgrastim) administered on day 8. However, no significant difference ($p=0.90$) was observed between HDAC-135 ($n=135$) and HDAC-123 ($n=392$) in terms of overall survival (OS) (Jaramillo et al. 2017), which was consistent in another clinical trial with 221 patients (Dumas et al. 2020) who were administered pegfilgrastim on day 5 (HDAC-123) and 7 (HDAC-135). The hematological recovery times for neutrophils were significantly shorter in patients receiving HDAC-123, with an average difference of 3–4 days for each consolidation cycle (CC) ($p<0.001$ for each cycle). Although these clinical trials reported no significant differences in OS between HDAC-123 and HDAC-135 (Hazard ratio [HR] 1.40 [95% confidence interval 95% CI 0.85–2.31]; $p=0.19$), the reported differences in hematological recovery warrant further investigation.

Further analysis of clinically relevant outcomes is thus needed to establish optimal clinical standards for post-remission AML therapy. Is IDAC-123 superior as compared to IDAC-135? What is the impact of different treatment strategies administering G-CSF compared to those opted for in the above studies? What is the impact of CC lengths? Considering the large number of possible combinations for such treatment decisions, conducting clinical studies for all the above-mentioned gaps in knowledge is not feasible. Utilizing virtual cohorts of digital twins is a promising approach for overcoming the combinatorial complexity (Sager 2023). It also enables studying the dynamics of absolute leukemic blast numbers, which is very challenging from an observational point of view in the case of remission, when by definition the bone marrow contains fewer than 5% blast cells.

Our main hypothesis was that AC-123 is better than AC-135 for WBC recovery time, which we used as a key performance indicator for neutropenia/leukopenia (kpiL), and not significantly inferior considering the dynamics of leukemic blasts (kpiB) under a large variety of treatment choices.

## Materials and methods

**Fig. 1:**
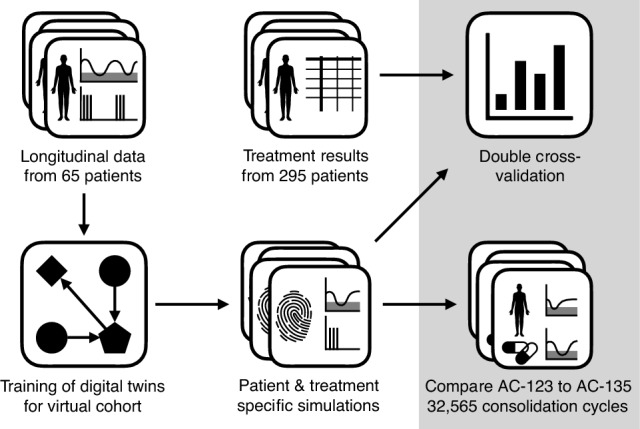
Our workflow built on retrospective longitudinal data from $n=65$ patients. Most importantly, the data contained 1869 white blood cell (WBC) measurements as well as cytarabine (Ara-C) and lenograstim timing and dosage. We used these data together with a pre-trained mathematical model published in the previous studies (Jost et al. 2019, 2020) to estimate six patient-specific model parameters. This allowed patient- and treatment-specific simulations in a virtual cohort. In addition to a first cross-validation reported in Jost et al. (2019), here, we compared the distributions of individual WBC recovery times to those of two independent clinical studies (Dumas et al. 2020; Jaramillo et al. 2017). By satisfying with the close similarity of the simulated predictions to clinical data, we compared the effect of Ara-C administration on days 1, 2, and 3 (AC-123) versus days 1, 3, and 5 (AC-135) for 334 simulated treatment schedules with varying administration of lenograstim, Ara-C dosage, and time between two consecutive cycles. As key performance indicators for a treatment, we evaluated key performance indicators for neutropenia/leukopenia (kpiL) and differences in simulated leukemic blast ratios (kpiB). The main result was a superiority of AC-123 compared to AC-135 in 99.6% of the considered cycles, using the same virtual cohort

Our approach is illustrated in Fig. 1.

**Fig. 2:**
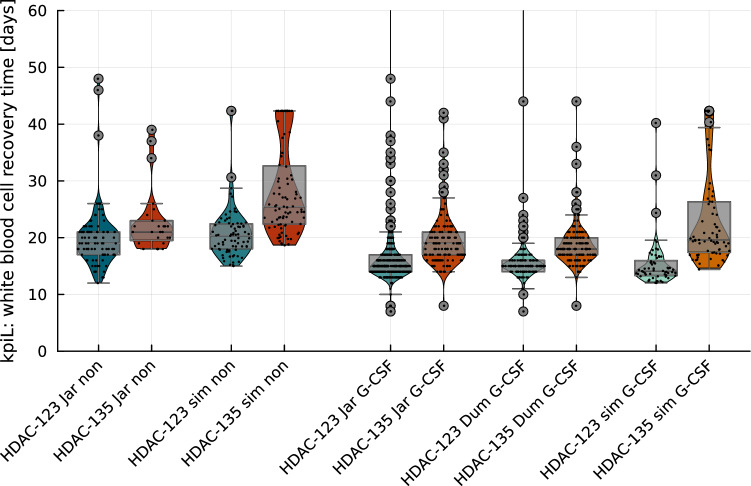
We compared WBC recovery time as a key performance indicator (kpiL) for different treatments. For different treatment schedules on the horizontal axis, dots indicate kpiL values from one of the consolidation cycles of different patients. The distributions, including median, standard deviations, and outliers, are illustrated. The treatments were always compared between HDAC-123 (123, blue) and HDAC-135 (135, red). Columns 1–2 show data from the clinical trial Jar (Jaramillo et al. 2017) for a cohort without granulocyte colony-stimulating factor (G-CSF) treatment (non); columns 3–4 show simulated data for our HDAC-virtual cohort of patients (sim) without G-CSF (non). For columns 5–10, either pegfilgrastim or lenograstim was administered (G-CSF). Columns 5–6 show the corresponding cohorts of Jar, columns 7–8 of another clinical trial Dum (Dumas et al. 2020), and columns 9–10 simulated values. The HDAC-virtual cohort used in columns 3, 4, 9, and 10 is approximately 10 years older compared to the Jar and Dumas cohorts shown in the other columns (compare Table 1), giving an explanation for slightly increased kpiL values. We visually demonstrate that the HDAC-123 treatments (in comparison to HDAC-135) resulted in a significant reduction in kpiL in all five comparisons and that the simulations resulted in very similar WBC recovery times when compared to the clinical studies

We started with retrospective, longitudinal data focusing on WBC counts. A large variety of mathematical models have been proposed for different purposes in oncology, see (Sager 2023; Chulián et al. 2022; Stiehl and Marciniak-Czochra 2012; Michor and Beal 2015; Brady and Enderling 2019; Clairambault 2009) for surveys and further references. We trained and cross-validated a previously published mathematical model (Jost et al. 2019, 2020) containing compartmental submodels for hematopoiesis (Jost et al. 2019; Friberg et al. 2002), pharmacokinetics and pharmacodynamics of Ara-C (Jost et al. 2019), leukemic blasts (Stiehl et al. 2018), and G-CSF (Jost et al. 2020). This model was developed with two main properties. First, to capture the most important physiological processes linking Ara-C and G-CSF treatment with hematological outcomes. Second, to have few model parameters that can be estimated from longitudinal WBC count data. The model is pre-trained in the sense that some parameters were fixed to values from the literature. Hence, additional data, e.g., from drug concentration measurements, were used previously to estimate these parameters in the submodels (Jost et al. 2019, 2020). By estimating the free model parameters [which were identified in Jost et al. (2019) and Friberg et al. (2002) to be a good choice in the interest of a good balance between identifiability and goodness-of-fit], we obtained personalized mathematical models that we will refer to as *digital twins* (Sager 2023) in the following. We used them to simulate the AC-123 and AC-135 schedules for 334 different treatment choices as specified below. Two of these correspond to those investigated in the previous studies (Jaramillo et al. 2017; Dumas et al. 2020), with only minor differences in G-CSF administration. We did a second cross-validation by comparing the obtained WBC recovery times with those of the clinical studies. The small standard deviations of the parameter estimation provided in the supplement showed that all model parameters were identifiable with the available longitudinal data (most importantly, 1869 WBC observations). The standard deviations of the simulated outcomes of the virtual cohorts show that 3 CCs for 65 patients are statistically significant. Based on the non-significant differences between simulated and real clinical results ($p=0.63$, see below), we postulated that the other 332 treatment simulations also provide a good indication of real-world treatment outcomes. Additionally, we see this and the visual similarity of the distributions in Fig. 2 for two specific treatments as an indication that the comparatively small virtual cohort of 65 digital twins is a good representative for the cohorts with almost 1000 patients included in the clinical trials (Jaramillo et al. 2017; Dumas et al. 2020).

### Data, model, and methods for the virtual cohorts

We trained 65 digital twins using longitudinal retrospective data. The data were obtained retrospectively from the phase II AMLSG 12-09 randomized-controlled trial (RCT; $n=44$) and from clinical chart records from the Magdeburg University Hospital, Germany ($n=21$). This study was approved by the ethics committee of the Magdeburg University Hospital, Approval No. 124/15. The group was heterogeneous considering Ara-C administration (with $n=30$ receiving HDAC-123 or HDAC-135 Ara-C of 3 g/m^2^ and n=35 receiving IDAC-123 or IDAC-135 Ara-C of 1–1.5 g/m^2^), lenograstim administration (some received $263\,\upmu {\text {g}}$, some no G-CSF at all), age (median 58.5 years), and number of recorded CCs. In clinical trials, heterogeneity may be a disadvantage. However, to train mathematical models, heterogeneity is an advantage. The mathematical model was derived and cross-validated (mainly with 1869 longitudinal WBC count data, see supplement for details) by our group (Jost et al. 2019, 2020). Using a population parameter estimation (nonlinear mixed-effects modeling) approach with the software NONMEM 7.5.0, we obtained six individual model parameters for each patient. The model, values for all the parameters, and validation results are specified in the online supplement. Hereafter, we refer to the mathematical model together with six estimated model parameters as digital twin, and the sets of 30 and 35 digital twins resulting from high-dose and intermediate-dose Ara-C data sets as HDAC-virtual cohort and IDAC-virtual cohort, respectively.

### Treatment choices

We defined a treatment as a particular choice: (i) between AC-123 and AC-135; (ii) days when $263\, \upmu {\text {g}}$ of lenograstim was administered; and (iii) the length of the three considered CCs. To obtain better prediction quality, we fixed the drug doses to those of the training data, that is, either 3 g/m^2^ Ara-C for the HDAC-virtual cohort (HDAC-123 or HDAC-135) or 1 g/m^2^ for the ID-virtual cohort (IDAC-123 or IDAC-135) and $263\, \upmu {\text {g}}$ daily dose of lenograstim. The timing of Ara-C was fixed to either days 1, 2, and 3 (HDAC-123 or IDAC-123) or to days 1, 3, and 5 (HDAC-135 or IDAC-135). Altogether, for both AC-123 and AC-135, we evaluated $n=334$ different treatment schedules ($111\times 2+(29-1)\times 2\times 2=334$). First, we defined 111 different lenograstim schedules while considering the cycle length as fixed to 42 days. The schedules varied in the starting days with respect to the last day of Ara-C and in duration (number of administration days), evaluated on the grids from 1 to 10 and from 1 to 21 in steps of 2 days, respectively. For example, a starting day of 3 corresponded to lenograstim treatment starting on day 6 for AC-123, and day 8 for AC-135. Thereafter, we fixed two lenograstim schedules (start day 5 with 5 day duration and no G-CSF) and considered 29 different lengths (from 28 to 56 days, with 42 already included above) for all three CCs. All the treatments pertained to the HDAC-virtual and IDAC-virtual cohorts. Thus, the setup could be considered as a huge study comprising 334 schedules, all comparing the AC-123 and AC-135 schedules.

### Predicting treatment outcomes

The outcome of treatments was determined via simulation (the numerical solution of differential equations) with patient-specific model parameters. We employed the RosenbrockW6S4OS method of the julia package DifferentialEquations.jl. For all the digital twins and all treatments, we simulated three consecutive CCs. Postprocessing the simulation results, we extracted two key performance indicators for each digital twin and each treatment: the WBC recovery time as an indicator of leukopenia severity (kpiL) and the ratio between the absolute leukemic blast number at the end of a CC divided by the number at the beginning of consolidation therapy (kpiB). As discussed in the online supplement, there are alternative key performance indicators to evaluate treatments. We used WBC recovery time as an indicator for leukopenia (kpiL), because it is a clinically established outcome. We used the leukemic blast ratio as kpiB because of several advantages: it is readily interpretable (a value $<1.0$ indicates a reduction of leukemic blasts compared to the beginning of treatment), it is sensitive against treatment choices, and it is almost in-sensitive against the unknown number of leukemic blasts at the beginning of treatment (see Online Supplement 6 for an illustration and discussion). Note that being able to evaluate kpiB is a unique advantage of digital twins compared to clinical practice.

### Data and methods for cross-validation

We obtained tabulated data containing information on the kpiL values as well as corresponding treatment and age from two clinical studies that compared HDAC-123 and HDAC-135 (Jaramillo et al. 2017; Dumas et al. 2020). To perform a second cross-validation of the digital twins, we compared the kpiL values of the different treatment arms of these studies to those of our HDAC-virtual cohort. For comparability, we only considered patients who received identical treatments for three consecutive cycles with WBC $<1 000/\upmu {\text {L}}$ in all the cycles. The details of the resulting cohorts are provided in Table 1.

**Table 1:** Numbers of consolidation cycles and age of patients included in cohorts of Fig. 2

HDAC-	No G-CSF (non)				G-CSF					
	Jar		Sim		Jar		Dum		Sim	
	123	135	123	135	123	135	123	135	123	135
*n*	90	27	78	87	357	135	129	147	60	72
Median age (years)	50.5	49	57.5	58	46	47	47	41	56	57

Number of considered consolidation cycles *n* and median age of the corresponding patients for the ten cohorts depicted in Fig. 2. The ten cohorts were ordered according to whether pegfilgrastim or lenograstim was administered in all consolidation cycles (G-CSF) or not (non), according to the origin of the data with Jar (Jaramillo et al. 2017), Dum (Dumas et al. 2020), and sim (HDAC-virtual cohort of digital twins), and according to the days on which high-dose cytarabine (HDAC) was administered (either days 1, 2, and 3 or days 1, 3, and 5). For comparability, we only considered patients who received identical treatments for three consecutive cycles with WBC counts $<10^9 {\text {/L}}$ in all cycles. This resulted in different numbers of cycles, although the same virtual cohort was used in columns 3, 4, 9, and 10 ($n=30$, median age $=58.5$). For the IDAC-virtual cohort of $n=35$ patients, a different Ara-C dosage was administered and simulated (denoted as IDAC-123 and IDAC-135), and these data are not shown here as the dose for all cycles in Jar and Dum was fixed to $3 \, {\text {g}}/{\text {m}}^2$

The clinical trial by Jaramillo et al. (2017) included one cohort of patients who received no G-CSF and one cohort who received 6 mg pegfilgrastim on day 8 (HDAC-123) and day 10 (HDAC-135) of each cycle. The data set by Dumas et al. (2020) contains only one cohort with data for HDAC-123 and HDAC-135 after exclusion of non-consistent cycle data as described above. This cohort received 6 mg pegfilgrastim on days 5 (HDAC-123) and 7 (HDAC-135) of each cycle. Since our mathematical model was trained with data corresponding to lenograstim treatments, we needed to identify a lenograstim schedule corresponding to these pegfilgrastim treatments. We are not aware of any detailed pharmacokinetic comparisons between pegfilgrastim and lenograstim. We opted for a “start day 5 and 5 days duration” schedule, because the start date (day 8 for HDAC-123 and day 10 for HDAC-135) was identical to that in the Jar clinical trial and a 5-day-administration seemed to be a plausible clinical alternative (for example, Granocyte^®^ contains five units).

Although the cross-validation of the mathematical model in Jost et al. (2019) and in the online supplement demonstrated very good accuracy, an intrinsic shortcoming of machine learning is that the accuracy of predictions may deteriorate when extrapolating beyond the training data. This happens when treatments that differ from those in the training data are applied to digital twins. An unavoidable problem, because a patient can receive only one treatment at a time. Thus, we propose a double cross-validation as a partial remedy. This novel model development and validation workflow with high scientific rigor could strengthen the trust in the validity of mathematical models. In addition to the first cross-validation based on splitting WBC values into training and test sets (Jost et al. 2019) as discussed in the online supplement, we extracted the kpiL values and compared them with the values reported in the literature. Statistical tests were performed for various cohorts. A multivariate analysis of the WBC recovery times (kpiL) of all the CCs was performed using an extended Cox regression model by applying the method of Wei et al. (1989). In this analysis, we considered the factors HDAC-123 versus HDAC-135, G-CSF administration, age, and simulated versus actual clinical outcomes. Further variables, such as sex, were not considered, because there was incomplete data or they were already shown to be not significant (Jaramillo et al. 2017).

## Results

### Double cross-validation

Figure 2 shows a visualization of the distributions of WBC recovery time for the clinical cohorts (Jaramillo et al. 2017 (Jar); Dumas et al. 2020) and for the virtual cohorts generated by our model.

Of note, HDAC-123 treatment resulted in a significant reduction in kpiL (the median was reduced by approximately 4 days) in all the five comparisons. In the Jaramillo data (columns 1, 2, 5, and 6 of Fig. 2) and in the simulations (columns 3, 4, 9, and 10), G-CSF administration resulted in an additional reduction of kpiL median values and more compact distributions. The distributions of WBC recovery times are similar for clinical and for virtual cohorts that received the same treatments. Table 2 presents a statistical confirmation of this visual impression. While the simulations confirmed the advantage of HDAC-123 over HDAC-135, the WBC recovery times of the HDAC-virtual cohort were not significantly different from the WBC recovery times obtained from the clinical studies included in this analysis.

**Table 2:** Hazard ratios for reduced white blood cell (WBC) recovery time upon administration of HDAC-123 compared to HDAC-135 in clinical and virtual studies

	Hazard ratio	95% CI	*p* value
Data: (Jaramillo et al. 2017) (Jar), columns 1, 2, 5, 6			
HDAC-123	2.10	1.66–2.64	$\le 0.0001$
G-CSF	2.21	1.70–2.87	$\le 0.0001$
Age	0.80	0.71–0.89	$\le 0.0001$
Data: (Dumas et al. 2020) (Dum), columns 7, 8			
HDAC-123	2.49	1.81–3.43	$\le 0.0001$
Age	1.10	0.97–1.25	0.127
Data: sim and Jar, columns 1–6, 9–10			
HDAC-123	2.52	2.00–3.18	$\le 0.0001$
G-CSF	2.28	1.77–2.94	$\le 0.0001$
Age	0.85	0.77–0.94	0.002
Simulated	0.93	0.69–1.25	0.627
Data: all, columns 1–10			
HDAC-123	2.42	2.02–2.90	$\le 0.0001$
G-CSF	2.33	1.83–2.96	$\le 0.0001$
Age	0.91	0.84–0.98	0.017
Simulated	0.89	0.67–1.17	0.407

Wei–Lin–Weissfeld model for WBC recovery times. Data, columns, and abbreviations Jar (Jaramillo et al. 2017), Dum (Dumas et al. 2020), and sim (Simulated) correspond to Fig. 2 and Table 1. HDAC-123 was compared to HDAC-135, administration of either pegfilgrastim or lenograstim (G-CSF) was compared to no G-CSF, and “Simulated” indicated data of columns 3, 4, 9, and 10 were compared against all other columns of Table 1. The hazard ratio for the impact of age was scaled to an age difference of 10 years. Results for the first two studies (Jar and Dum data) were published previously and are shown here for convenience. The inclusion of the HDAC-virtual cohorts substantiated the significant influence of HDAC-123 and G-CSF treatments, while the simulated values were not significantly different from the clinical ones. This was even evident when Dum data were included, where pegfilgrastim was administered on different days of the consolidation cycle

### A study on a virtual cohort of digital twins

**Fig. 3:**
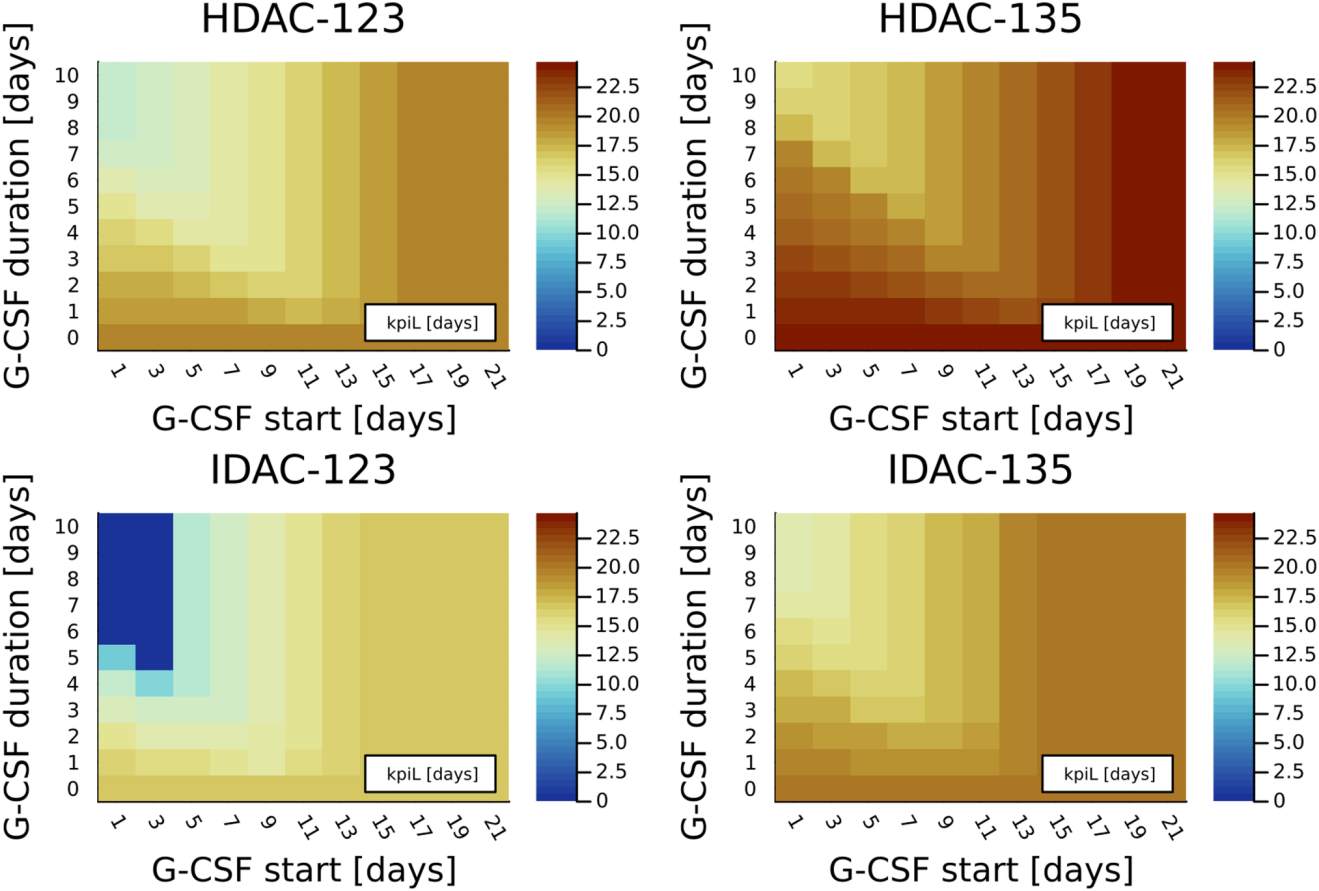
The heatplots show median values of the WBC recovery times (key performance indicator, kpiL) for different simulated lenograstim schedules and the first consolidation cycle per patient. The four subplots show different chemotherapy schedules, namely HDAC-123, HDAC-135, IDAC-123, and IDAC-135. HDAC indicates high Ara-C dosage of $3 \frac{g}{m^2}$ of body surface area, IDAC intermediate dosage of $1 \frac{g}{m^2}$. The numbers indicate the days of drug administration (either day 1, 2, and 3 or day 1, 3, and 5). The median is either taken from the HDAC-virtual cohort with $n=30$ patients (for HDAC-123 and HDAC-135) or from the IDAC-virtual cohort ($n=35$, for IDAC-123 and IDAC-135). Within the subplots, the lenograstim timing is varied with fixed daily dosage of $263\, \upmu {\text {g}}$. On the horizontal axis, we compared different starting points, where the number indicates the number of days between the end of the Ara-C treatment and the start of the Lenograstim treatment, while the vertical axis decodes the number of consecutive days lenograstim was administered. For example, G-CSF start 5 and G-CSF duration 5 correspond to 5 consecutive days of lenograstim treatment starting at day 8 for AC-123, and day 10 for AC-135. No granulocyte colony-stimulating factor (G-CSF) (non) corresponds to the field at duration 0. In all the subplots, we observed a valley corresponding to lower kpiL values that starts around G-CSF start 13 and G-CSF duration 1 and reaches the minimum in the top left corner. For later G-CSF starts, no impact on the first consolidation cycle was observed any more. Decreased kpiL values were observed for HDAC-123 and IDAC-123 in comparison to HDAC-135 and IDAC-135, respectively, for all of the lenograstim schedules, including no lenograstim application

To comprehensively study the differential outcome of AC-123 versus AC-135 administration with and without application of G-CSF, an extended study on WBC recovery times was performed. To account for a large variety of possible G-CSF administrations, we evaluated 111 different possibilities in start and duration of administering G-CSF ($263 \, \upmu {\text {g}}/{\text {day}}$). The CC length was fixed at 42 days. Figure 3 shows 444 median kpiL values resulting from the various lenograstim schedules and from HDAC-123, HDAC-135, IDAC-123, and IDAC-135 schedules, respectively.

Looking at the four heatplots individually, one observes the important impact of the G-CSF schedules on kpiL. The numerical study supports the current clinical procedure of starting approximately a week after the end of chemotherapy (horizontal axis) and administering for several days (vertical axis). Comparing the heatplots to the left with those to the right, one observes the clear advantage of HDAC-123 compared to HDAC-135 and of IDAC-123 compared to IDAC-135 for all investigated schedules of G-CSF. In the same setup, we compared different key performance indicators, which can be done in a straightforward way by extracting information from the solutions of the system of differential equations. In the online supplement heatplots for the WBC nadir values and for leukopenia duration are shown, giving qualitatively similar results as Fig. 3.

**Fig. 4:**
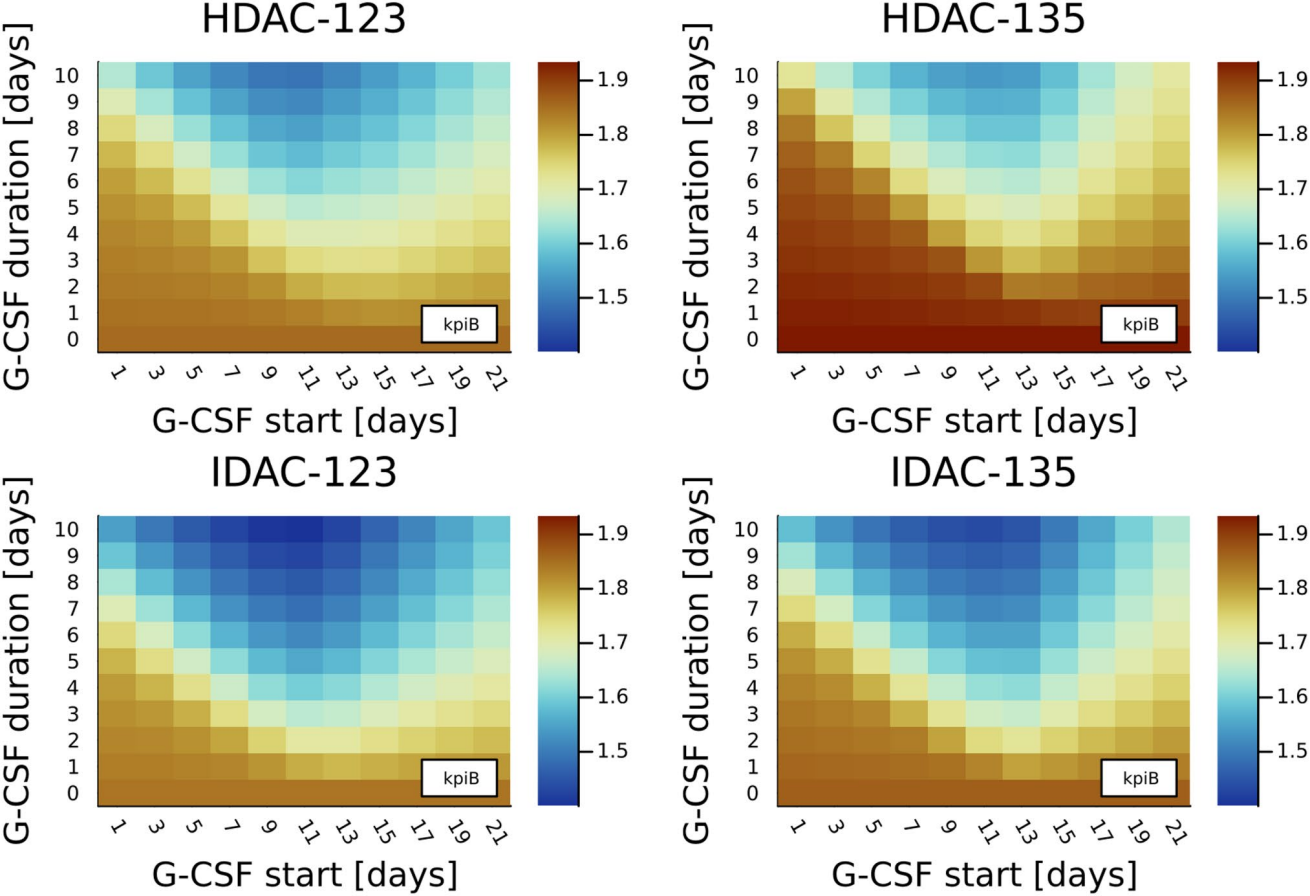
Similar to Fig. 3, but for the median values of the ratios of simulated absolute leukemic blast numbers at the end and at the beginning of the first consolidation cycle, denoted as key performance indicator kpiB. By design, a decrease in the leukemic blasts over the cycle would correspond to a kpiB value below 1, while most values for a cycle length of 42 days are above 1. Granulocyte colony-stimulating factor (G-CSF) administration reduces the ratio in all the settings, with best performance starting approximately 11 days after the end of the chemotherapy. We observe that AC-123 treatment (two subplots to the left) does not result in worse (but rather slightly better) outcomes compared to AC-135 (right) considering proliferation of leukemic blasts

In addition, we evaluated kpiB values based on the simulated dynamics of absolute leukemic blast numbers, as also discussed in more detail in the online supplement. Figure 4 shows heatplots for these values, indicating that the outcome of HDAC-123 and IDAC-123 as compared to HDAC-135 and IDAC-135, respectively, is not worse (but rather better) considering the impact of the chemotherapy schedule on the dynamics (and hence persistence) of leukemic blast numbers (kpiB).

**Fig. 5:**
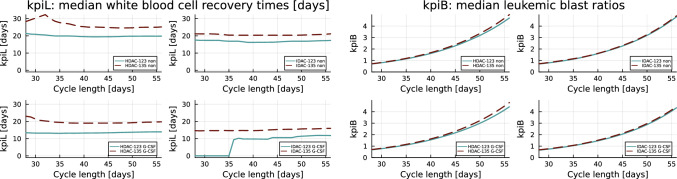
Left four subplots: similar to Fig. 3, but now the median values of white blood cell recovery times (key performance indicator, kpiL) are shown as one-dimensional plots with the consolidation cycle length in days (CC dist) as independent variable. One can observe that for high (HDAC-123) and intermediate dose (IDAC-123) of cytarabine (Ara-C) and for administration of no lenograstim (non, topmost subplots) and of lenograstim for 5 days starting at day 5 (G-CSF, subplots 3 and 4), the median kpiL values of AC-123 are better than those of AC-135, respectively. Right four subplots: the same setting, but similar to Fig. 4, the ratios between the leukemic blasts at the end and the beginning of the first consolidation cycle (kpiB) are plotted. In simulation, the cycle lengths exceeding 35–42 days resulted in greatly increased numbers of leukemic blasts. Similar to Fig. 4, AC-123 did not lead to worse outcomes compared to AC-135 considering the numbers of leukemic blasts

To evaluate the impact of the CC length on kpiL and kpiB, we considered two major clinical G-CSF settings: either no G-CSF or G-CSF started at day 5 once daily for 5 days, as used for the data depicted in Fig. 2. Like Figs. 3 and 4, Fig. 5 shows the median kpiL (left panel) and kpiB (right panel) values, here resulting from different CC lengths. We also implemented strategies that are closer to clinical practice, such as individually starting the next CC when the WBC count is above a certain threshold (data not shown). In all the considered scenarios, AC-123 resulted in a significant reduction in kpiL but no meaningful deterioration of kpiB.

Interestingly, the Wei–Lin–Weissfeld model demonstrated an HR for reduced WBC recovery time of 1.57 (95% CI 1.53–1.61, $p<0.0001$) for IDAC administration as compared to HDAC administration. Comparisons of specific treatments in Figs. 3, 4, and 5 demonstrate consistently lower median kpiL values and approximately identical kpiB values for IDAC administration in comparison to HDAC. However, these comparisons between HDAC and IDAC administration must be considered with extreme caution, as they are based on two different virtual cohorts.

In total, AC-123 resulted in 99.6% of the considered 32 565 CCs in a reduction of kpiL (median of 3.5 d) compared to AC-135. The HR was 2.17 (95% CI 2.11–2.23, $p<0.0001$). No significant increase in the absolute number of leukemic blasts was observed. In fact, a lower value was observed in 94.2% of all the CCs. We conclude that AC-123 is better in silico than AC-135 for WBC recovery time, which we used as a key performance indicator for leukopenia, and not significantly inferior considering the dynamics of leukemic blasts under a large variety of treatment choices.

## Discussion

### Clinical implications

AML consolidation therapy should be optimized to reduce patient risk of infection without endangering the main goal of post-remission maintenance. The interactions between AML treatments and the immune system are complex and influenced by several factors, including treatment dose, combination, frequency, and duration. For example, deciding when to start the next CC is challenging, since hematological recovery and the main therapy objective must be balanced. Our results shed additional light on the statement “We found that a delay $\ge 40$ days between HDAC cycles 1 and 2 was independently and significantly associated with poorer OS rate. We have no clear explanation for this intriguing result, which should be interpreted with caution and should prompt additional investigations. If confirmed, this could have important implications for routine practice.” Dumas et al. (2020). Our results indicated that a total of 35–42 days were a critical kpiB threshold for all considered treatments (see Fig. 5).

More generally, our study highlights advantages of AC-123 treatment compared to AC-135; superiority of IDAC-123 was shown for the first time. Based on RCTs (Schaich et al. 2011; Thomas et al. 2011; Burnett et al. 2013), no convincing evidence exists that Ara-C regimens at 3 g/m^2^ are more effective than those at intermediate-dose levels of 1–1.5 g/m^2^ (Döhner et al. 2017). Our results showed significantly shorter kpiL values for IDAC-123 compared to HDAC-123, and for IDAC-123 compared to IDAC-135. In all considered settings, G-CSF reduced WBC recovery times.

In the previous studies using trial-specific GSF-schedules (Jaramillo et al. 2017; Dumas et al. 2020), no difference in OS was observed between HDAC-123 and HDAC-135. Our mathematical model does not permit evaluation of OS directly. Also adversarial events like febrile infections, which are of high interest for clinical practice when comparing treatments, cannot be easily predicted. In our setting, predicting such an event would require both an extension of the model and additional data allowing us to learn the parameters coming from such an extension. However, in two clinical studies (Jaramillo et al. 2017; Dumas et al. 2020), it was already shown that the shorter exposure to low ANC/WBC values correlates with a reduction in AE. Therefore, it is a plausible assumption that similar statistics for adversarial events would arise in the considered variety of treatments.

In our setting, we can compare kpiB values based on the simulated dynamics of leukemic blasts, as discussed in the online supplement and shown in Fig. 4. These results suggest that the outcome of HDAC-123 and IDAC-123 as compared to HDAC-135 and IDAC-135, respectively, is not worse (but rather better) considering the impact of the chemotherapy schedule on the dynamics (and hence persistence) of leukemic blasts (kpiB).

Based on these results and emphasizing the risks of drawing conclusions from a numerical study, we summarize that IDAC-123 plus G-CSF administration is the most promising candidate for future post-remission AML therapy. The CC length should be well below 42 days. Shorter CC lengths are possible with the expected reduced kpiL values owing to the recommended IDAC-123 plus G-CSF treatment.

### Risks of drawing conclusions from a numerical study

There are justified concerns related to the predictions based on mathematical models. The data used to train the models are sparse in comparison to the huge datasets often used in machine learning. The longitudinal data for each digital twin comprised in median 24 WBC observations from up to 3 CC. For 65 patients, this “lots of little data” was enough for a robust training of the nonlinear mathematical model as discussed in Jost et al. (2019) and the standard deviations for the six estimated model parameters are reported in the supplement. The coefficients of variation are below 37% for the five most relevant parameters. We have performed Monte Carlo simulations and could hence validate that the results are stable within these regions of uncertainty (data not shown).

Another concern is that for every digital twin, there is a mismatch between the clinical and predicted outcomes. Our study aimed to simulate a variety of different treatments. One major issue in machine learning is extrapolation beyond the training data. Thus, predictions of mathematical models are expected to perform poorly when different treatments are used compared to those for which the training data were acquired. However, the opportunities arising from being able to compare different treatments in a well-defined setting are huge. Therefore, we designed our study to address this challenge. First, we used a physiological, mechanistic modeling approach. Such models do not possess the universal approximation property of neural networks: thus, we cannot expect to detect completely unknown physiological mechanisms. However, by modeling medical knowledge concerning myelosuppression or pharmacokinetics of Ara-C, we obtained an a priori interpretable model with transparent functional dependencies between the estimated model parameters and simulated outcomes. More importantly, such models are known to perform better when extrapolated to inputs beyond the training data. Second, we fixed the doses of Ara-C and lenograstim to those present in the training data (1 g/m^2^ or 3 g/m^2^ and $263\,\upmu {\text {g}}$, respectively). To avoid including Ara-C dosage as an additional variable in the model, we seperated patients treated with 1 g/m^2^ from patients treated with 3 g/m^2^. Although this restricts the results of the study to these values, we expect them to be more reliable, since the pharmacokinetic submodel cannot be identified owing to a lack of drug concentration measurements in the available training dataset. Third, we applied double cross-validation of the results by additionally comparing key performance indicators to those of independent clinical studies (see Fig. 2).

### Comparison of the cohorts in the double cross-validation

Certain differences exist in the specifics of the HDAC studies used for the double cross-validation method. While the median age of the cohorts in the studies by Jaramillo et al. (2017) and Dumas et al. (2020) varied between 41 and 50 years, the median age of the IDAC-virtual cohort was 57 years (Table 2). The multivariate Wei–Lin–Weissfeld test took age into consideration. When restricted to the Dumas et al. dataset which had the youngest population, age was not significant. However, for the Jaramillo et al. and the full data set, a significant impact was observed that could explain the absolute increase in the kpiL values for the IDAC-virtual cohort. We speculate that the superiority of AC-123 could also be considered in the elderly, and not only for the younger populations addressed in the previous studies (Jaramillo et al. 2017; Dumas et al. 2020).

Another important covariate to consider is G-CSF administration. While the clinical kpiL values for HDAC-123 and HDAC-135 were only available for pegfilgrastim administration, the training data of our mathematical model included lenograstim. Pegfilgrastim, the pegylated formulation of filgrastim, permits one-time administration (Sierra et al. 2008). In addition, pegfilgrastim was administered on different days (Jaramillo et al. 2017; Dumas et al. 2020). We argue that the similarity between the clinical and simulated results in Fig. 2 and Table 2 demonstrates that these differences were stochastically of minor importance when compared to the impact of AC-123 versus AC-135. On the positive side, the comparison of slightly different lenograstim and pegfilgrastim schedules can thus be interpreted as an indication of the robustness of our analysis.

### Biomarkers and key performance indicators

In clinical practice, ANC is preferred to WBC counts for treatment decision-making. Our mathematical model, and hence kpiL, is based on the WBC counts. This was owing to the better quality of the WBC data in our training set. For the ANC simulation, an identical mathematical model could be used, and the training would subsequently result in slightly different model parameters. Considering this analogy, the similarity of results with respect to ANC and WBC in the previous studies (Jaramillo et al. 2017; Dumas et al. 2020), and our focus on an analysis of the recovery times instead of individual clinical decision-making, we consider WBC as a valid choice for this study.

We also investigated the leukopenia times (number of days between the first WBC count $<1 000/\upmu {\text {L}}$ until recovery) and WBC nadir values as alternative indicators for leukopenia (neutropenia). Both the results were qualitatively identical, as shown in the online supplement. Thus, AC-123 and G-CSF administration reduces duration and severity of neutropenia compared to AC-135.

The key performance indicator kpiB denotes the impact of treatment on leukemic blasts. Mathematical modeling has been successfully applied to identify more efficient treatments in the previous studies. The Norton–Simons hypothesis and the derived new clinical standard of dense high-dose chemotherapy treatments for breast cancer are a prominent example (Michor and Beal 2015; Simon and Norton 2006). Our approach could be interpreted similarly. We also focused on the sensitivity of cancerous cells with respect to treatment choices. Our result that the AC-123 treatment is better in 94.2% of the considered CCs for kpiB compared to AC-135 which is consistent with the advantage of dense chemotherapy treatments postulated by Norton and Simons. In addition, we found an interesting non-trivial impact of G-CSF timing on kpiB (see Fig. 4) that should be investigated in future studies.

### Scientific implications

Several implications of our general approach exist as advocates for studies involving virtual cohorts of digital twins, which might complement or even replace preclinical animal studies. This concept can be transferred in a straightforward way to more complex cases of combination therapy or to other diseases. As a by-product, mathematical models can also be used for training the next generation of clinicians and for clinical decision-making support systems that foster personalized medicine based on individual dynamic responses.

## Supplementary Information

Below is the link to the electronic supplementary material.

**Supplementary information** Results of the parameter estimation and all fixed parameters are provided in the online supplement. All results should be reproducible up to numerical accuracy based on the differential equations, the specification of initial values, and these model parameters. (pdf 81929KB)

## Data Availability

Results of the parameter estimation and all fixed parameters are provided in the online supplement. All results should be reproducible up to numerical accuracy based on the differential equations, the specification of initial values, and these model parameters.
