# Supplementary material for: AML consolidation therapy: timing matters
Source: J Cancer Res Clin Oncol. 2023 Aug 3;149(15):13811–21. doi: 10.1007/s00432-023-05115-0 (PMC10590325; doi:10.1007/s00432-023-05115-0)
Supplement: Supplementary file 1 — Supplementary information Results of the parameter estimation and all fixed parameters are provided in the online supplement. All results should be reproducible up to numerical accuracy based on the differential equations, the specification of initial values, and these model parameters. (pdf 81929KB) [file 432_2023_5115_MOESM1_ESM.pdf]

AML Consolidation Therapy: Timing Matters

Adrian-Manuel Reimann, Enrico Schalk, Felix Jost, Dimitrios Mougiakakos, Daniela Weber, Hartmut Döhner, Christian Récher, Pierre-Yves Dumas, Marc Ditzhaus, Thomas Fischer, Sebastian Sager

Journal of Cancer Research and Clinical Oncology
Supplemental Material

This supplements complements the research article submitted to Journal of Cancer Research and Clinical Oncology with additional details. It makes relevant data available.

We provide details concerning data in Section 1. A detailed description of the mathematical model follows in in Section 2. Parameters for this model and details how they were derived are presented in Section 3. We discuss key performance indicators in detail in Sections 4 and Section 5. In Section 6 simulated trajectories for the complete virtual cohort and the most interesting treatment choices are shown.

1 Training Data

We trained 65 digital twins using longitudinal retrospective data. The data were obtained from the phase II AMLSG 12-09 randomized controlled trial (RCT; $n = 44$) and from clinical chart records from the Magdeburg University Hospital, Germany ($n = 21$). This study was approved by the ethics committee of the Magdeburg University Hospital, approval no. 124/15. The group was heterogeneous considering Ara-C administration, lenograstim administration, and age. Details are shown in Table 1.

Table 1: Numbers and age of patient subgroups in the training data

Data source	Magdeburg			Ulm		Sum
Ara-C	HDAC-123	HDAC-135	IDAC-135	HDAC-123	IDAC-123	all
#patients	1	11	10	18	25	65
with blast measurements	0	0	0	15	20	35
with lenograstim treatment	0	0	0	13	22	35
median age [y]	48	62	63	55.5	72	–
mean age [y]	48	62.8	54.9	51.6	70.5	–

The data set is identical to the one of a previous study [Jost et al., 2020], but for two patients who have been removed for reasons of data inconsistency. The data contains longitudinal patient-specific data, most importantly 1869 white blood cell (WBC) concentration measurements and 63 relative blast measurements from the bone marrow.

2 Mathematical Model

There are many different levels on which the dynamics of AML treatment can be modeled, see [Clairambault, 2009, Stiehl and Marciniak-Czochra, 2012, Brady and Enderling, 2019, Chulián et al., 2022] for further references. In [Jost et al., 2020], a differential equation model was presented that combines four submodels for hematopoiesis [Friberg et al., 2002, Jost et al., 2019], pharmacokinetics and -dynamics of cytarabine [Jost et al., 2019], leukemic cells [Stiehl et al., 2018], and G-CSF [Jost et al., 2020]. This comprehensive model allows to study in silico the impact of different treatment protocols on WBC (and hence neutropenia depth and length) and on the leukemic blasts.

Figure 1: A visualization of the complete differential equation model

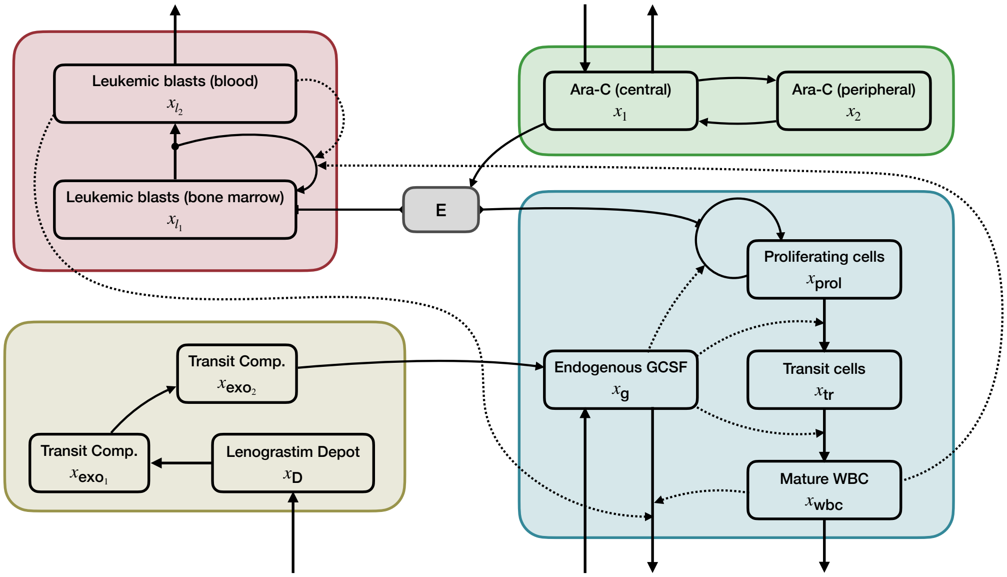

The differential states and functional relations between them are visualized in Figure 1. Shown are differential states x and the pharmacody-

namics E. Four submodels have been coupled and are indicated in different colors. The myelosuppression compartment model [Friberg et al., 2002] plus endogeuous G-CSF is shown in blue, the leukemic blast compartment model [Stiehl et al., 2018] in red, the pharmakokinetics of cytarabine [Jost et al., 2019] in green and a pharmacokinetics model for lenograstim in yellow. Functional relationships are indicated by arrows connecting the compartments.

The full system of differential equations is

$$\dot{x}_1 = k_{21}x_2 - (k_{10} + k_{12})x_1 + \frac{u_c \text{BSA}}{\text{dur}_c} \tag{1}$$

$$\dot{x}_2 = k_{12}x_1 - k_{21}x_2 \tag{2}$$

$$\dot{x}_{prol} = -\left(\frac{x_g}{B_g}\right)^{\beta} k_{tr}x_{prol} + \left(\frac{x_g}{B_g}\right)^{\gamma S_{x1}} k_{tr}(1-E)x_{prol} \tag{3}$$

$$\dot{x}_{tr} = \left(\frac{x_g}{B_g}\right)^{\beta} k_{tr}(x_{prol} - x_{tr}) \tag{4}$$

$$\dot{x}_{wbc} = \left(\frac{x_g}{B_g}\right)^{\beta} k_{tr}x_{tr} - k_{wbc}x_{wbc} \tag{5}$$

$$\dot{x}_g = k_{in} - k_{out}x_g \tag{6}$$

$$\dot{x}_D = -k_{a1}x_D + \frac{u_l 1000}{V_g \text{dur}_l} \tag{7}$$

$$\dot{x}_{exo_1} = k_{a1}x_D - k_{a2}x_{exo_1} \tag{8}$$

$$\dot{x}_{exo_2} = k_{a2}x_{exo_1} - k_{a2}x_{exo_2} \tag{9}$$

$$\dot{x}_{l1} = (2a_1k_{lc} - 1)p_1x_{l1} - p_1Ex_{l1} \tag{10}$$

$$\dot{x}_{l2} = 2(1 - a_1k_{lc})p_1x_{l1} - d_2x_{l2}. \tag{11}$$

with parameter-dependent initial values specified in Table 2

Table 2: Initial values for all differential states

**States**	**Initial values**
$x_1, x_2, x_{\text{exo1}}, x_{\text{exo2}}, x_D$	0
$x_{\text{prol}}, x_{\text{tr}}$	$(B\ k_{wbc})/k_{tr}$
x_{wbc}	B
x_g	B_g
x_{l1}	x^0_{blasts}
x_{l2}	$B/99$

and algebraic variables describing the pharmacodynamics of cytarabine E, a term S_{x_1} to modeling a secondary effect of cytarabine introduced and discussed in [Jost et al., 2019], terms k_{in} and k_{out} modeling the interactions in the G-CSF compartment, and a term k_{lc} modeling the influence of WBC counts on the proliferation of leukemic blasts [Stiehl et al., 2018] as follows:

$$E = \text{slope } \log\left(\frac{x_1}{V_c MM_{AraC}} + 1\right) \tag{12}$$

$$S_{x_1} = 1 + \log\left(\frac{x_1}{V_c MM_{AraC}} + 1\right) \tag{13}$$

$$k_{in} = (k_{e,g} + k_{ANC}B)B_g + k_{a2}x_{exo_2} \tag{14}$$

$$k_{out} = k_{e,g} + k_{ANC}(x_{wbc} + x_{l2}) \tag{15}$$

$$k_{lc} = \frac{1}{1 + c_1 x_{wbc} + c_2 x_{l2}}. \tag{16}$$

The mathematical model itself has already been used in previous studies [Jost et al., 2019, Jost et al., 2020]. We refer to them for a discussion of modeling assumptions and alternatives and a comprehensive literature survey.

3 Parameter Estimation

For most of the differential states in (1-11) no measurements are available, with the exception of WBC counts (x_{wbc}) and very few relative leukemic blast measurements. As a consequence, most of the model parameters had to be fixed to values from the literature. Table 3 gives an overview.

Table 3: List of all non–personalized model parameters

**Constant**	**Unit**	**Value**	**Description**
**PK model of Ara-C**			
k_{10}	$1/day$	98.2920	Elimination rate of Ara-C
k_{12}	$1/day$	2.6616	Distribution rate of Ara-C
k_{21}	$1/day$	12.8784	Distribution rate of Ara-C
Volume V_c	L	26.5554	Volume of central compartment
MM_{AraC}	g/mol	243.2170	Molecular mass of Ara-C
dur_c	day	0.1250	Infusion time
**PD model of WBCs and leukemic blasts**			

k_{wbc}	$1/day$	2.3765	Death rate of circulating WBCs
β	-	0.2340	Feedback of G-CSF on transit time
B_g	ng/L	24.4000	Endogenous G-CSF steady state
$k_{e,g}$	$1/day$	0.5920×24	Non-specific elimination rate constant
k_{ANC}	$1/day$	5.6400×24	Neutrophil-dependent elimination rate
a_1	-	0.8750	Probability of self-renewal
p_1	$1/day$	0.1000	Leukemic cell proliferation rate
d_2	$1/day$	2.3000	Leukemic cell death rate
c_1, c_2	$L/10^9$	0.0100	G-CSF quasi steady-state feedback scaling factor
**PK model of lenograstim**			
k_{a2}	-	$\frac{10}{3}k_{a1}$	2. absorption rate of lenograstim
V_g	L	14.5	Volume of distribution
dur_l	day	0.0007	Infusion time
BSA	m^2	2	Body surface area

For a personalization of the mathematical model, we used the longitudinal data to estimate the model parameters specified in Table 4.

Table 4: List of all personalized model parameters

**Parameter**	**Unit**	**Description**
k_{a1}	$1/day$	Absorption rate of lenograstim
k_{tr}	$1/day$	Transition rate
γ	–	Feedback speed of G-CSF on WBCs
slope	$L/\mu mol$	PD effect of Ara-C on WBCs
B	$10^9/L$	Baseline of WBC count
x^0_{blasts}	$10^9/L$	Relative number of blasts at start of consolidation therapy

Using a population parameter estimation (nonlinear mixed-effects modeling) approach with the software NONMEM 7.5.0, we obtained six individual model parameters for each patient. For all estimated parameters we have both patient-specific estimations and fixed effect. The fixed effects are listed in Table 5. They can be interpreted as a general prediction of the (population) parameter. The residual error was 0.199. Note that the initial values for $x_{l1} = x^0_{blasts}$ were calculated involving a heuristic function to translate the relative blast counts to absolute numbers. Furthermore, the formula $x^0_{blasts} = \hat{x} - 0.005(B\ k_{wbc})/k_{tr}$ was applied to connect estimated numbers $\hat{x}$ with another modeling assumption. See [Jost et al., 2020] for details.

Table 5: Results of the nonlinear mixed-effects modeling and numerical solution with NONMEM 7.5.0

B	k_{tr}	slope	γ	k_a	x^0_{blasts}
**Fixed Effect Prediction**					
4.78	0.202	6.94	0.707	4.53	0.00458
**Interindividual Variability CV%**					
37.1	23.6	32.1	19.8	81.7	11.4

Finally, we provide the personalized model parameters for all 65 patients included in our study. All parameters are given with a consistent number of digits. This is not due to significance but rather due to readability and consistency with the literature values provided in Table 3.

Table 6: Patient-specific estimated model parameters

**Patient id**	**B**	$\boldsymbol{k_{tr}}$	$\boldsymbol{\gamma}$	**slope**	$\mathbf{k_a}$	$\boldsymbol{x^0_{blasts}}$
1	7.9486	0.25944	0.65448	6.6155	3.2428	1.6867
2	2.8166	0.24445	0.52384	7.6323	9.0704	4.2797
3	10.592	0.25506	0.74528	9.6581	1.9137	4.7626
4	3.5361	0.24263	0.67855	7.5927	5.0817	2.5351
5	4.8955	0.20583	0.62789	5.4085	6.2395	1.4801
6	4.5859	0.21625	0.70575	7.5677	4.2518	1.9014
7	5.4688	0.24489	0.57731	7.2270	4.1366	1.8369
8	3.0416	0.18571	0.68770	6.3089	6.3185	1.2505
9	5.3121	0.30312	0.65462	7.4970	2.1573	5.2653
10	3.4184	0.25135	0.57087	7.4679	4.8991	1.0951
11	8.0523	0.16385	0.93809	11.590	1.9737	3.8741
12	2.1501	0.19919	0.67406	9.4983	4.1113	2.7085
13	5.9667	0.18948	0.65151	5.6163	4.5824	0.2803
14	3.4979	0.27748	0.54717	5.8753	8.1764	2.3349
15	4.7392	0.25209	0.56108	5.8816	5.9524	1.5701
16	5.3869	0.20788	0.60090	12.445	3.9281	8.8081
17	4.2011	0.23157	0.71396	6.8993	5.1013	1.0670
18	4.8346	0.28311	0.54539	6.2418	6.4825	1.2162
19	4.2657	0.19235	0.74215	5.8187	4.3950	0.4692
20	7.0345	0.25000	0.71213	5.6554	5.0671	3.7435

Table 6: Patient-specific estimated model parameters

Patient id	B	k_{tr}	γ	slope	k_a	x^0_{blasts}
21	8.3913	0.25708	0.60111	4.1656	4.0396	0.4120
22	8.1884	0.30259	0.57469	6.7056	4.6485	3.3539
23	6.5801	0.23684	0.81094	8.1394	3.3494	3.5222
24	7.3105	0.25661	0.88452	9.9849	3.2972	2.2347
25	5.8967	0.13113	0.56008	7.1150	4.1098	0.3608
26	8.2972	0.19978	0.58588	6.8880	2.7974	0.4445
27	5.6396	0.15433	0.95078	9.7500	3.0166	2.9667
28	2.7016	0.24733	0.55248	10.361	6.7852	4.2782
29	3.7000	0.21510	0.72975	9.1689	3.5145	2.3707
30	5.6541	0.23721	0.82594	10.208	2.2576	5.4032
31	5.2017	0.22406	0.82755	9.7669	2.8261	2.7652
32	4.0655	0.16873	0.91295	7.1931	5.4911	0.9803
33	4.7514	0.16954	0.79004	4.7579	6.5324	0.4055
34	3.4610	0.17505	0.79958	9.3623	6.3286	2.2810
35	3.4718	0.15294	1.05930	9.9026	2.4458	3.5586
36	5.2309	0.32344	0.89942	5.9028	9.3148	3.3537
37	9.5609	0.32892	0.61605	8.6815	2.2489	3.0188
38	5.0640	0.26599	0.79128	6.6957	6.0160	6.3722
39	10.139	0.27426	0.71329	10.003	3.3266	7.9299
40	5.5669	0.19500	0.64255	8.1875	3.5311	1.3349
41	4.3203	0.18957	0.79576	7.3622	4.4171	0.7779
42	4.5405	0.23121	0.60351	7.6093	4.6768	1.9716
43	4.4084	0.22807	0.67270	9.2018	4.8867	3.2528
44	5.2061	0.15261	0.79031	7.8732	3.4211	1.1890
45	4.9365	0.18405	0.64306	3.6496	11.567	6.2983
46	6.0840	0.17222	0.80826	4.9991	9.4007	23.698
47	6.2352	0.20943	0.75407	8.9540	3.2550	3.4045
48	5.1978	0.19175	0.67417	3.4368	5.3916	0.0021
49	5.4062	0.18606	0.74200	9.1736	3.5217	3.2048
50	5.6288	0.21284	0.62145	4.0890	7.5715	2.5029
51	6.6960	0.17720	0.61307	5.9185	4.2019	1.3424
52	3.9256	0.17039	0.66297	5.0101	7.2501	1.6524
53	4.8942	0.21438	0.58426	4.0313	4.9581	0.0394
54	4.2305	0.16222	1.14650	12.207	1.5185	0.0237
55	6.4670	0.17902	0.63684	7.4602	3.2723	1.1549

Table 6: Patient-specific estimated model parameters

**Patient id**	**B**	k_{tr}	γ	**slope**	$\mathbf{k_a}$	x^0_{blasts}
56	3.1446	0.16629	0.70567	6.7460	4.2348	0.2009
57	2.2922	0.13701	0.76650	6.6364	6.5720	0.7695
58	5.2768	0.13701	0.75130	10.684	2.9764	2.4160
59	3.3872	0.17145	0.78383	6.4929	5.2151	0.9589
60	5.7936	0.22091	0.76722	7.8194	3.7277	2.8695
61	3.5002	0.15326	0.69783	4.2491	7.1642	0.4473
62	3.6137	0.14057	0.78554	5.6245	5.5405	0.6721
63	8.3834	0.17254	0.78270	10.991	2.2811	4.1653
64	3.6361	0.17476	0.55405	6.8366	5.9258	1.6388
65	6.4818	0.14356	0.92028	6.2728	3.4485	0.9695

Concerning the numerical simulation studies, we obtained stable results as manifested by the smooth and plausible distributions of the median values in Figures 3, 4, and 5 in the main manuscript. Thus, even if the predictions of digital twins differ from the possible outcome that a treatment would have caused in a particular patient, the stochastic consideration of a cohort seems to balance out the individual differences. While our virtual cohorts comprised only 65 patients, we considered 65 130 CCs. Additionally, we performed proof-of-concept studies using Monte-Carlo simulations. Since the process of drawing individual parameters from a population parameter distribution is not trivial from a stochastic point of view, we did not include these results here, but only state that the main results were stable with respect to the underlying virtual cohort.

4 Key Performance Indicators Leukopenia

The goal of our study was to compare different AML treatments. Three criteria to be taken into account are leukopenia (neutropenia), leukemic blasts, and overall amount of administered drugs. For leukopenia, we mainly considered and discussed the WBC recovery time in the main article. However, our approach allows also for an easy evaluation of alternative performance indicators.

While *WBC recovery time* is defined by the number of days between start of a CC and recovery of WBC $> 1000/\mu L$, the *leukopenia duration* is given by

the number of days between the first time that WBC drop below the critical threshold and the first time they recover back $> 1000/\mu L$. This duration is by definition shorter than WBC recovery time and can be expected to be a better indicator for the time a patient is at highest risk. Figure 2 shows the heatmap in analogy to Figure 3 of the manuscript. As can be observed, the results are qualitatively similar to Figure 3 in the main manuscript.

Figure 2: Like Figure 3 of the main manuscript, but evaluated for leukopenia duration in days. The heatmaps indicates the same optimal regions for administration of G-CSF in the top left corner of all four subplots. AC-123 treatment resulted in shorter leukopenia durations compared to AC-135.

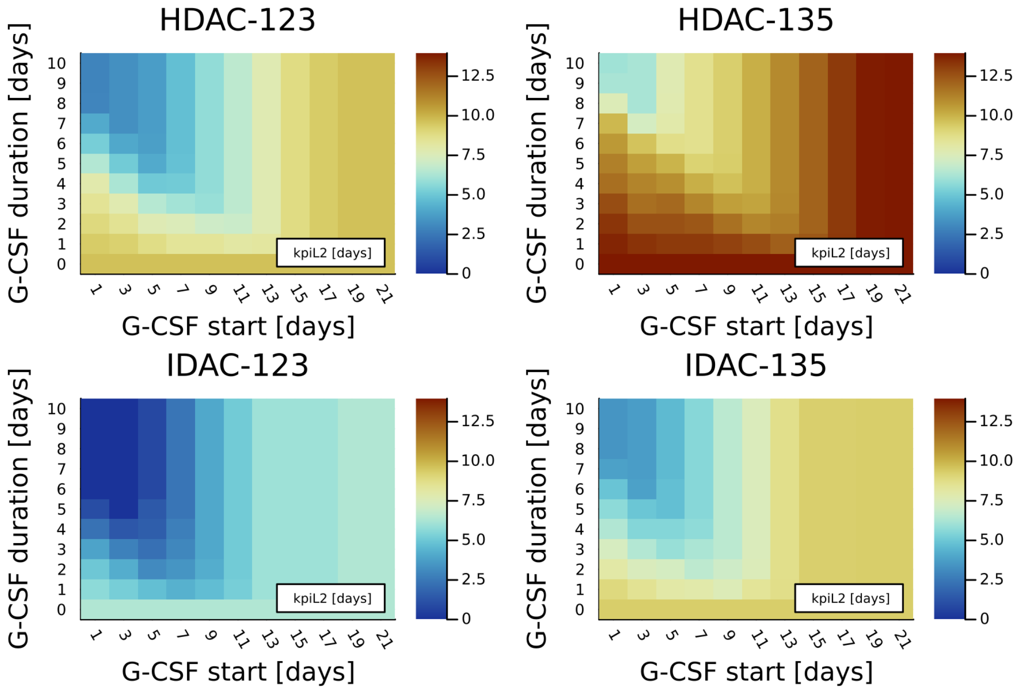

Not only the duration, but also the severity of a leukopenia could impact the risk of infectious complications. Figure 3 shows the minimum WBC value during the first CC.

Figure 3: Like Figure 3 of the main manuscript, but evaluated for WBC nadir. Again, the heatmaps indicate the same optimal regions for administration of G-CSF in the top left corner of all four subplots. AC-123 treatment resulted in higher WBC nadirs compared to AC-135, indicating a less severe leukopenia (note that here a higher value is the better result).

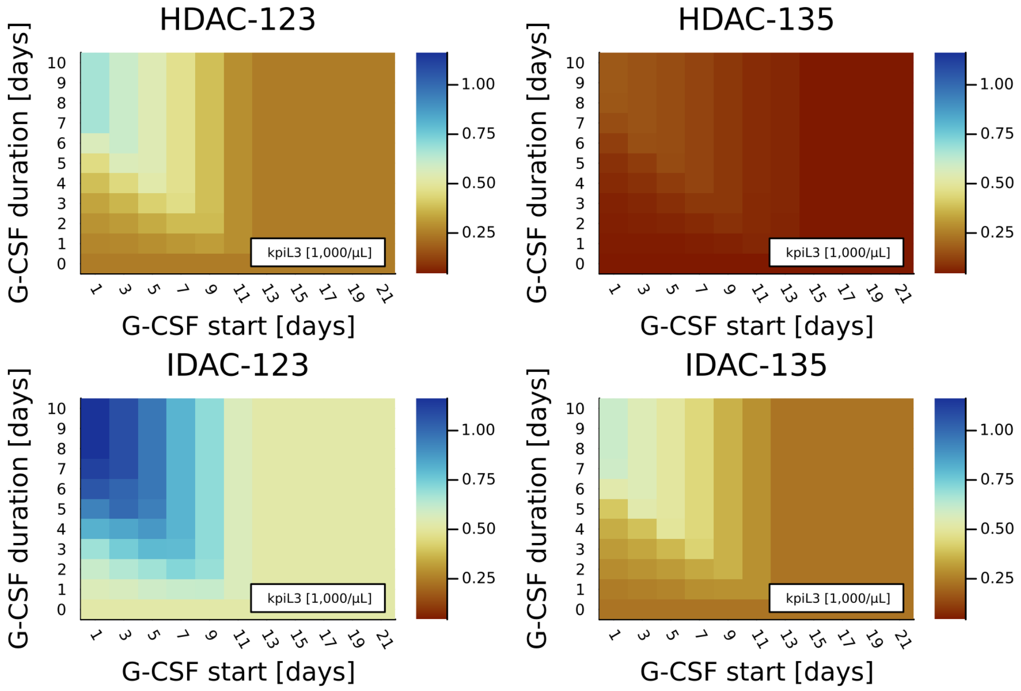

The results show that AC-123 leads to shorter **and** less severe periods of leukopenia. A similar statement is true for intermediate-dose in comparison to high-dose cytarabine administration. In all settings, an advantage of a long and early G-CSF administration could be observed.

An earlier administration of G-CSF overlapping with Ara-C administration is discouraged. There is clinical evidence for a strong increase in WBC recovery time in this case [Ortiz et al., 1993] that we could verify in our simulations (data not shown).

5 Key Performance Indicators Blasts

The second criterium we took into account when evaluating different treatments was the impact on the leukemic blasts in the bone marrow. This is

very important to get a better understanding of balancing the conflicting therapy goals (avoiding severe leukopenia vs. reducing leukemic blasts).

In clinical practice it is very challenging to assess the absolute number of leukemic blasts. Bone marrow assessments are intrusive and yield uncertain relative measurements. Patients in consolidation therapy are in complete remission. In routine practice, medullary blasts in patients in complete remission are often normal immature cells. Thus, clinical decisions on how to avoid relapse have to be made without knowledge of absolute leukemic blast numbers and rely on measurable residual disease or the OS of previous clinical studies. The situation is completely different in numerical studies, because these allow an exact "observation" of the predicted values. But also in our context of training the mathematical model the sparsity and uncertainty of blast measurements was a challenge. For some of the 65 patients there were 1 or 2 measurements of BM blasts% in our data. We used a heuristic measurement function to transfer the relative blast count into an absolute number [Jost et al., 2020] and estimated model parameters x^0_{blasts} with this data for all the patients. For patients without available measurements the value was set to the average fixed effect or population parameter value of x^0_{blasts}. In summary, the initial values x^0_{blasts} are very poorly approximated and uncertain. That would be a problem if we wanted to make individual decision-support or OS prediction.

For our focus on dynamics, however, this uncertainty is more or less irrelevant. By modeling the most relevant physiological processes of blast proliferation, we were able to predict absolute leukemic blast numbers for all digital twins. By considering the ratio between absolute numbers at the end and beginning of treatment, which can be easily extracted from the numerical solution, we could focus on the question how much a treatment decreases (or increases) the absolute number of leukemic cells in comparison to other treatment choices.

This is a well-established approach in mathematical oncology. For example, the Norton-Simon hypothesis "Chemotherapy success is proportional to the growth rate of proliferating cancerous cells" and an investigation of the right hand side of the differential equation describing cancerous cell proliferation led to the insight that early, dense, high-dosage chemotherapy treatments for breast cancer are to be preferred, a triumphal success for mathematical modeling with huge clinical impact [Michor and Beal, 2015]. Norton and Simon considered Gompertz growth for the cells. Their analysis showed that timing is crucial independent of the absolute number of cancerous cells.

In this line of thought we decided to analyse $\frac{x_{l1}(t)}{x_{l1}(0)}$ and to use $\frac{x_{l1}(T)}{x_{l1}(0)}$ at the end of consolidation cycle times T as key performance indicator kpiB.

The submodel for leukemic blasts [Stiehl et al., 2018] is more involved than simple Gompertz or exponential growth and considers interactions between the numbers of mature WBC and leukemic blasts. Thus, an analytical investigation is beyond the scope of this paper. Yet, the growth of leukemic cells is approximately exponential, as can be observed by looking at simulated trajectories (see next Section). By looking at the analytical solution

$$x_{l1}(t) = x^0_{blasts}\, e^{ct}$$

of the simplified linear differential equation

$$\dot{x}_{l1}(t) = c\, x_{l1}(t), \quad x_{l1}(0) = x^0_{blasts}$$

for a constant value c one observes that the ratio $\frac{x_{l1}(T)}{x^0_{blasts}} = e^{cT}$ is independent of the practically difficult to assess value $x_{l1}(0) = x^0_{blasts}$, but depends on the value c which can be linked to different treatment choices. We expected a similar behavior for our mathematical model. Thus, even if the simulated absolute leukemic blast counts are inaccurate in comparison to real patient data owing to the few available relative leukemic blast count measurements, this approach leads to a sensitivity of kpiB with respect to different treatments, but an in-sensitivity with respect to the unknown absolute leukemic blast numbers. Another advantage of this choice of kpiB is the straightforward interpretability. By design a value greater than 1 would indicate an increase of leukemic blasts, otherwise a decrease.

To investigate this numerically, we simulated with strongly modified different initial values x^0_{blasts} for the leukemic blasts in the bone marrow and depicted the results in Figure 4. For illustration, we considered one exemplary patient (the first one with patient id 1). We chose HDAC-123 and lenograstim administration starting on day 8 for 5 consecutive days and a CC length of 42 days for the treatment. The estimated initial value $x^0_{blasts} = 1.6867$ of the patient was changed to factor $\cdot\, x^0_{blasts}$ for various values of factor, covering three orders of magnitude.

A comparison between $\frac{x_{l1}(t)}{x^0_{blasts}}$, the kpiB value in the top subplot, and $x_{l1}(t)$, the absolute number of leukemic blasts in the bottom subplot, indicates the expected strong sensitivity of the latter with respect to the chosen initial value. In contrast, the trajectories $\frac{x_{l1}(t)}{x^0_{blasts}}$ are almost identical despite the large variance in the initial value.

Figure 4: Impact of different initial values on the dynamics of kpiB and of leukemic blasts in the bone marrow

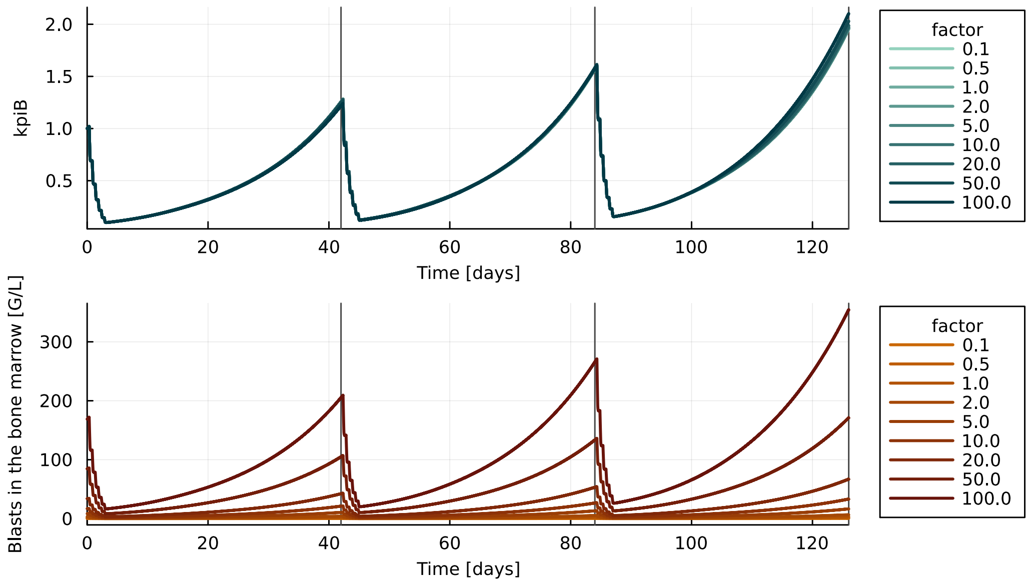

One also observes that the absolute leukemic blast numbers increase during consolidation therapy. This is reflected by kpiB values larger 1.0 and due to a comparitively long CC duration of 42 days. This is interesting, because in [Dumas et al., 2020] the statement "We found that a delay $\geq$ 40 days between HDAC cycles 1 and 2 was independently and significantly associated with poorer OS rate. We have no clear explanation for this intriguing result, which should be interpreted with caution and should prompt additional investigations. If confirmed, this could have important implications for routine practice." made a similar claim based on clinical outcome.

Figure 5 shows the results of a study focusing on four different treatments. One observes the different behavior of kpiB, dependent on the Ara-C administration and whether G-CSF is administered or not. The CC duration has been chosen as 35 days. This resulted in kpiB values < 1.0 for all four treatments, differing from the situation in Figure 4.

Figure 5: Impact of different treatments on the dynamics of kpiB and of leukemic blasts in the bone marrow

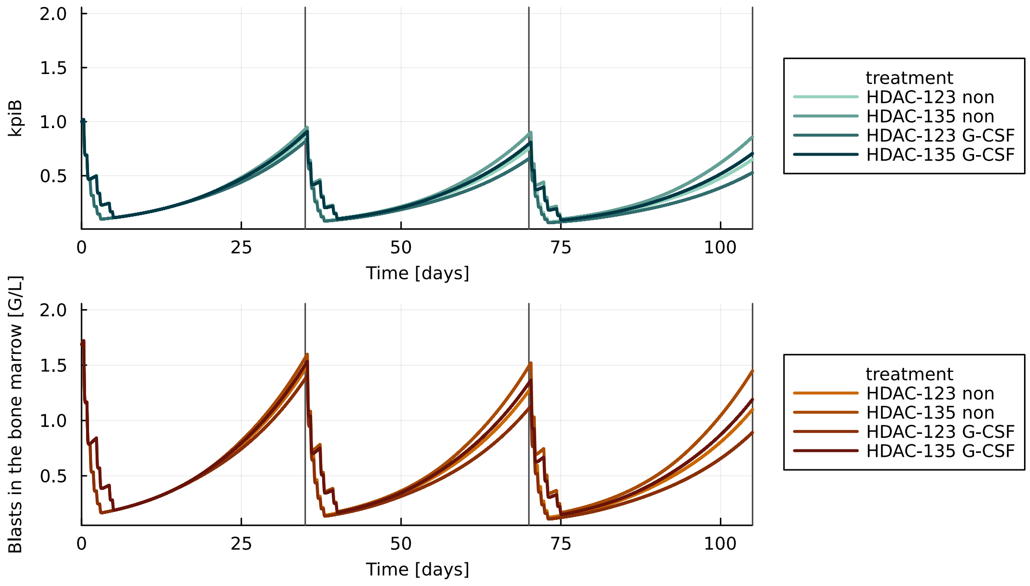

In summary, our key performance indicator kpiB has the property of insensitivity with respect to the typically unknown initial values and a sensitivity with respect to the treatment choices Ara-C and lenograstim administration and CC length.

6 Simulated trajectories

In the interest of detailed individual comparisons of treatment outcomes we provide plots for all 65 patients and the four treatments used in Figure 2 of the manuscript. The following 65 plots contain four subplots each, with the top two showing results for no G-CSF administration and the bottom two showing results for lenograstim administration for 5 consecutive days starting on day 8 for AC-123, and day 10 for AC-135. The subplots show the WBC count $x_{wbc}(t)$ and the kpiB value $\frac{x_{l1}(t)}{x_{l1}(0)}$ evolving over time, respectively. In all four subplots, one trajectory corresponds to HDAC-123 (or IDAC-123, depending on the dosage the digital twin was trained with) and one to HDAC-135 (or IDAC-135) treatment. Additionally, we provide numerical values for kpiL and kpiB for all three CCs in the legend of each subplot.

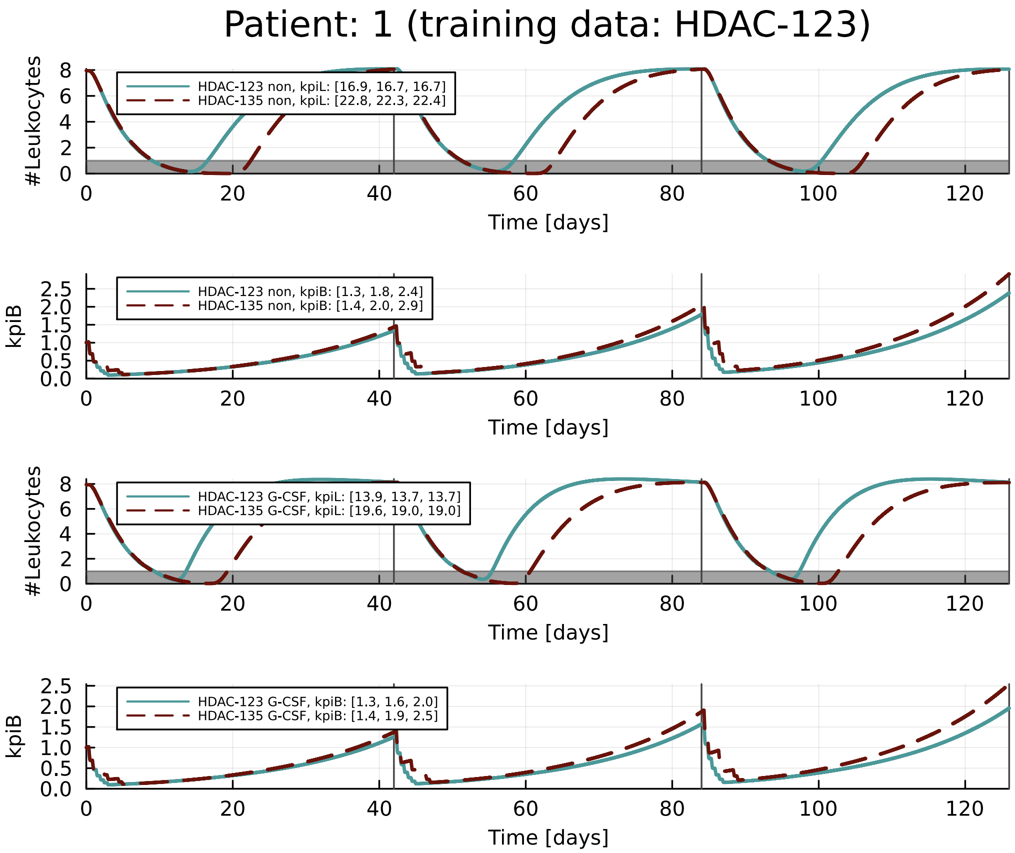

Patient: 1 (training data: HDAC-123)
HDAC-123 non, kpiL: [16.9, 16.7, 16.7]
HDAC-135 non, kpiL: [22.8, 22.3, 22.4]
HDAC-123 non, kpiB: [1.3, 1.8, 2.4]
HDAC-135 non, kpiB: [1.4, 2.0, 2.9]
HDAC-123 G-CSF, kpiL: [13.9, 13.7, 13.7]
HDAC-135 G-CSF, kpiL: [19.6, 19.0, 19.0]
HDAC-123 G-CSF, kpiB: [1.3, 1.6, 2.0]
HDAC-135 G-CSF, kpiB: [1.4, 1.9, 2.5]
#Leukocytes
kpiB
Time [days]

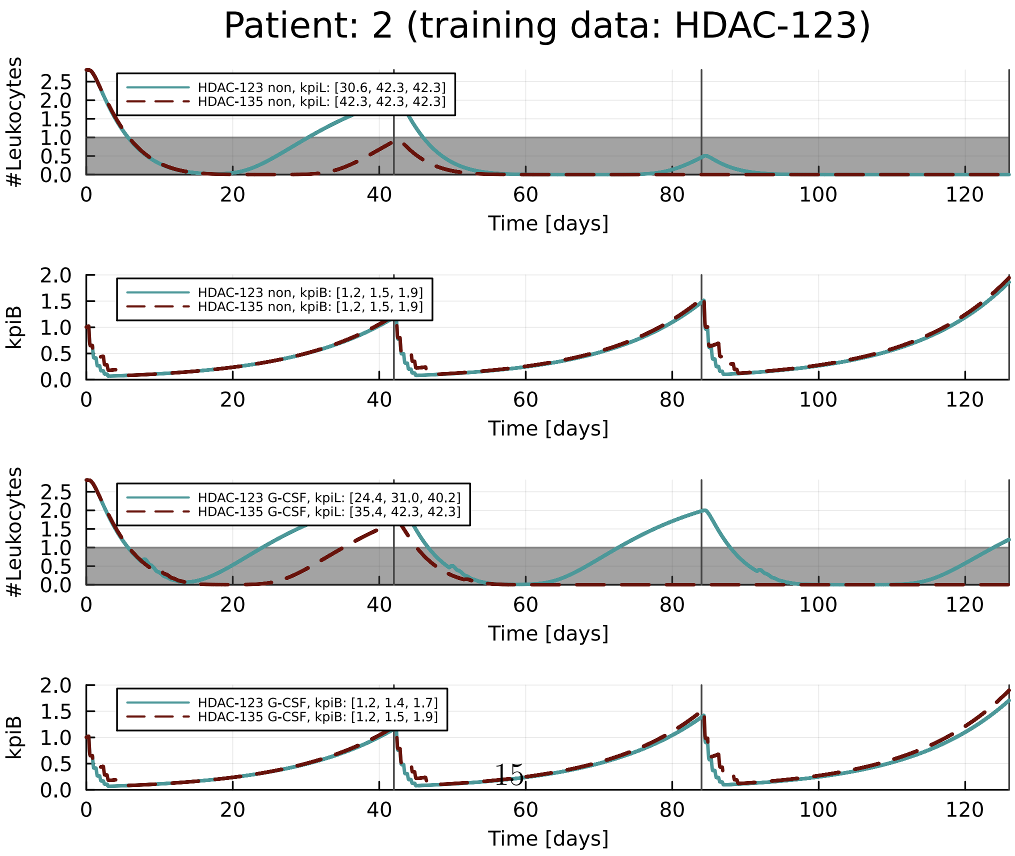

Patient: 2 (training data: HDAC-123)
HDAC-123 non, kpiL: [30.6, 42.3, 42.3]
HDAC-135 non, kpiL: [42.3, 42.3, 42.3]
HDAC-123 non, kpiB: [1.2, 1.5, 1.9]
HDAC-135 non, kpiB: [1.2, 1.5, 1.9]
HDAC-123 G-CSF, kpiL: [24.4, 31.0, 40.2]
HDAC-135 G-CSF, kpiL: [35.4, 42.3, 42.3]
HDAC-123 G-CSF, kpiB: [1.2, 1.4, 1.7]
HDAC-135 G-CSF, kpiB: [1.2, 1.5, 1.9]
#Leukocytes
kpiB
Time [days]

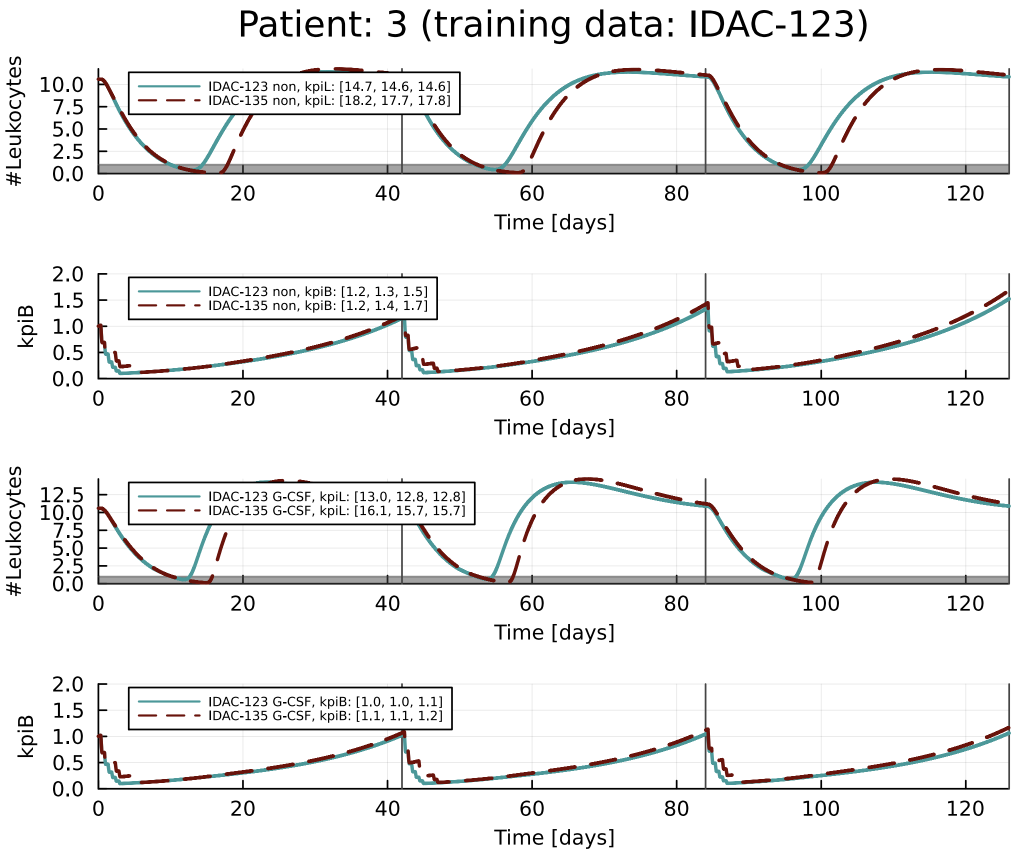
Patient: 3 (training data: IDAC-123)
IDAC-123 non, kpiL: [14.7, 14.6, 14.6]
IDAC-135 non, kpiL: [18.2, 17.7, 17.8]
#Leukocytes
Time [days]
IDAC-123 non, kpiB: [1.2, 1.3, 1.5]
IDAC-135 non, kpiB: [1.2, 1.4, 1.7]
kpiB
Time [days]
IDAC-123 G-CSF, kpiL: [13.0, 12.8, 12.8]
IDAC-135 G-CSF, kpiL: [16.1, 15.7, 15.7]
#Leukocytes
Time [days]
IDAC-123 G-CSF, kpiB: [1.0, 1.0, 1.1]
IDAC-135 G-CSF, kpiB: [1.1, 1.1, 1.2]
kpiB
Time [days]

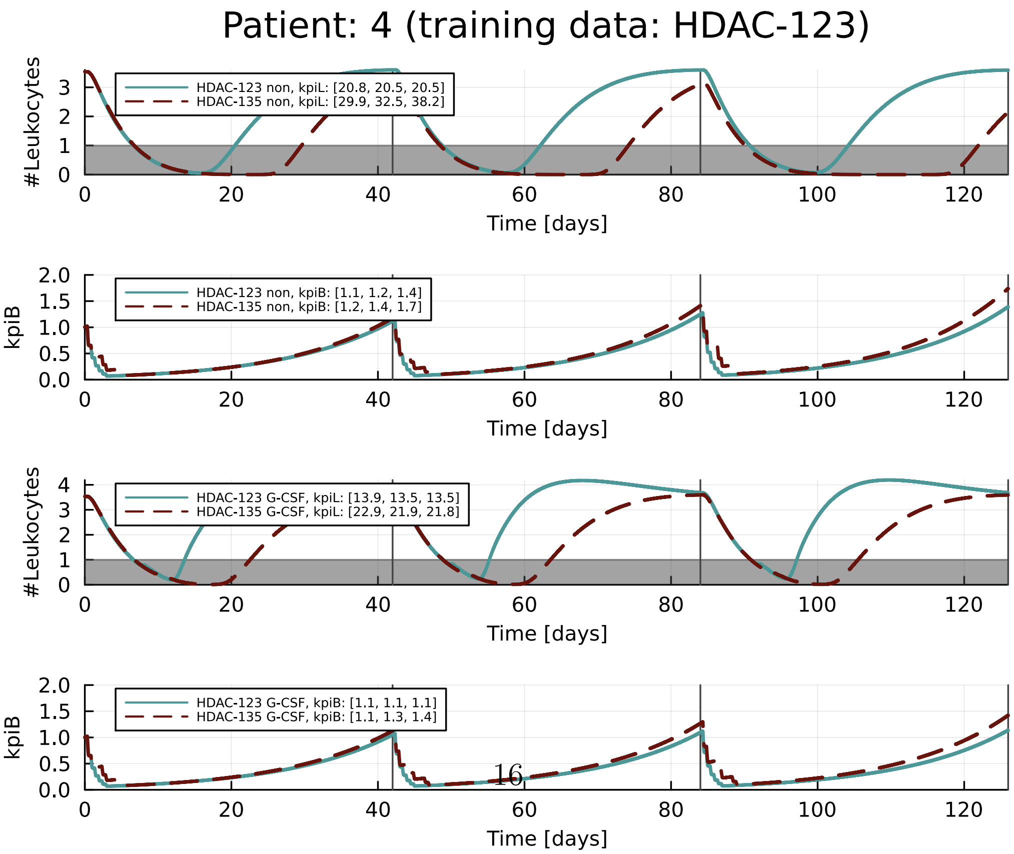
Patient: 4 (training data: HDAC-123)
HDAC-123 non, kpiL: [20.8, 20.5, 20.5]
HDAC-135 non, kpiL: [29.9, 32.5, 38.2]
#Leukocytes
Time [days]
HDAC-123 non, kpiB: [1.1, 1.2, 1.4]
HDAC-135 non, kpiB: [1.2, 1.4, 1.7]
kpiB
Time [days]
HDAC-123 G-CSF, kpiL: [13.9, 13.5, 13.5]
HDAC-135 G-CSF, kpiL: [22.9, 21.9, 21.8]
#Leukocytes
Time [days]
HDAC-123 G-CSF, kpiB: [1.1, 1.1, 1.1]
HDAC-135 G-CSF, kpiB: [1.1, 1.3, 1.4]
kpiB
Time [days]

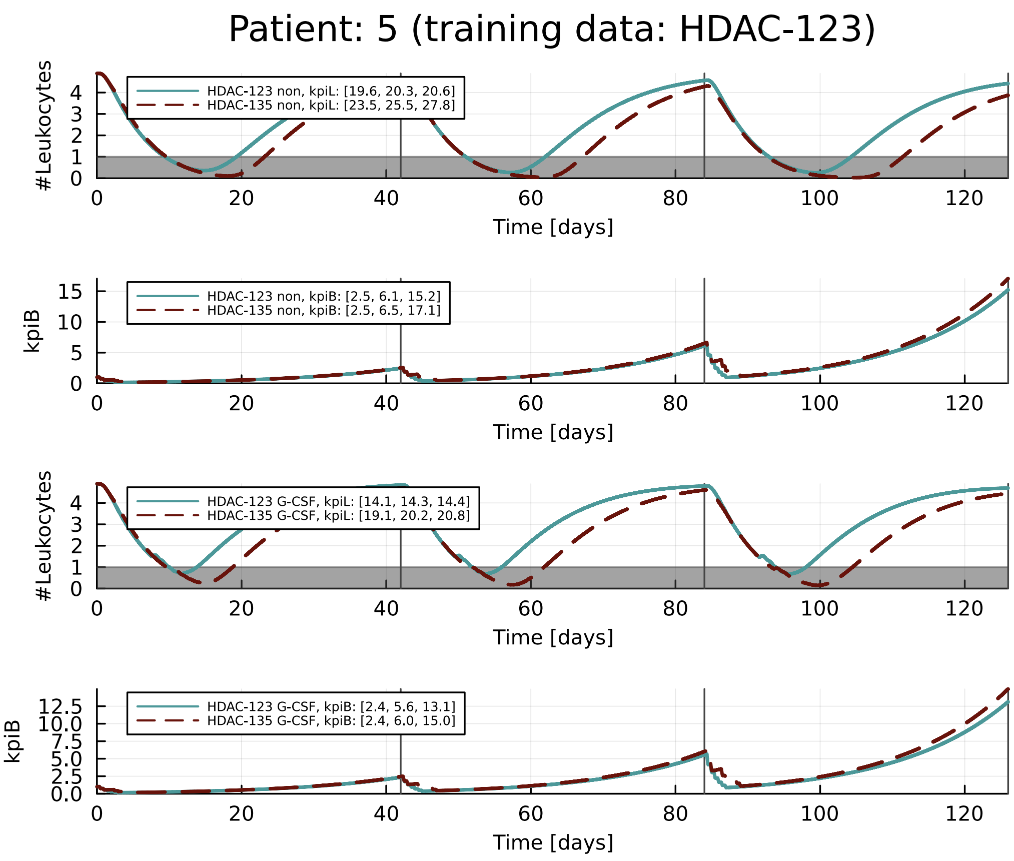
Patient: 5 (training data: HDAC-123)
HDAC-123 non, kpiL: [19.6, 20.3, 20.6]
HDAC-135 non, kpiL: [23.5, 25.5, 27.8]
HDAC-123 non, kpiB: [2.5, 6.1, 15.2]
HDAC-135 non, kpiB: [2.5, 6.5, 17.1]
HDAC-123 G-CSF, kpiL: [14.1, 14.3, 14.4]
HDAC-135 G-CSF, kpiL: [19.1, 20.2, 20.8]
HDAC-123 G-CSF, kpiB: [2.4, 5.6, 13.1]
HDAC-135 G-CSF, kpiB: [2.4, 6.0, 15.0]
#Leukocytes
kpiB
Time [days]

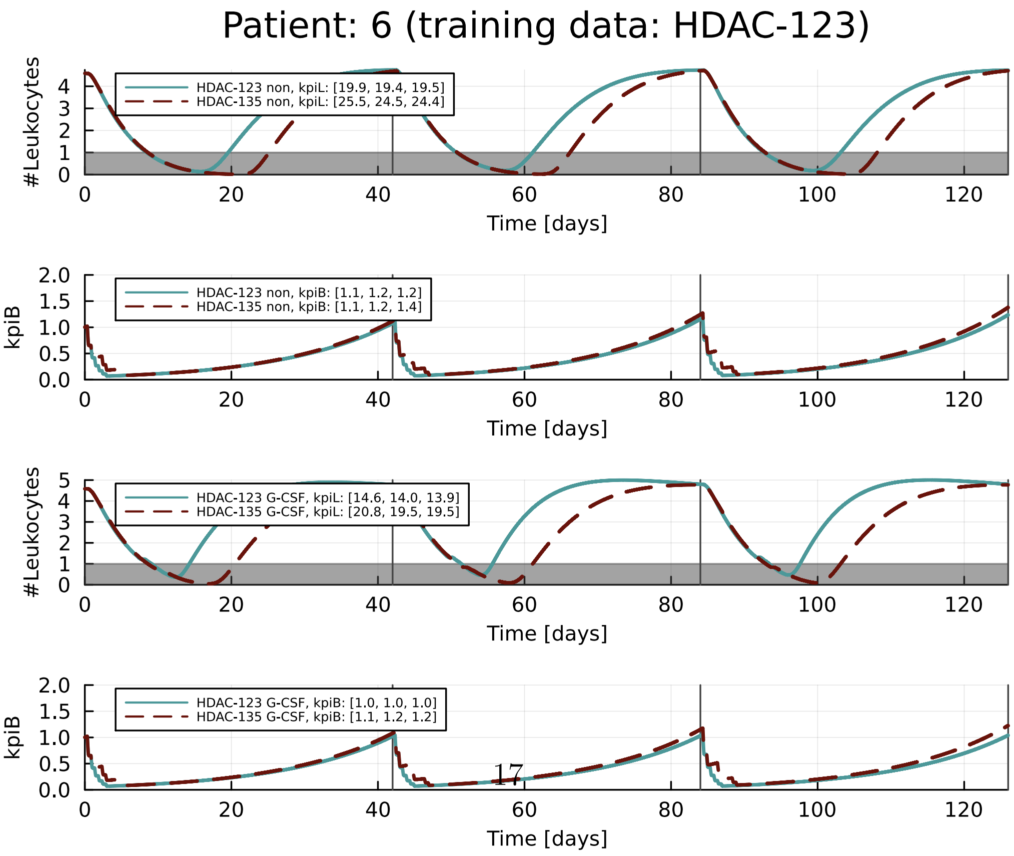
Patient: 6 (training data: HDAC-123)
HDAC-123 non, kpiL: [19.9, 19.4, 19.5]
HDAC-135 non, kpiL: [25.5, 24.5, 24.4]
HDAC-123 non, kpiB: [1.1, 1.2, 1.2]
HDAC-135 non, kpiB: [1.1, 1.2, 1.4]
HDAC-123 G-CSF, kpiL: [14.6, 14.0, 13.9]
HDAC-135 G-CSF, kpiL: [20.8, 19.5, 19.5]
HDAC-123 G-CSF, kpiB: [1.0, 1.0, 1.0]
HDAC-135 G-CSF, kpiB: [1.1, 1.2, 1.2]
#Leukocytes
kpiB
Time [days]

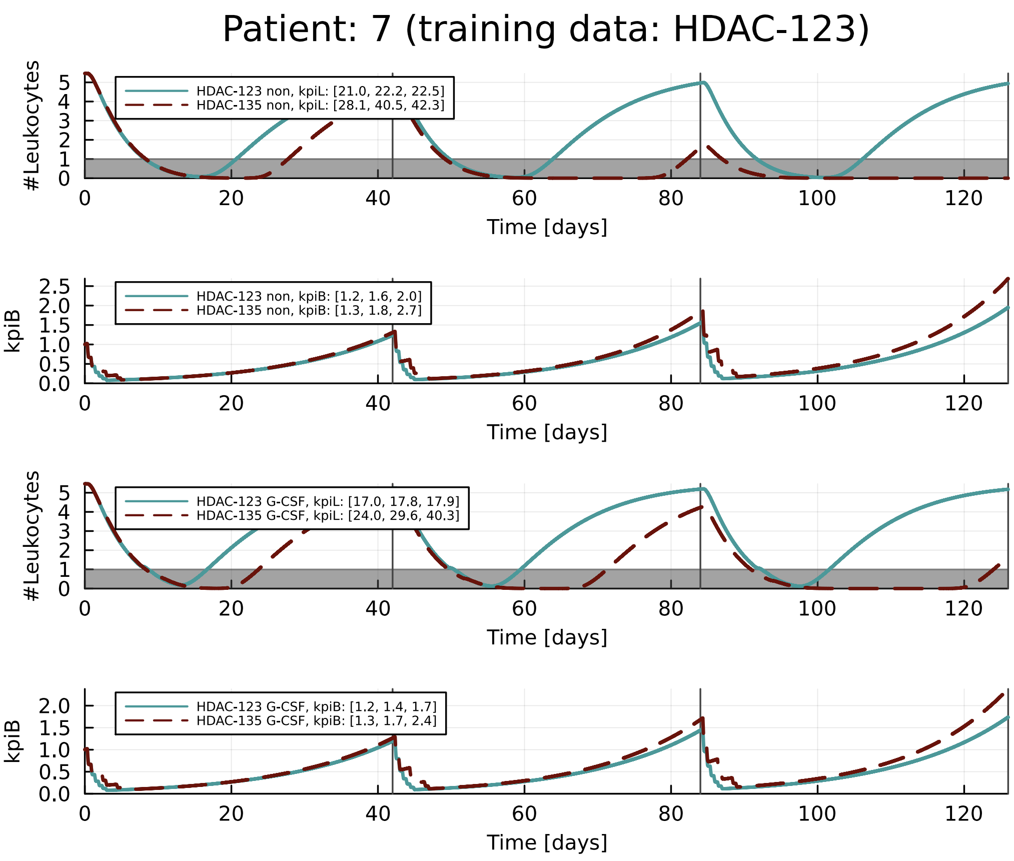
Patient: 7 (training data: HDAC-123)
HDAC-123 non, kpiL: [21.0, 22.2, 22.5]
HDAC-135 non, kpiL: [28.1, 40.5, 42.3]
#Leukocytes
Time [days]
HDAC-123 non, kpiB: [1.2, 1.6, 2.0]
HDAC-135 non, kpiB: [1.3, 1.8, 2.7]
kpiB
Time [days]
HDAC-123 G-CSF, kpiL: [17.0, 17.8, 17.9]
HDAC-135 G-CSF, kpiL: [24.0, 29.6, 40.3]
#Leukocytes
Time [days]
HDAC-123 G-CSF, kpiB: [1.2, 1.4, 1.7]
HDAC-135 G-CSF, kpiB: [1.3, 1.7, 2.4]
kpiB
Time [days]

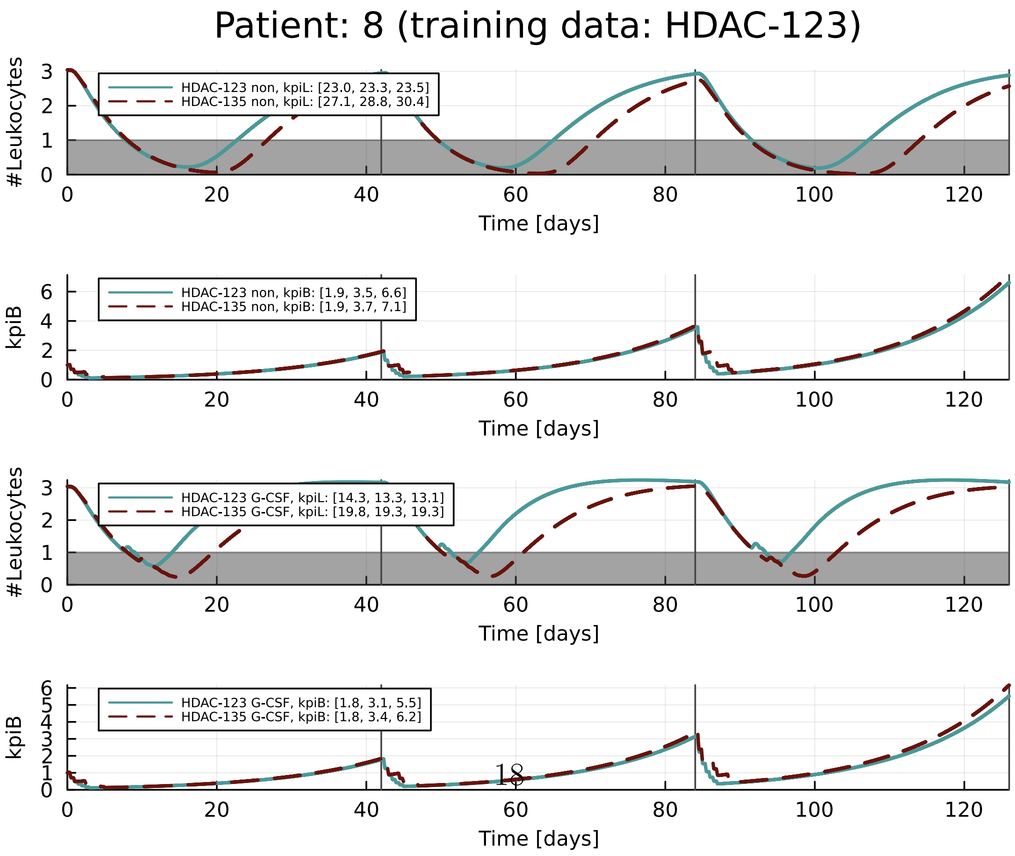
Patient: 8 (training data: HDAC-123)
HDAC-123 non, kpiL: [23.0, 23.3, 23.5]
HDAC-135 non, kpiL: [27.1, 28.8, 30.4]
#Leukocytes
Time [days]
HDAC-123 non, kpiB: [1.9, 3.5, 6.6]
HDAC-135 non, kpiB: [1.9, 3.7, 7.1]
kpiB
Time [days]
HDAC-123 G-CSF, kpiL: [14.3, 13.3, 13.1]
HDAC-135 G-CSF, kpiL: [19.8, 19.3, 19.3]
#Leukocytes
Time [days]
HDAC-123 G-CSF, kpiB: [1.8, 3.1, 5.5]
HDAC-135 G-CSF, kpiB: [1.8, 3.4, 6.2]
kpiB
Time [days]

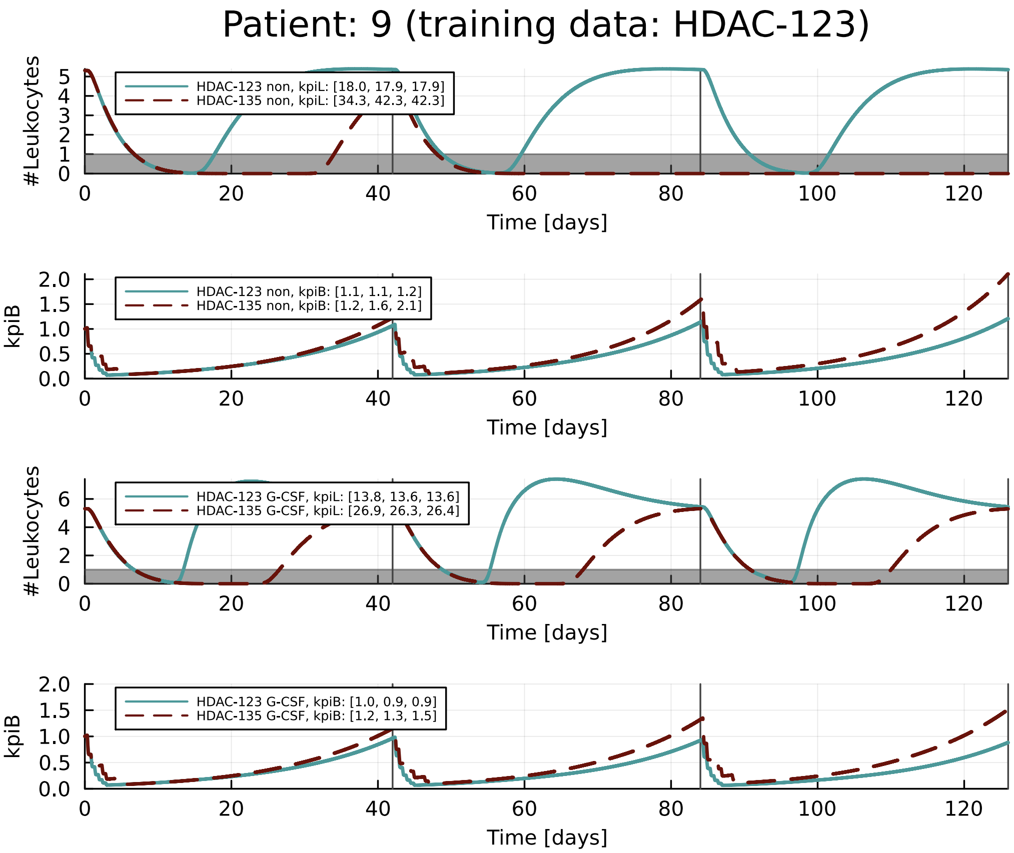
Patient: 9 (training data: HDAC-123)
#Leukocytes
HDAC-123 non, kpiL: [18.0, 17.9, 17.9]
HDAC-135 non, kpiL: [34.3, 42.3, 42.3]
Time [days]
kpiB
HDAC-123 non, kpiB: [1.1, 1.1, 1.2]
HDAC-135 non, kpiB: [1.2, 1.6, 2.1]
Time [days]
#Leukocytes
HDAC-123 G-CSF, kpiL: [13.8, 13.6, 13.6]
HDAC-135 G-CSF, kpiL: [26.9, 26.3, 26.4]
Time [days]
kpiB
HDAC-123 G-CSF, kpiB: [1.0, 0.9, 0.9]
HDAC-135 G-CSF, kpiB: [1.2, 1.3, 1.5]
Time [days]

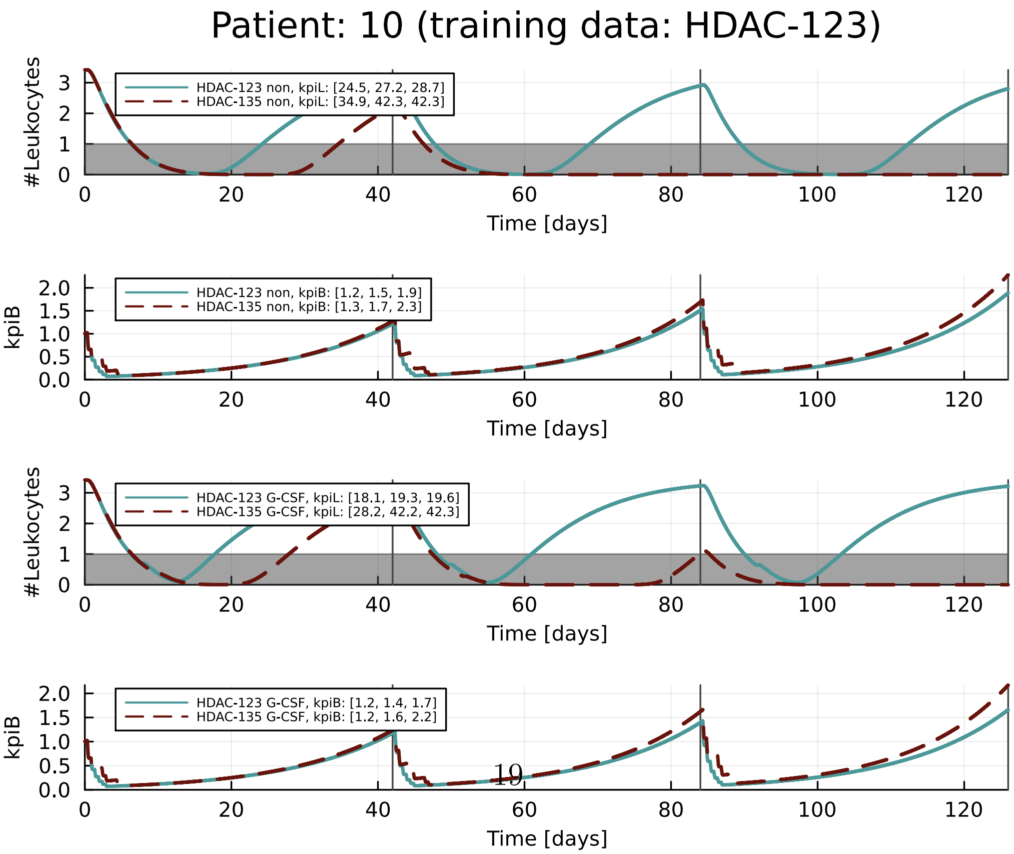
Patient: 10 (training data: HDAC-123)
#Leukocytes
HDAC-123 non, kpiL: [24.5, 27.2, 28.7]
HDAC-135 non, kpiL: [34.9, 42.3, 42.3]
Time [days]
kpiB
HDAC-123 non, kpiB: [1.2, 1.5, 1.9]
HDAC-135 non, kpiB: [1.3, 1.7, 2.3]
Time [days]
#Leukocytes
HDAC-123 G-CSF, kpiL: [18.1, 19.3, 19.6]
HDAC-135 G-CSF, kpiL: [28.2, 42.2, 42.3]
Time [days]
kpiB
HDAC-123 G-CSF, kpiB: [1.2, 1.4, 1.7]
HDAC-135 G-CSF, kpiB: [1.2, 1.6, 2.2]
Time [days]

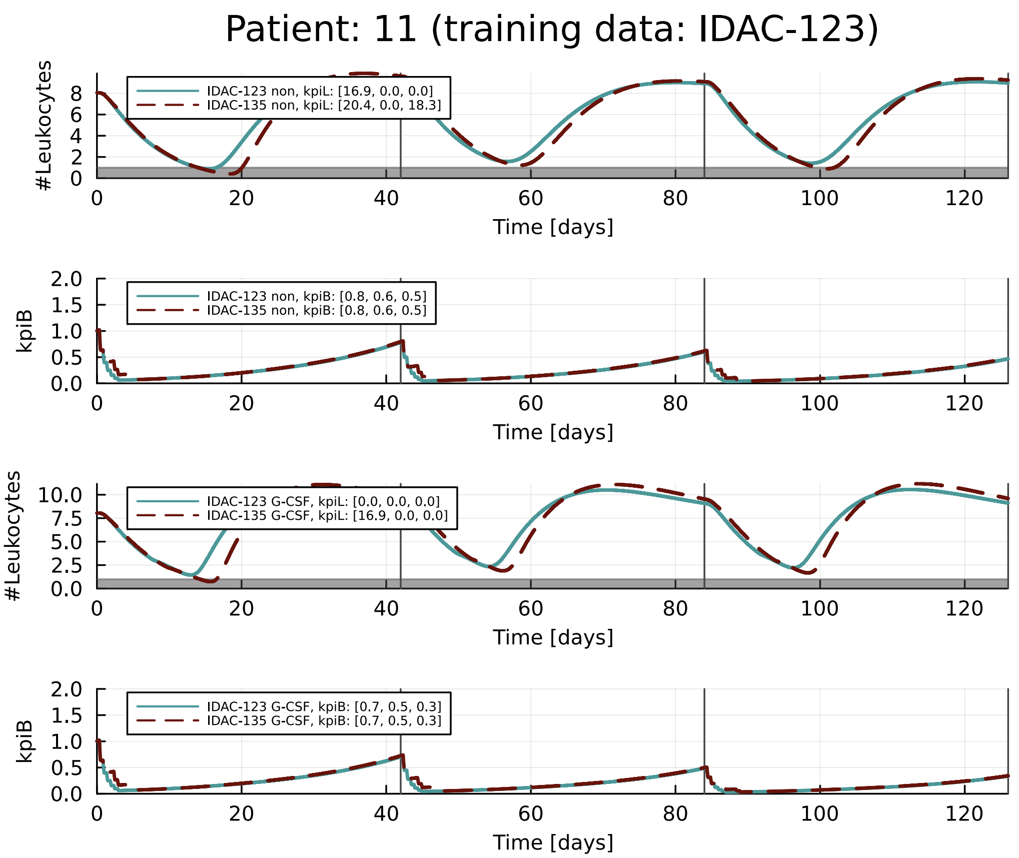
Patient: 11 (training data: IDAC-123)
#Leukocytes
IDAC-123 non, kpiL: [16.9, 0.0, 0.0]
IDAC-135 non, kpiL: [20.4, 0.0, 18.3]
Time [days]
kpiB
IDAC-123 non, kpiB: [0.8, 0.6, 0.5]
IDAC-135 non, kpiB: [0.8, 0.6, 0.5]
Time [days]
#Leukocytes
IDAC-123 G-CSF, kpiL: [0.0, 0.0, 0.0]
IDAC-135 G-CSF, kpiL: [16.9, 0.0, 0.0]
Time [days]
kpiB
IDAC-123 G-CSF, kpiB: [0.7, 0.5, 0.3]
IDAC-135 G-CSF, kpiB: [0.7, 0.5, 0.3]
Time [days]

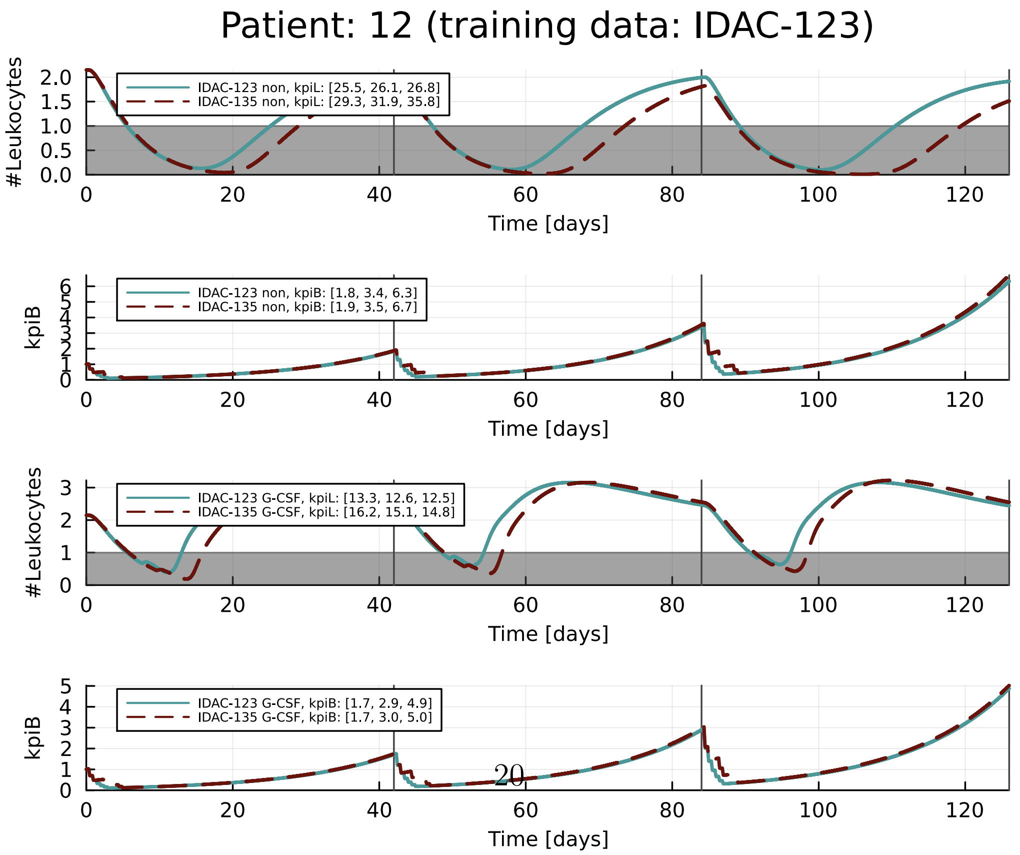
Patient: 12 (training data: IDAC-123)
#Leukocytes
IDAC-123 non, kpiL: [25.5, 26.1, 26.8]
IDAC-135 non, kpiL: [29.3, 31.9, 35.8]
Time [days]
kpiB
IDAC-123 non, kpiB: [1.8, 3.4, 6.3]
IDAC-135 non, kpiB: [1.9, 3.5, 6.7]
Time [days]
#Leukocytes
IDAC-123 G-CSF, kpiL: [13.3, 12.6, 12.5]
IDAC-135 G-CSF, kpiL: [16.2, 15.1, 14.8]
Time [days]
kpiB
IDAC-123 G-CSF, kpiB: [1.7, 2.9, 4.9]
IDAC-135 G-CSF, kpiB: [1.7, 3.0, 5.0]
Time [days]

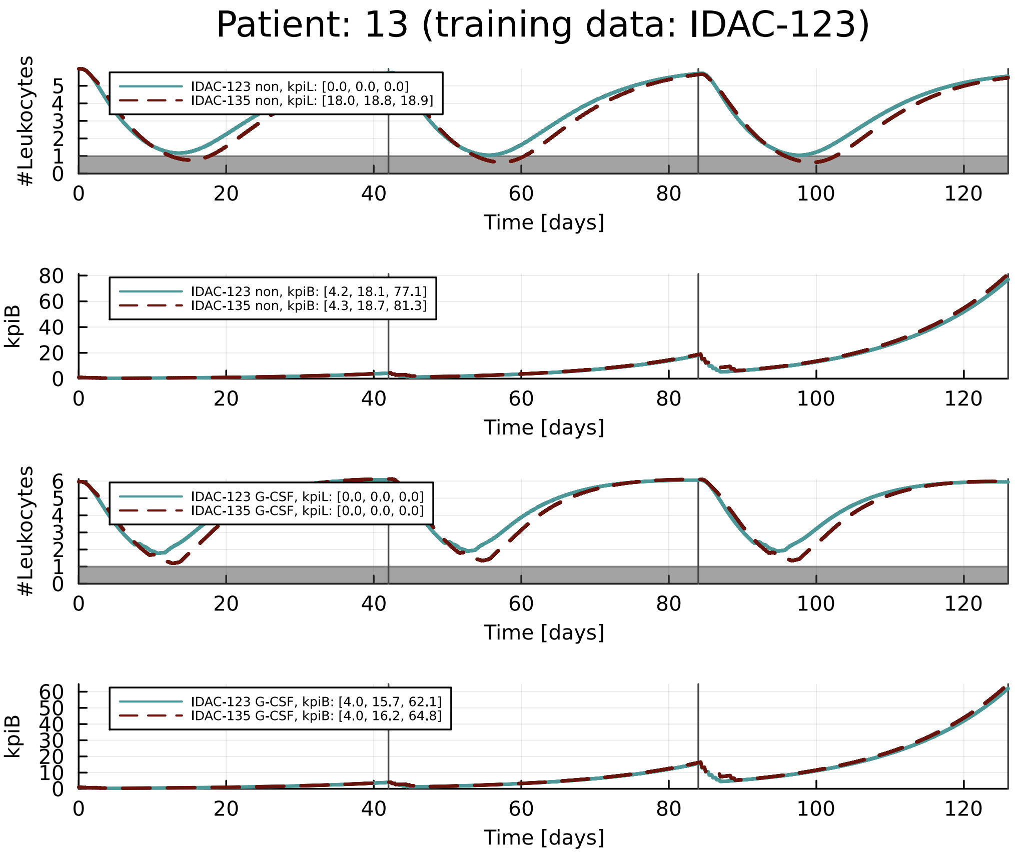
Patient: 13 (training data: IDAC-123)
#Leukocytes
IDAC-123 non, kpiL: [0.0, 0.0, 0.0]
IDAC-135 non, kpiL: [18.0, 18.8, 18.9]
kpiB
IDAC-123 non, kpiB: [4.2, 18.1, 77.1]
IDAC-135 non, kpiB: [4.3, 18.7, 81.3]
IDAC-123 G-CSF, kpiL: [0.0, 0.0, 0.0]
IDAC-135 G-CSF, kpiL: [0.0, 0.0, 0.0]
IDAC-123 G-CSF, kpiB: [4.0, 15.7, 62.1]
IDAC-135 G-CSF, kpiB: [4.0, 16.2, 64.8]
Time [days]

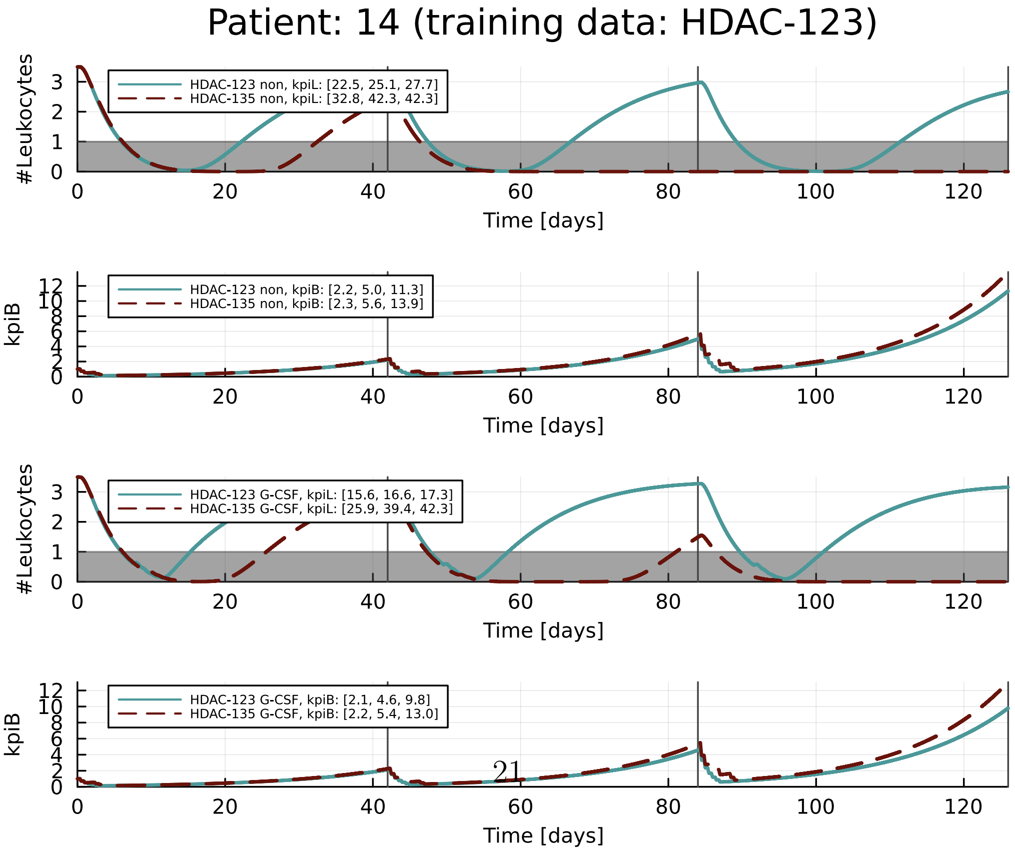
Patient: 14 (training data: HDAC-123)
#Leukocytes
HDAC-123 non, kpiL: [22.5, 25.1, 27.7]
HDAC-135 non, kpiL: [32.8, 42.3, 42.3]
kpiB
HDAC-123 non, kpiB: [2.2, 5.0, 11.3]
HDAC-135 non, kpiB: [2.3, 5.6, 13.9]
HDAC-123 G-CSF, kpiL: [15.6, 16.6, 17.3]
HDAC-135 G-CSF, kpiL: [25.9, 39.4, 42.3]
HDAC-123 G-CSF, kpiB: [2.1, 4.6, 9.8]
HDAC-135 G-CSF, kpiB: [2.2, 5.4, 13.0]
Time [days]

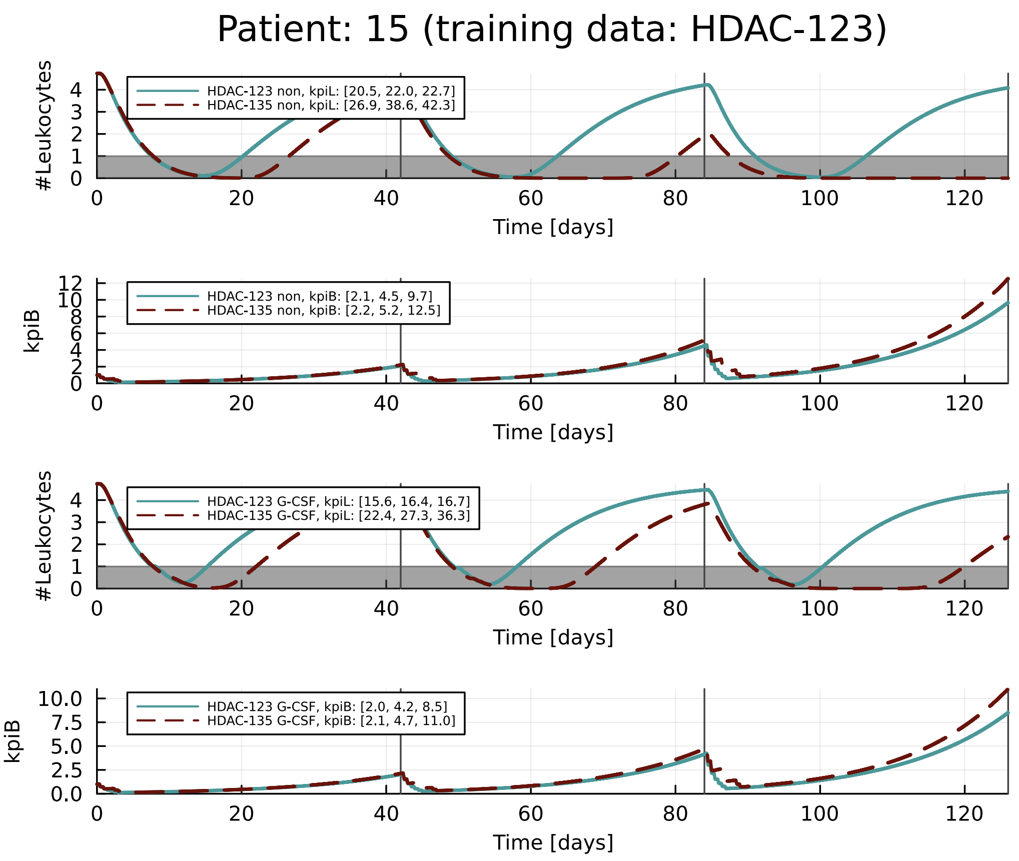
Patient: 15 (training data: HDAC-123)
HDAC-123 non, kpiL: [20.5, 22.0, 22.7]
HDAC-135 non, kpiL: [26.9, 38.6, 42.3]
HDAC-123 non, kpiB: [2.1, 4.5, 9.7]
HDAC-135 non, kpiB: [2.2, 5.2, 12.5]
HDAC-123 G-CSF, kpiL: [15.6, 16.4, 16.7]
HDAC-135 G-CSF, kpiL: [22.4, 27.3, 36.3]
HDAC-123 G-CSF, kpiB: [2.0, 4.2, 8.5]
HDAC-135 G-CSF, kpiB: [2.1, 4.7, 11.0]
#Leukocytes
kpiB
Time [days]

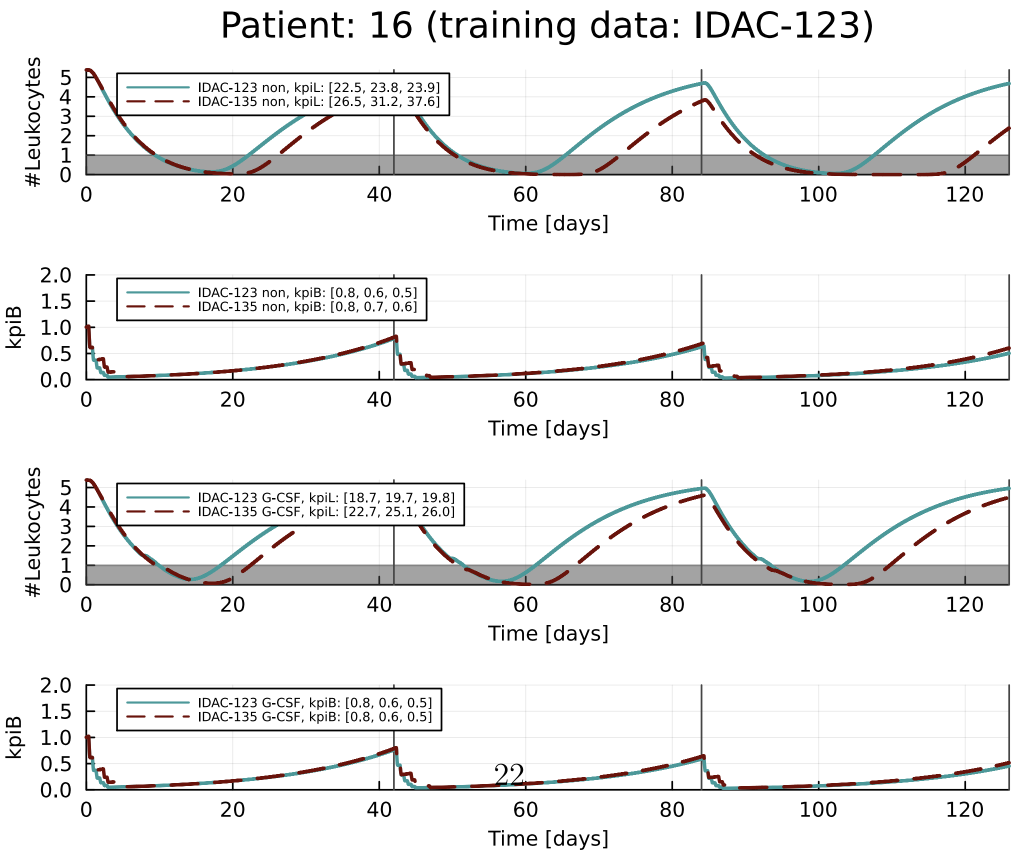
Patient: 16 (training data: IDAC-123)
IDAC-123 non, kpiL: [22.5, 23.8, 23.9]
IDAC-135 non, kpiL: [26.5, 31.2, 37.6]
IDAC-123 non, kpiB: [0.8, 0.6, 0.5]
IDAC-135 non, kpiB: [0.8, 0.7, 0.6]
IDAC-123 G-CSF, kpiL: [18.7, 19.7, 19.8]
IDAC-135 G-CSF, kpiL: [22.7, 25.1, 26.0]
IDAC-123 G-CSF, kpiB: [0.8, 0.6, 0.5]
IDAC-135 G-CSF, kpiB: [0.8, 0.6, 0.5]
#Leukocytes
kpiB
Time [days]

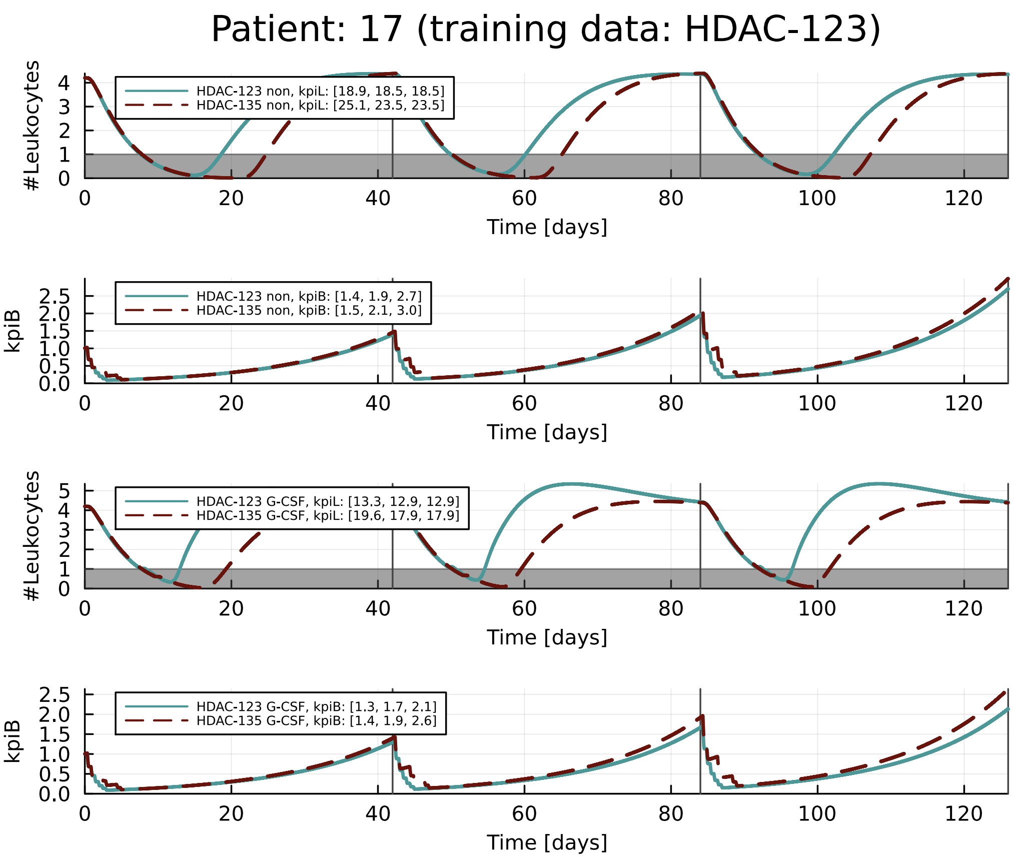

Patient: 17 (training data: HDAC-123)
HDAC-123 non, kpiL: [18.9, 18.5, 18.5]
HDAC-135 non, kpiL: [25.1, 23.5, 23.5]
#Leukocytes
Time [days]
HDAC-123 non, kpiB: [1.4, 1.9, 2.7]
HDAC-135 non, kpiB: [1.5, 2.1, 3.0]
kpiB
Time [days]
HDAC-123 G-CSF, kpiL: [13.3, 12.9, 12.9]
HDAC-135 G-CSF, kpiL: [19.6, 17.9, 17.9]
#Leukocytes
Time [days]
HDAC-123 G-CSF, kpiB: [1.3, 1.7, 2.1]
HDAC-135 G-CSF, kpiB: [1.4, 1.9, 2.6]
kpiB
Time [days]

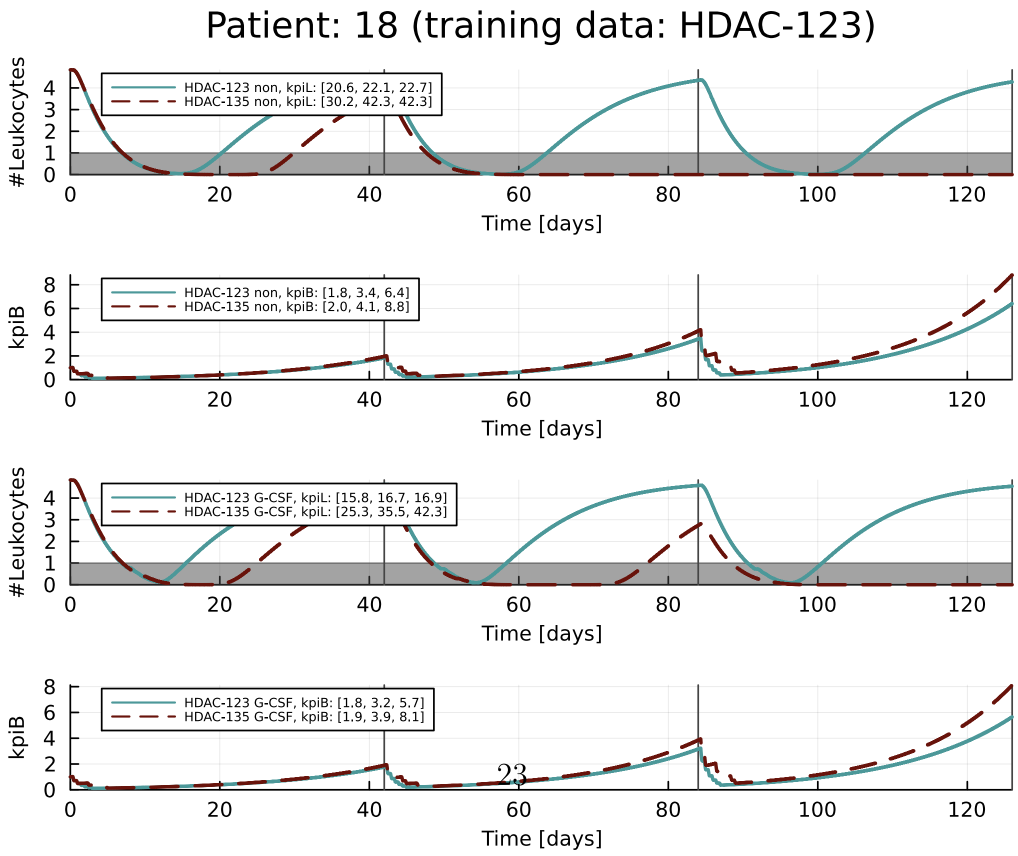

Patient: 18 (training data: HDAC-123)
HDAC-123 non, kpiL: [20.6, 22.1, 22.7]
HDAC-135 non, kpiL: [30.2, 42.3, 42.3]
#Leukocytes
Time [days]
HDAC-123 non, kpiB: [1.8, 3.4, 6.4]
HDAC-135 non, kpiB: [2.0, 4.1, 8.8]
kpiB
Time [days]
HDAC-123 G-CSF, kpiL: [15.8, 16.7, 16.9]
HDAC-135 G-CSF, kpiL: [25.3, 35.5, 42.3]
#Leukocytes
Time [days]
HDAC-123 G-CSF, kpiB: [1.8, 3.2, 5.7]
HDAC-135 G-CSF, kpiB: [1.9, 3.9, 8.1]
kpiB
Time [days]

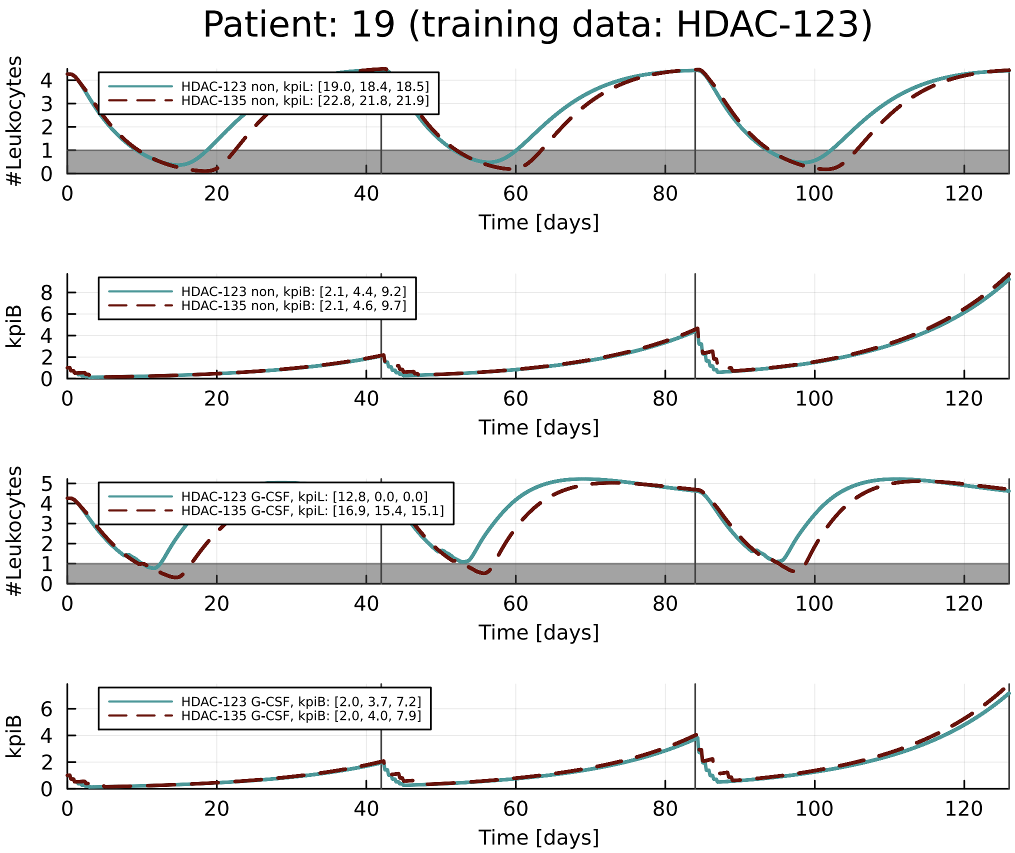
Patient: 19 (training data: HDAC-123)
HDAC-123 non, kpiL: [19.0, 18.4, 18.5]
HDAC-135 non, kpiL: [22.8, 21.8, 21.9]
HDAC-123 non, kpiB: [2.1, 4.4, 9.2]
HDAC-135 non, kpiB: [2.1, 4.6, 9.7]
HDAC-123 G-CSF, kpiL: [12.8, 0.0, 0.0]
HDAC-135 G-CSF, kpiL: [16.9, 15.4, 15.1]
HDAC-123 G-CSF, kpiB: [2.0, 3.7, 7.2]
HDAC-135 G-CSF, kpiB: [2.0, 4.0, 7.9]
#Leukocytes
kpiB
Time [days]

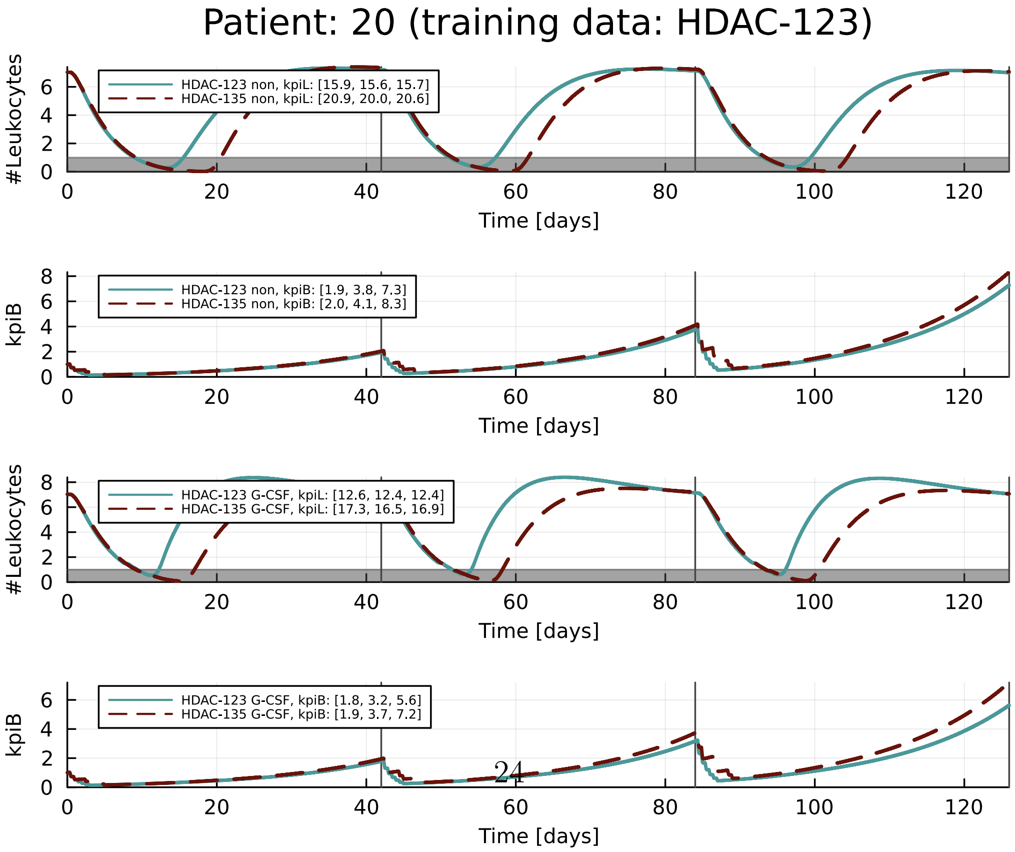
Patient: 20 (training data: HDAC-123)
HDAC-123 non, kpiL: [15.9, 15.6, 15.7]
HDAC-135 non, kpiL: [20.9, 20.0, 20.6]
HDAC-123 non, kpiB: [1.9, 3.8, 7.3]
HDAC-135 non, kpiB: [2.0, 4.1, 8.3]
HDAC-123 G-CSF, kpiL: [12.6, 12.4, 12.4]
HDAC-135 G-CSF, kpiL: [17.3, 16.5, 16.9]
HDAC-123 G-CSF, kpiB: [1.8, 3.2, 5.6]
HDAC-135 G-CSF, kpiB: [1.9, 3.7, 7.2]
#Leukocytes
kpiB
Time [days]

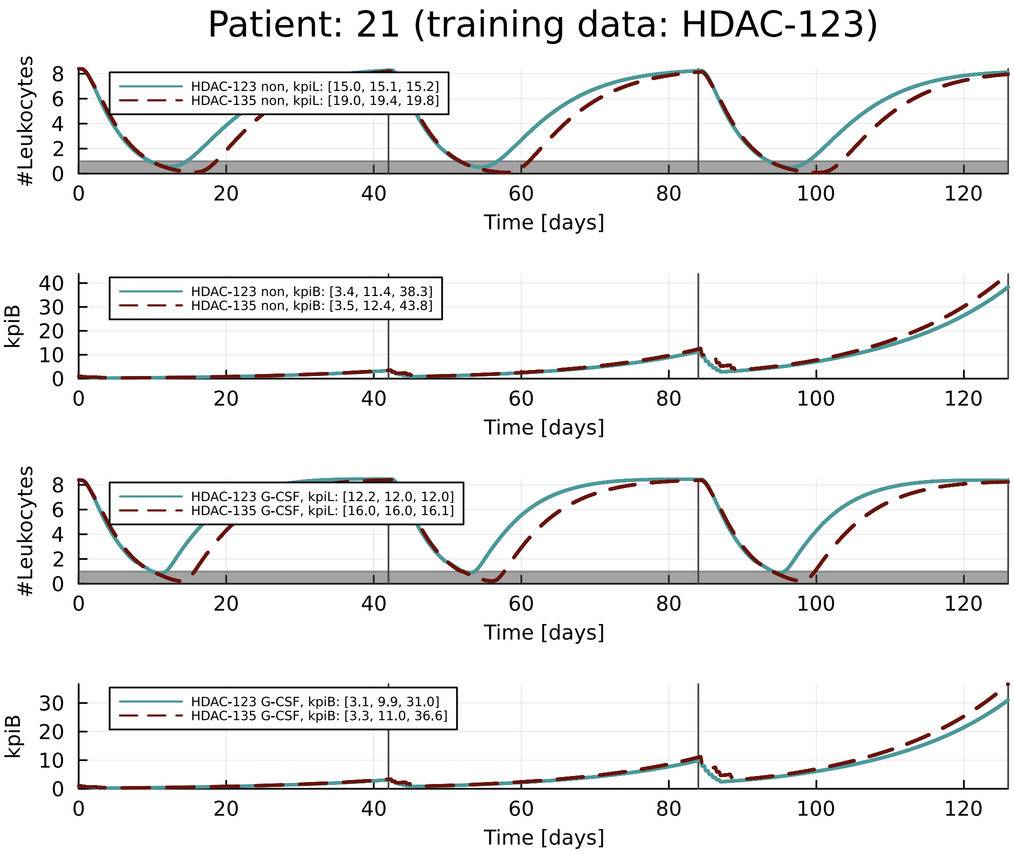
Patient: 21 (training data: HDAC-123)
HDAC-123 non, kpiL: [15.0, 15.1, 15.2]
HDAC-135 non, kpiL: [19.0, 19.4, 19.8]
HDAC-123 non, kpiB: [3.4, 11.4, 38.3]
HDAC-135 non, kpiB: [3.5, 12.4, 43.8]
HDAC-123 G-CSF, kpiL: [12.2, 12.0, 12.0]
HDAC-135 G-CSF, kpiL: [16.0, 16.0, 16.1]
HDAC-123 G-CSF, kpiB: [3.1, 9.9, 31.0]
HDAC-135 G-CSF, kpiB: [3.3, 11.0, 36.6]
#Leukocytes
kpiB
Time [days]

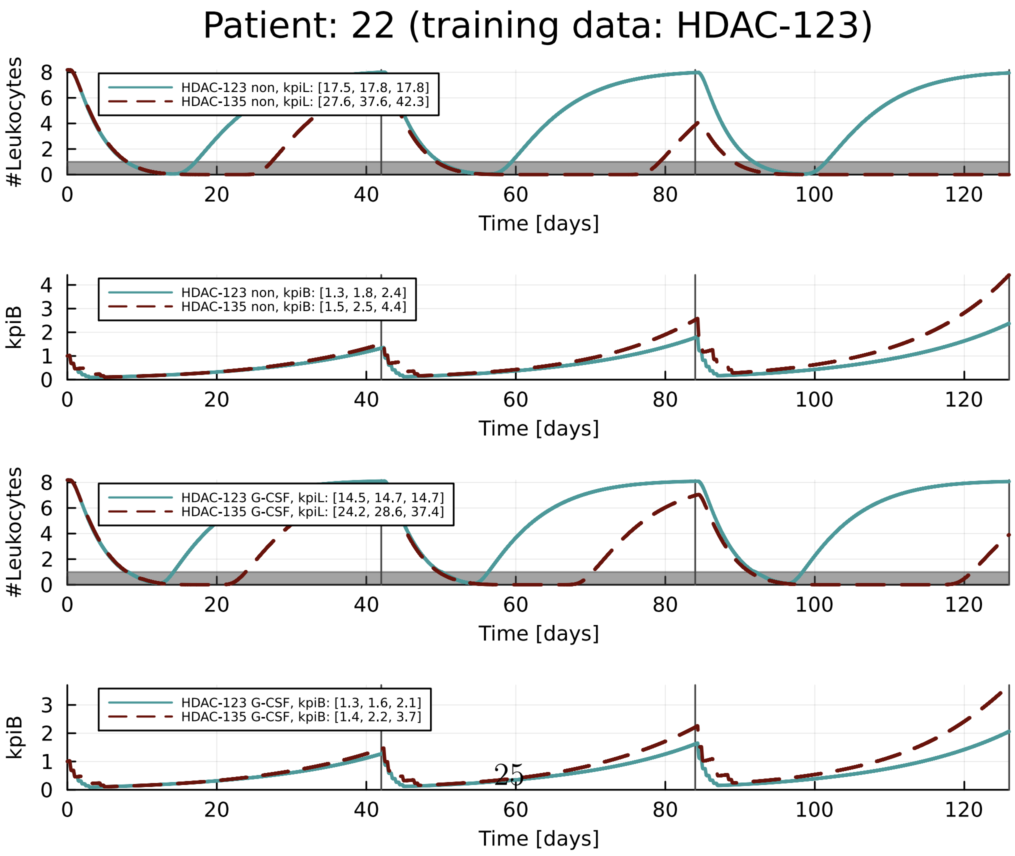
Patient: 22 (training data: HDAC-123)
HDAC-123 non, kpiL: [17.5, 17.8, 17.8]
HDAC-135 non, kpiL: [27.6, 37.6, 42.3]
HDAC-123 non, kpiB: [1.3, 1.8, 2.4]
HDAC-135 non, kpiB: [1.5, 2.5, 4.4]
HDAC-123 G-CSF, kpiL: [14.5, 14.7, 14.7]
HDAC-135 G-CSF, kpiL: [24.2, 28.6, 37.4]
HDAC-123 G-CSF, kpiB: [1.3, 1.6, 2.1]
HDAC-135 G-CSF, kpiB: [1.4, 2.2, 3.7]
#Leukocytes
kpiB
Time [days]

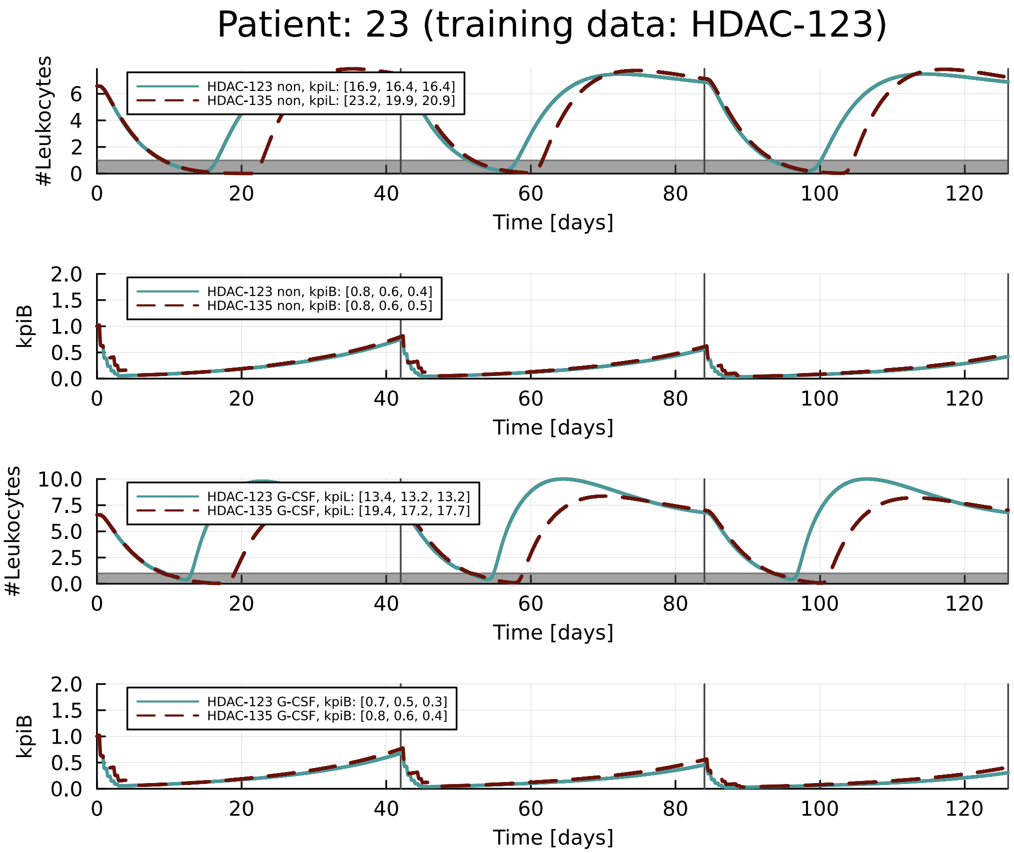
Patient: 23 (training data: HDAC-123)
#Leukocytes
HDAC-123 non, kpiL: [16.9, 16.4, 16.4]
HDAC-135 non, kpiL: [23.2, 19.9, 20.9]
Time [days]
kpiB
HDAC-123 non, kpiB: [0.8, 0.6, 0.4]
HDAC-135 non, kpiB: [0.8, 0.6, 0.5]
Time [days]
#Leukocytes
HDAC-123 G-CSF, kpiL: [13.4, 13.2, 13.2]
HDAC-135 G-CSF, kpiL: [19.4, 17.2, 17.7]
Time [days]
kpiB
HDAC-123 G-CSF, kpiB: [0.7, 0.5, 0.3]
HDAC-135 G-CSF, kpiB: [0.8, 0.6, 0.4]
Time [days]

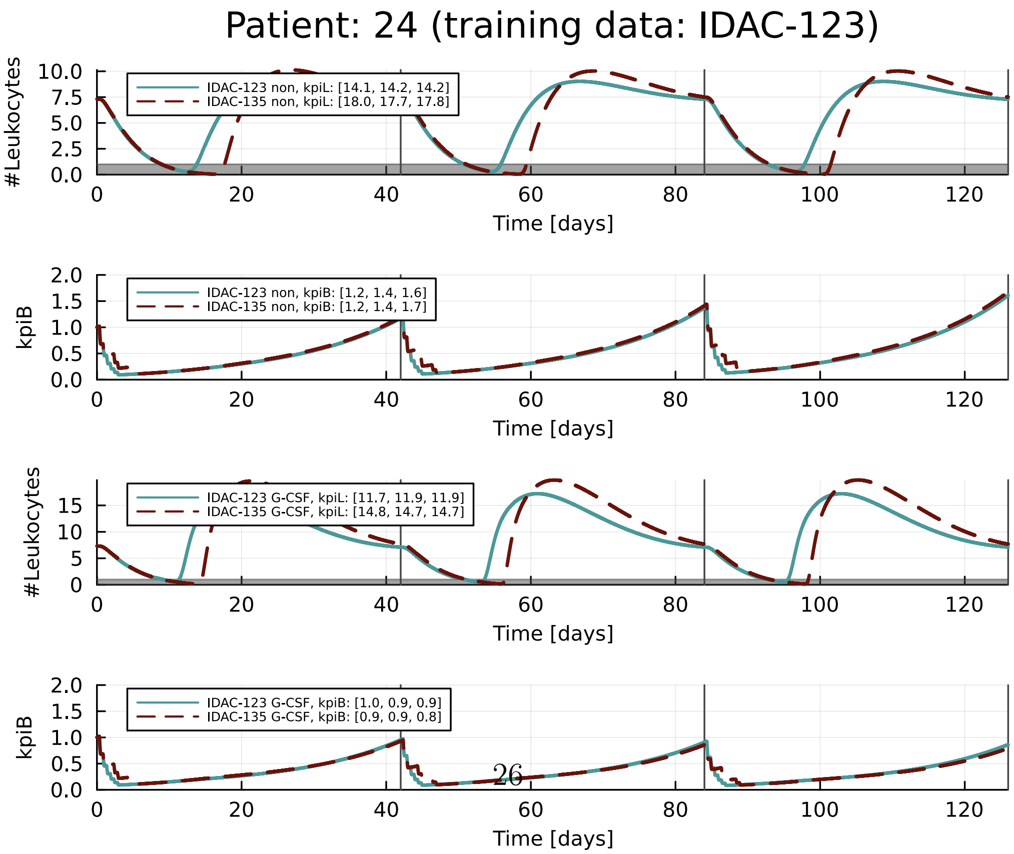
Patient: 24 (training data: IDAC-123)
#Leukocytes
IDAC-123 non, kpiL: [14.1, 14.2, 14.2]
IDAC-135 non, kpiL: [18.0, 17.7, 17.8]
Time [days]
kpiB
IDAC-123 non, kpiB: [1.2, 1.4, 1.6]
IDAC-135 non, kpiB: [1.2, 1.4, 1.7]
Time [days]
#Leukocytes
IDAC-123 G-CSF, kpiL: [11.7, 11.9, 11.9]
IDAC-135 G-CSF, kpiL: [14.8, 14.7, 14.7]
Time [days]
kpiB
IDAC-123 G-CSF, kpiB: [1.0, 0.9, 0.9]
IDAC-135 G-CSF, kpiB: [0.9, 0.9, 0.8]
Time [days]

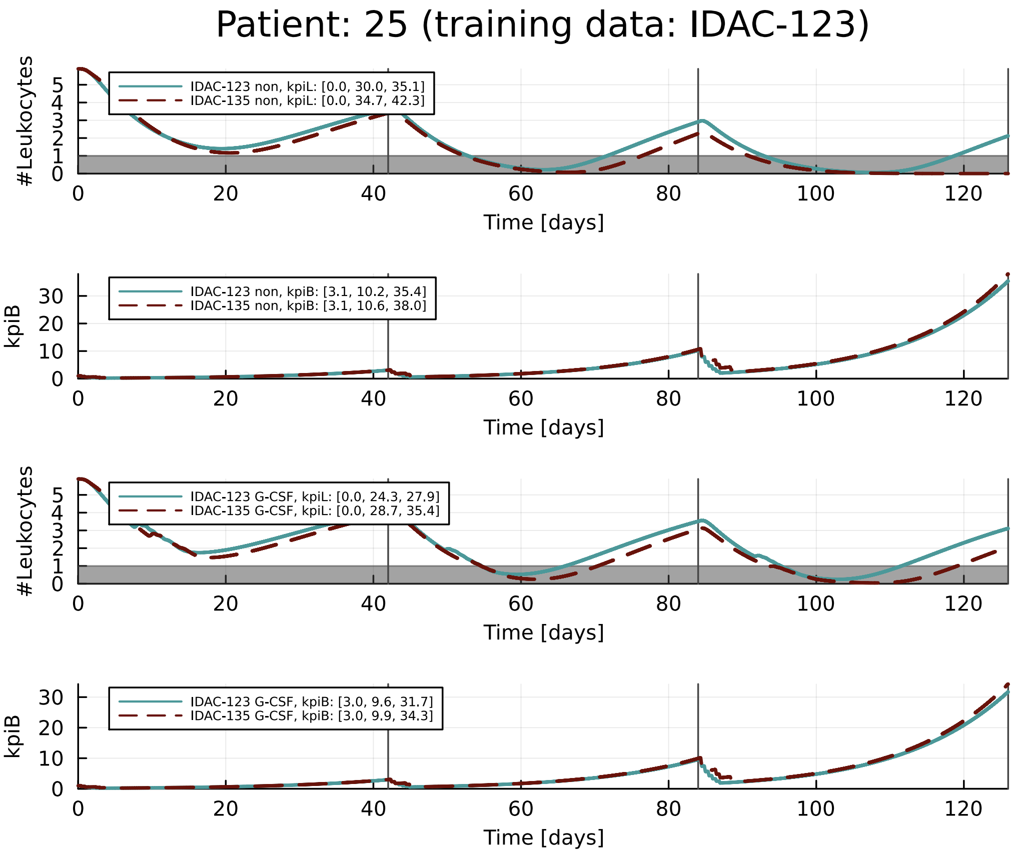
Patient: 25 (training data: IDAC-123)
IDAC-123 non, kpiL: [0.0, 30.0, 35.1]
IDAC-135 non, kpiL: [0.0, 34.7, 42.3]
#Leukocytes
Time [days]
IDAC-123 non, kpiB: [3.1, 10.2, 35.4]
IDAC-135 non, kpiB: [3.1, 10.6, 38.0]
kpiB
Time [days]
IDAC-123 G-CSF, kpiL: [0.0, 24.3, 27.9]
IDAC-135 G-CSF, kpiL: [0.0, 28.7, 35.4]
#Leukocytes
Time [days]
IDAC-123 G-CSF, kpiB: [3.0, 9.6, 31.7]
IDAC-135 G-CSF, kpiB: [3.0, 9.9, 34.3]
kpiB
Time [days]

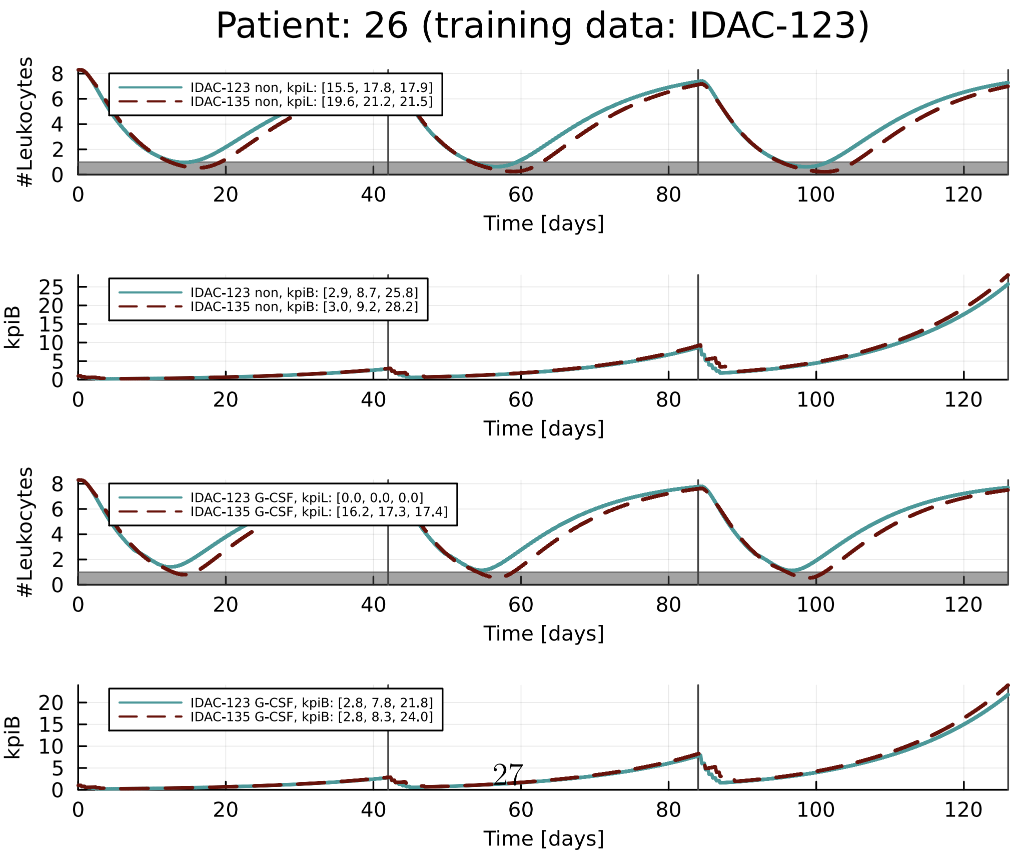
Patient: 26 (training data: IDAC-123)
IDAC-123 non, kpiL: [15.5, 17.8, 17.9]
IDAC-135 non, kpiL: [19.6, 21.2, 21.5]
#Leukocytes
Time [days]
IDAC-123 non, kpiB: [2.9, 8.7, 25.8]
IDAC-135 non, kpiB: [3.0, 9.2, 28.2]
kpiB
Time [days]
IDAC-123 G-CSF, kpiL: [0.0, 0.0, 0.0]
IDAC-135 G-CSF, kpiL: [16.2, 17.3, 17.4]
#Leukocytes
Time [days]
IDAC-123 G-CSF, kpiB: [2.8, 7.8, 21.8]
IDAC-135 G-CSF, kpiB: [2.8, 8.3, 24.0]
kpiB
Time [days]

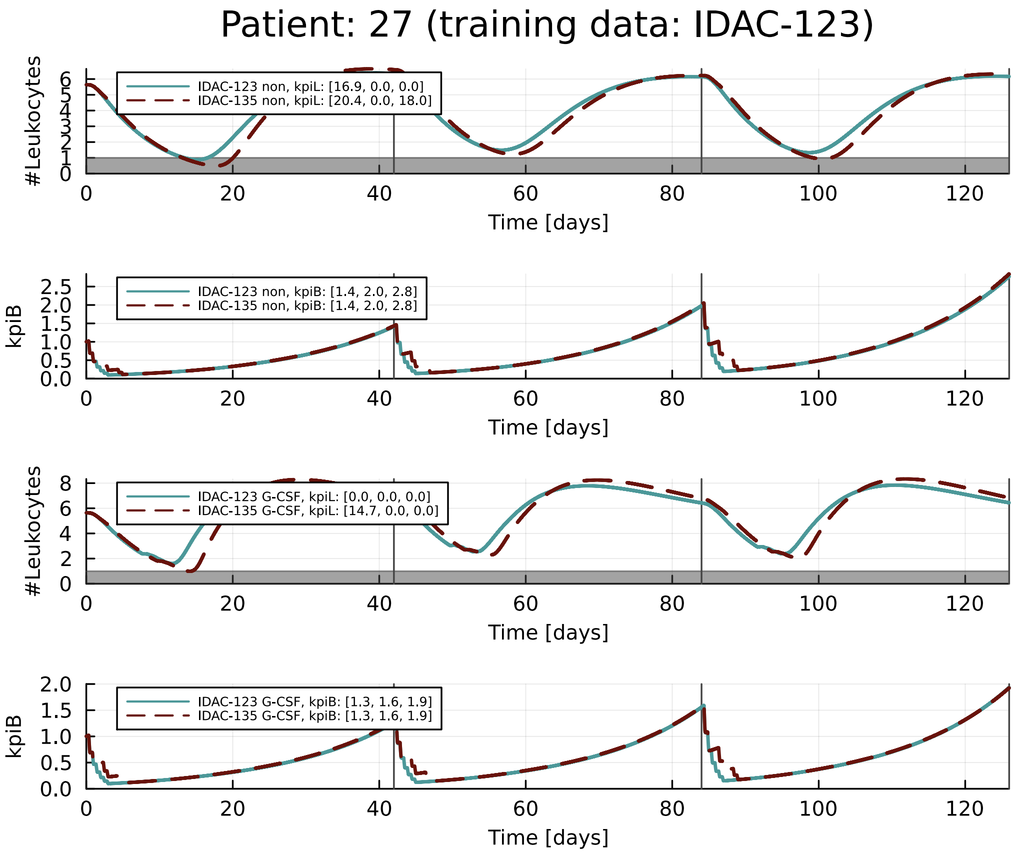
Patient: 27 (training data: IDAC-123)
IDAC-123 non, kpiL: [16.9, 0.0, 0.0]
IDAC-135 non, kpiL: [20.4, 0.0, 18.0]
IDAC-123 non, kpiB: [1.4, 2.0, 2.8]
IDAC-135 non, kpiB: [1.4, 2.0, 2.8]
IDAC-123 G-CSF, kpiL: [0.0, 0.0, 0.0]
IDAC-135 G-CSF, kpiL: [14.7, 0.0, 0.0]
IDAC-123 G-CSF, kpiB: [1.3, 1.6, 1.9]
IDAC-135 G-CSF, kpiB: [1.3, 1.6, 1.9]
#Leukocytes
kpiB
Time [days]

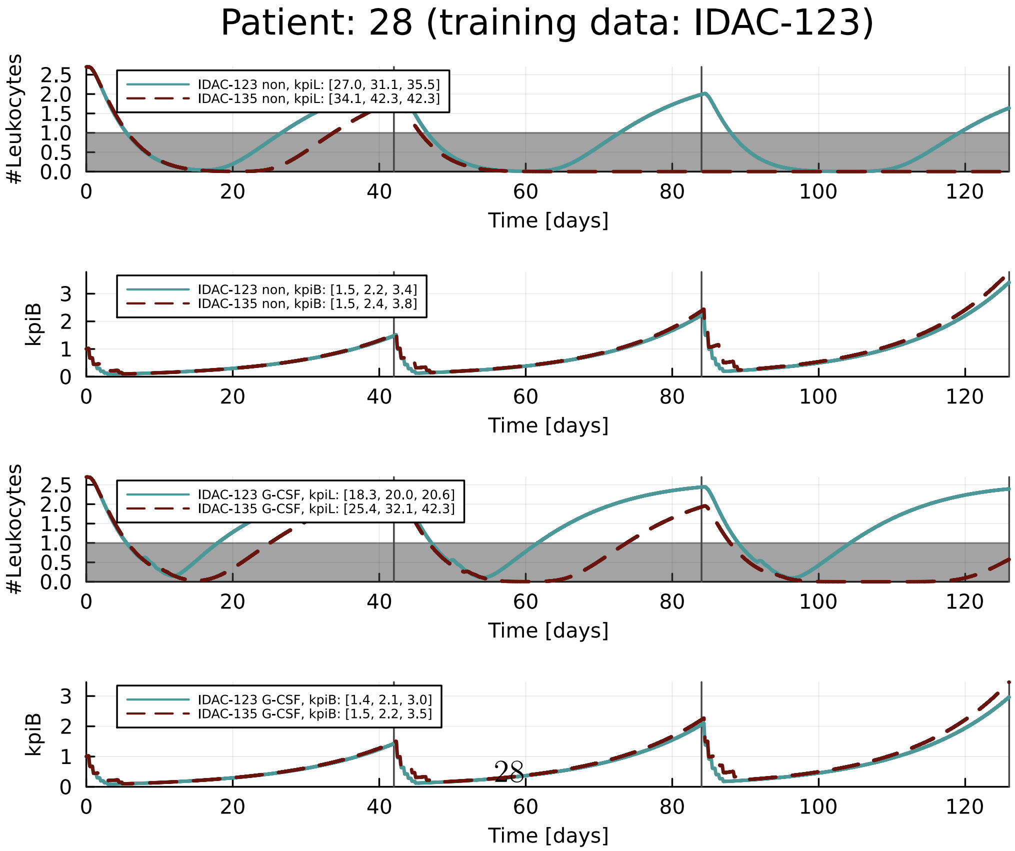
Patient: 28 (training data: IDAC-123)
IDAC-123 non, kpiL: [27.0, 31.1, 35.5]
IDAC-135 non, kpiL: [34.1, 42.3, 42.3]
IDAC-123 non, kpiB: [1.5, 2.2, 3.4]
IDAC-135 non, kpiB: [1.5, 2.4, 3.8]
IDAC-123 G-CSF, kpiL: [18.3, 20.0, 20.6]
IDAC-135 G-CSF, kpiL: [25.4, 32.1, 42.3]
IDAC-123 G-CSF, kpiB: [1.4, 2.1, 3.0]
IDAC-135 G-CSF, kpiB: [1.5, 2.2, 3.5]
#Leukocytes
kpiB
Time [days]

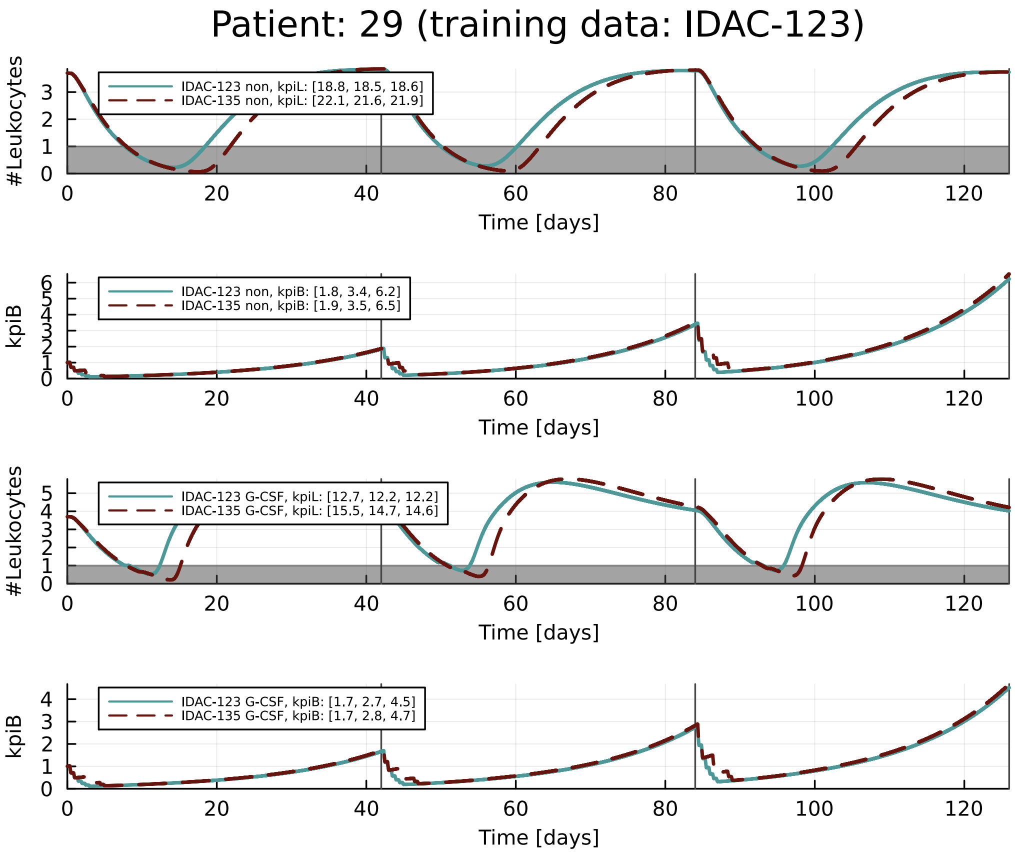
Patient: 29 (training data: IDAC-123)
IDAC-123 non, kpiL: [18.8, 18.5, 18.6]
IDAC-135 non, kpiL: [22.1, 21.6, 21.9]
IDAC-123 non, kpiB: [1.8, 3.4, 6.2]
IDAC-135 non, kpiB: [1.9, 3.5, 6.5]
IDAC-123 G-CSF, kpiL: [12.7, 12.2, 12.2]
IDAC-135 G-CSF, kpiL: [15.5, 14.7, 14.6]
IDAC-123 G-CSF, kpiB: [1.7, 2.7, 4.5]
IDAC-135 G-CSF, kpiB: [1.7, 2.8, 4.7]
#Leukocytes
kpiB
Time [days]

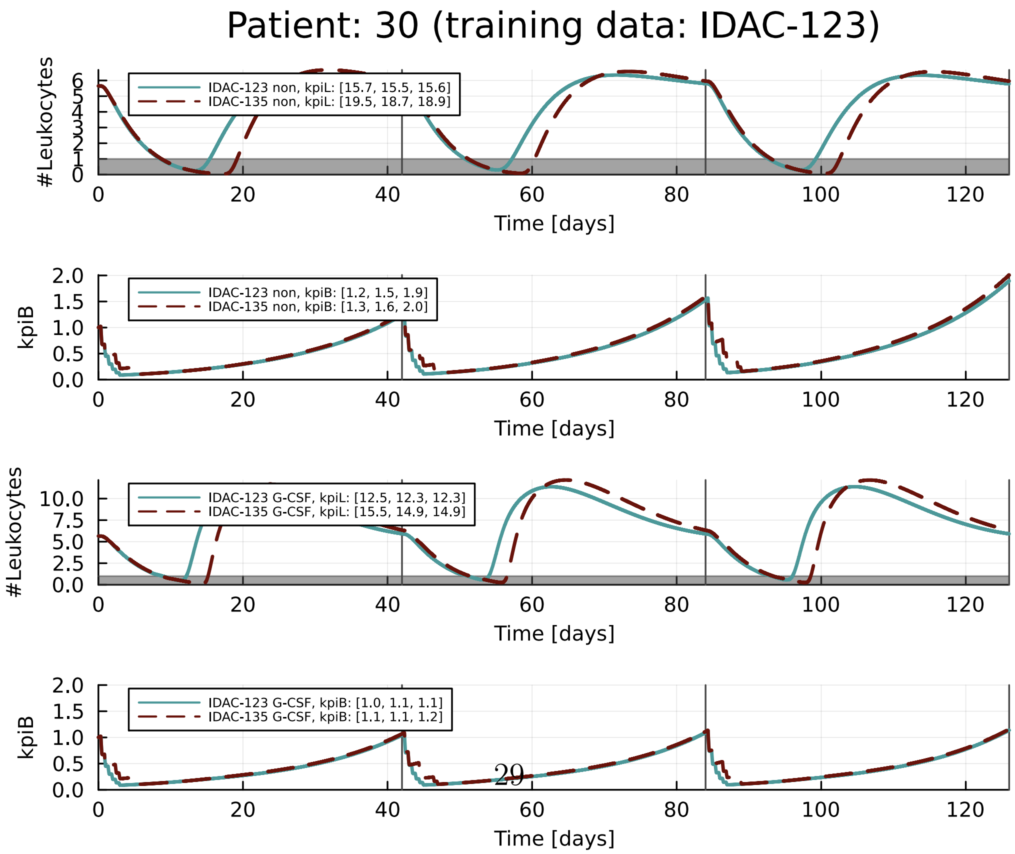
Patient: 30 (training data: IDAC-123)
IDAC-123 non, kpiL: [15.7, 15.5, 15.6]
IDAC-135 non, kpiL: [19.5, 18.7, 18.9]
IDAC-123 non, kpiB: [1.2, 1.5, 1.9]
IDAC-135 non, kpiB: [1.3, 1.6, 2.0]
IDAC-123 G-CSF, kpiL: [12.5, 12.3, 12.3]
IDAC-135 G-CSF, kpiL: [15.5, 14.9, 14.9]
IDAC-123 G-CSF, kpiB: [1.0, 1.1, 1.1]
IDAC-135 G-CSF, kpiB: [1.1, 1.1, 1.2]
#Leukocytes
kpiB
Time [days]

Patient: 31 (training data: IDAC-123)

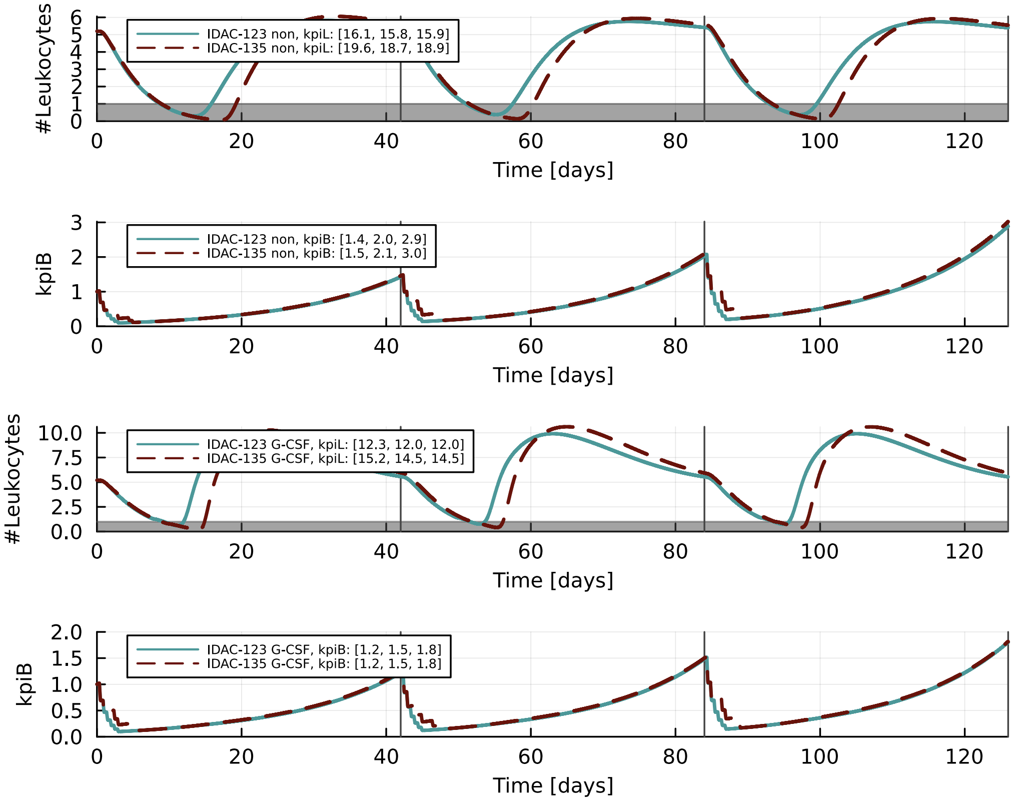

Patient: 32 (training data: IDAC-123)

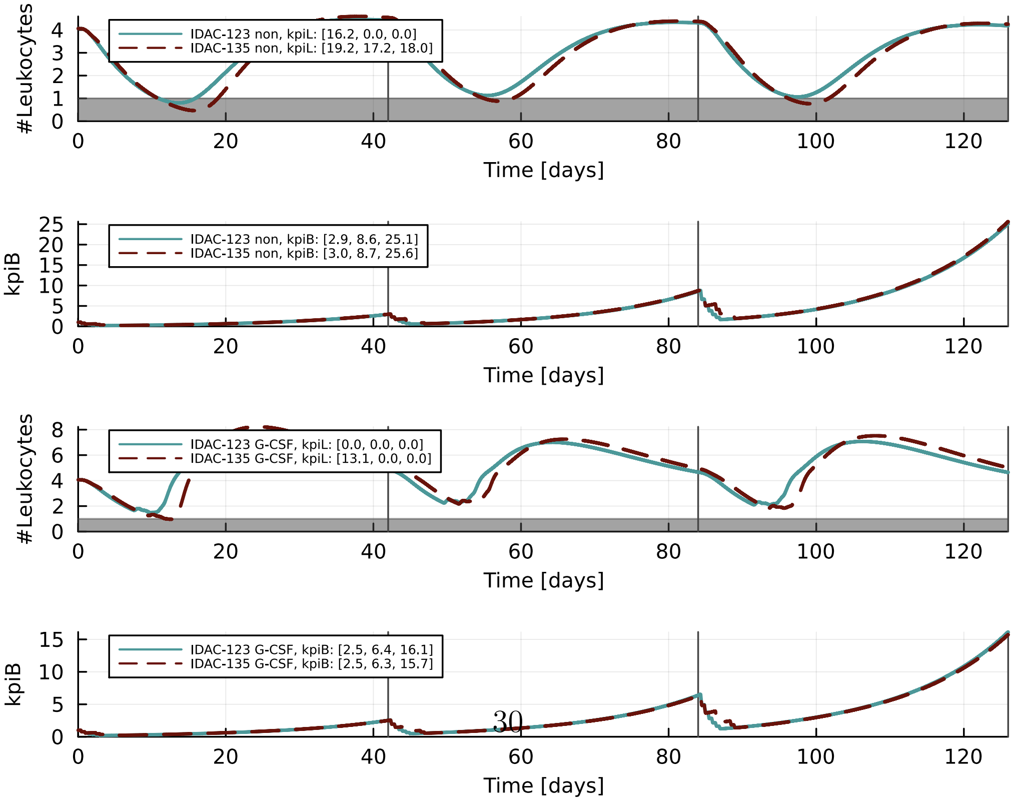

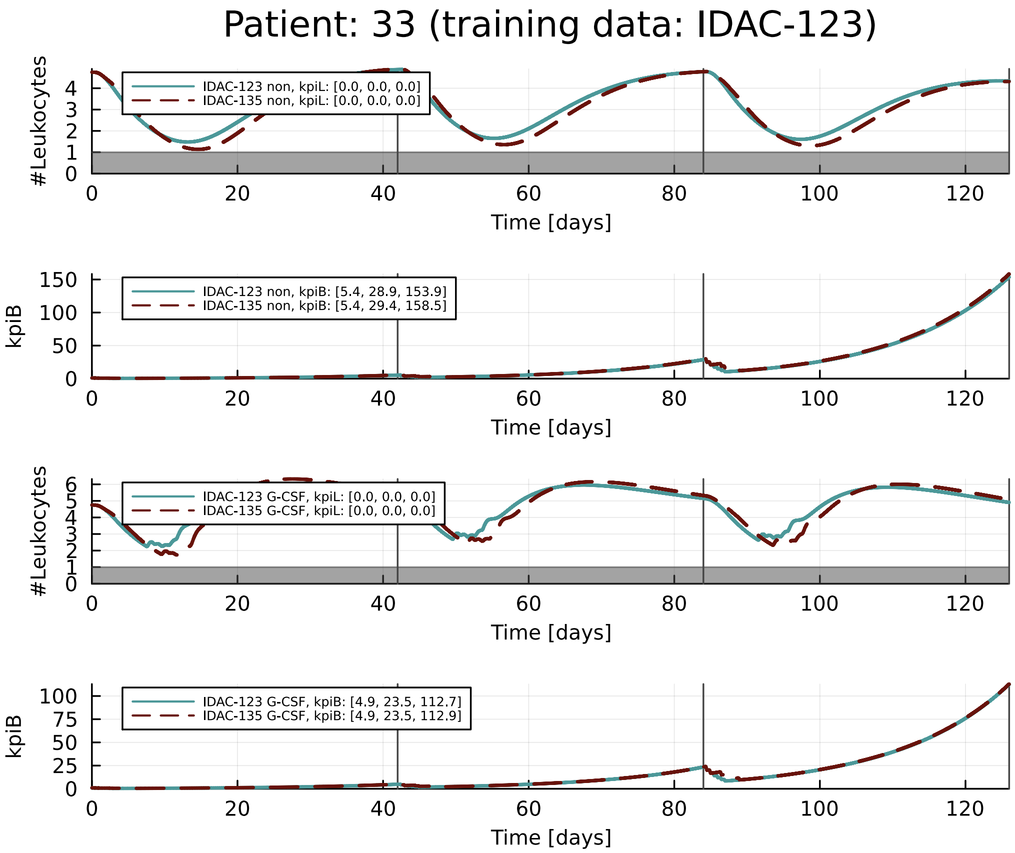
Patient: 33 (training data: IDAC-123)
#Leukocytes
IDAC-123 non, kpiL: [0.0, 0.0, 0.0]
IDAC-135 non, kpiL: [0.0, 0.0, 0.0]
Time [days]
kpiB
IDAC-123 non, kpiB: [5.4, 28.9, 153.9]
IDAC-135 non, kpiB: [5.4, 29.4, 158.5]
Time [days]
#Leukocytes
IDAC-123 G-CSF, kpiL: [0.0, 0.0, 0.0]
IDAC-135 G-CSF, kpiL: [0.0, 0.0, 0.0]
Time [days]
kpiB
IDAC-123 G-CSF, kpiB: [4.9, 23.5, 112.7]
IDAC-135 G-CSF, kpiB: [4.9, 23.5, 112.9]
Time [days]

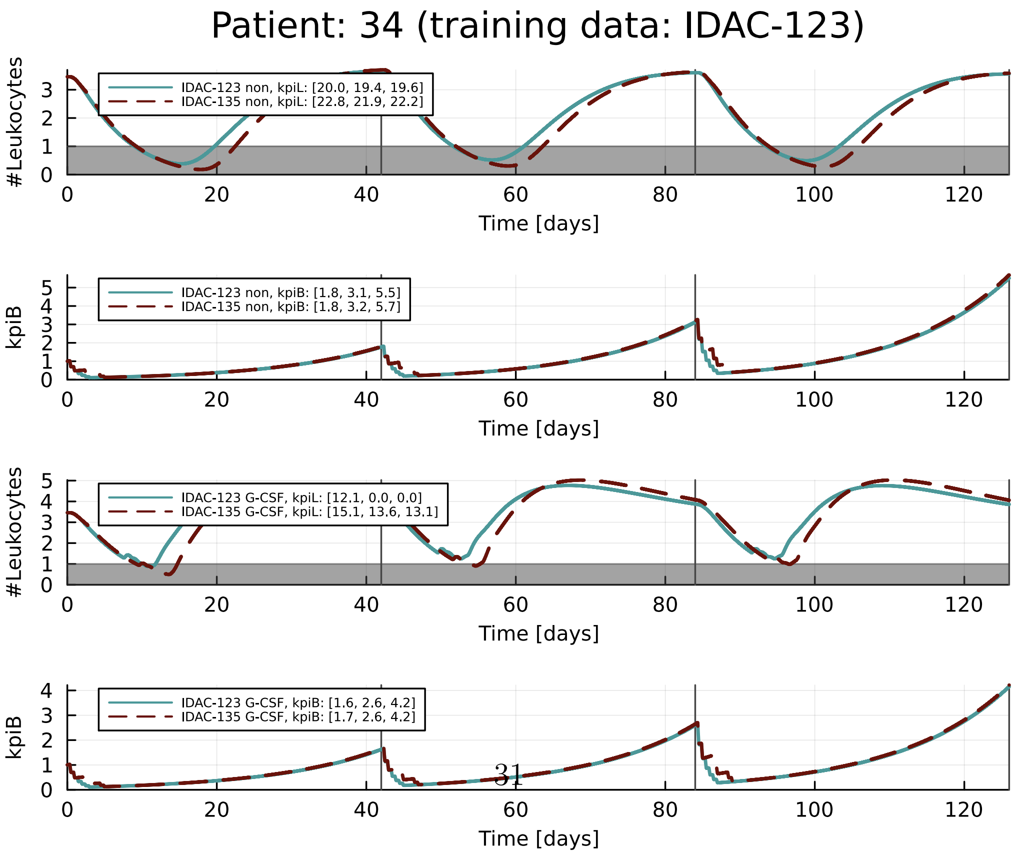
Patient: 34 (training data: IDAC-123)
#Leukocytes
IDAC-123 non, kpiL: [20.0, 19.4, 19.6]
IDAC-135 non, kpiL: [22.8, 21.9, 22.2]
Time [days]
kpiB
IDAC-123 non, kpiB: [1.8, 3.1, 5.5]
IDAC-135 non, kpiB: [1.8, 3.2, 5.7]
Time [days]
#Leukocytes
IDAC-123 G-CSF, kpiL: [12.1, 0.0, 0.0]
IDAC-135 G-CSF, kpiL: [15.1, 13.6, 13.1]
Time [days]
kpiB
IDAC-123 G-CSF, kpiB: [1.6, 2.6, 4.2]
IDAC-135 G-CSF, kpiB: [1.7, 2.6, 4.2]
Time [days]

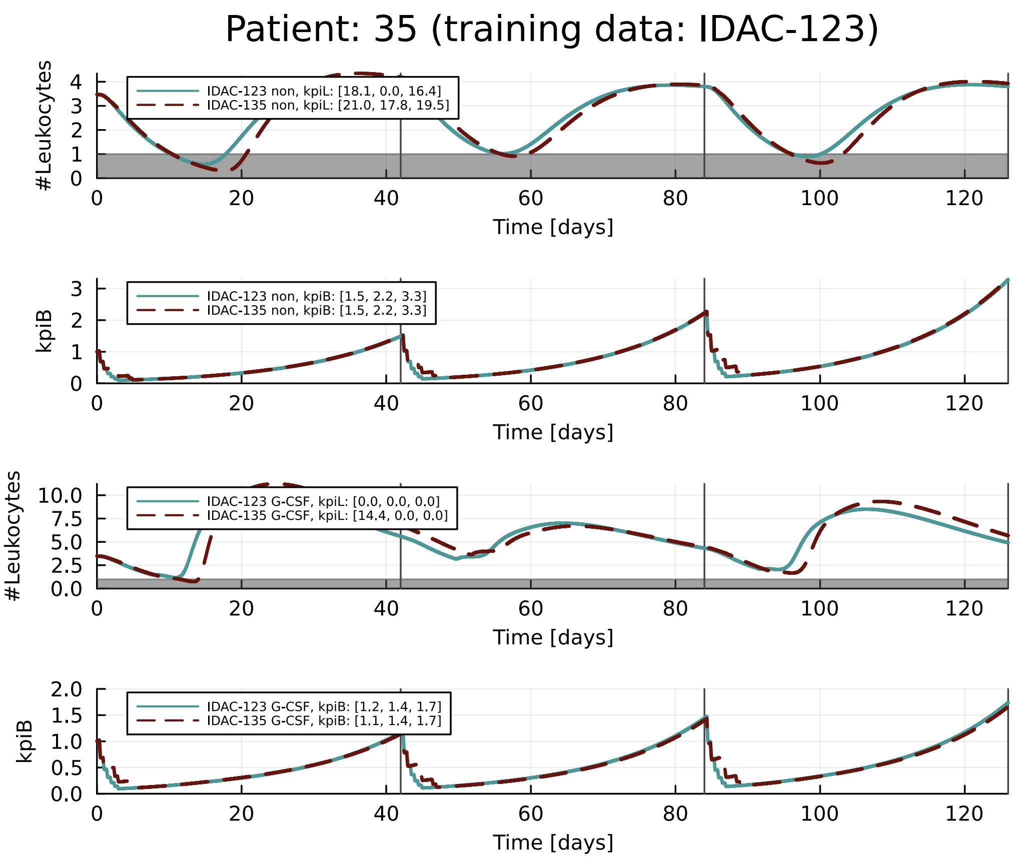
Patient: 35 (training data: IDAC-123)
IDAC-123 non, kpiL: [18.1, 0.0, 16.4]
IDAC-135 non, kpiL: [21.0, 17.8, 19.5]
IDAC-123 non, kpiB: [1.5, 2.2, 3.3]
IDAC-135 non, kpiB: [1.5, 2.2, 3.3]
IDAC-123 G-CSF, kpiL: [0.0, 0.0, 0.0]
IDAC-135 G-CSF, kpiL: [14.4, 0.0, 0.0]
IDAC-123 G-CSF, kpiB: [1.2, 1.4, 1.7]
IDAC-135 G-CSF, kpiB: [1.1, 1.4, 1.7]
#Leukocytes
kpiB
Time [days]

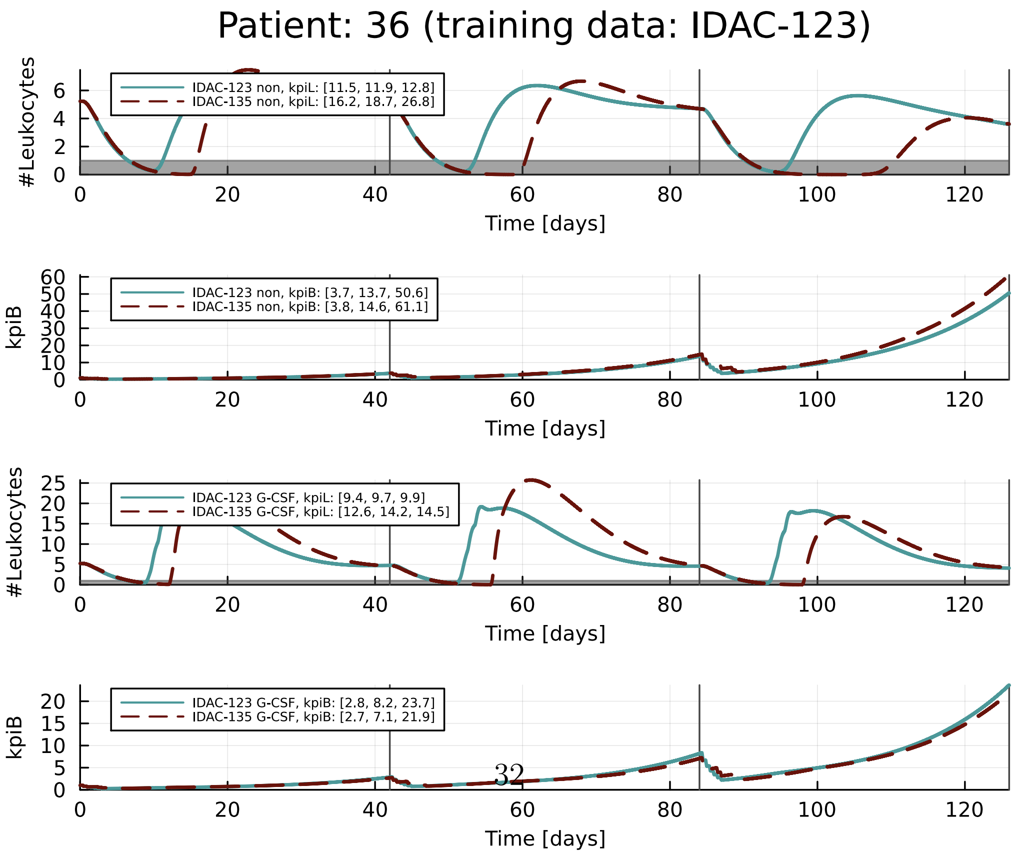
Patient: 36 (training data: IDAC-123)
IDAC-123 non, kpiL: [11.5, 11.9, 12.8]
IDAC-135 non, kpiL: [16.2, 18.7, 26.8]
IDAC-123 non, kpiB: [3.7, 13.7, 50.6]
IDAC-135 non, kpiB: [3.8, 14.6, 61.1]
IDAC-123 G-CSF, kpiL: [9.4, 9.7, 9.9]
IDAC-135 G-CSF, kpiL: [12.6, 14.2, 14.5]
IDAC-123 G-CSF, kpiB: [2.8, 8.2, 23.7]
IDAC-135 G-CSF, kpiB: [2.7, 7.1, 21.9]
#Leukocytes
kpiB
Time [days]

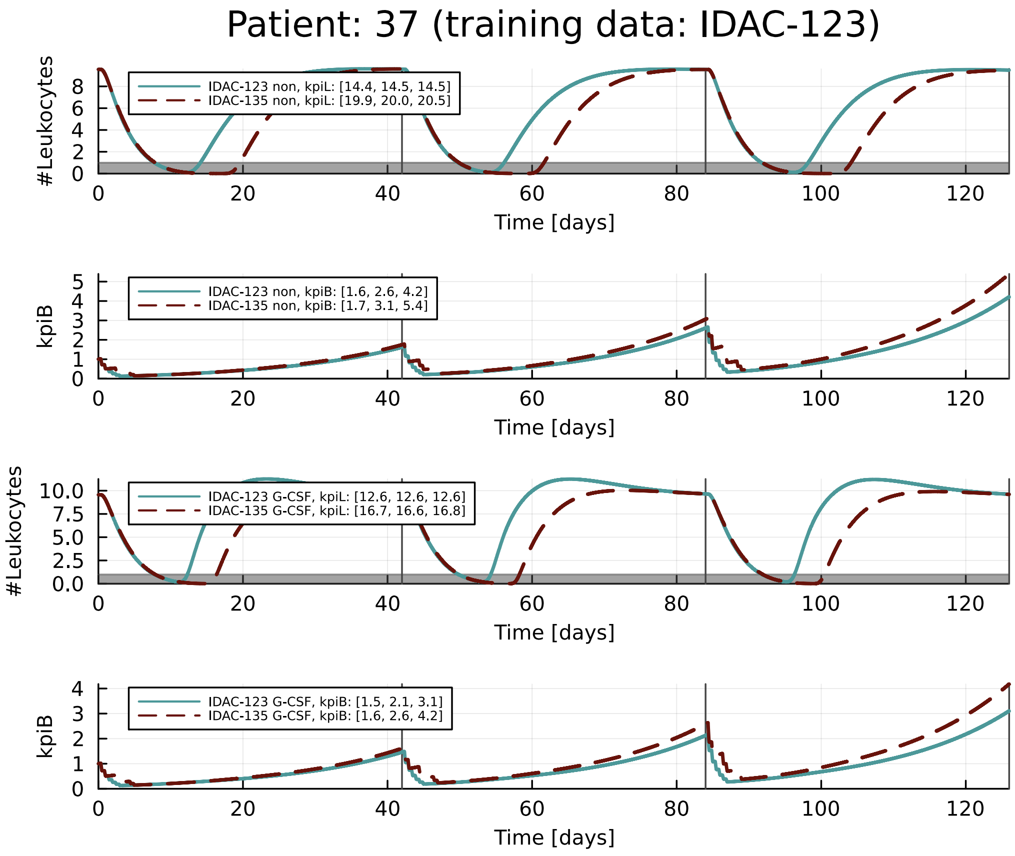
Patient: 37 (training data: IDAC-123)
IDAC-123 non, kpiL: [14.4, 14.5, 14.5]
IDAC-135 non, kpiL: [19.9, 20.0, 20.5]
IDAC-123 non, kpiB: [1.6, 2.6, 4.2]
IDAC-135 non, kpiB: [1.7, 3.1, 5.4]
IDAC-123 G-CSF, kpiL: [12.6, 12.6, 12.6]
IDAC-135 G-CSF, kpiL: [16.7, 16.6, 16.8]
IDAC-123 G-CSF, kpiB: [1.5, 2.1, 3.1]
IDAC-135 G-CSF, kpiB: [1.6, 2.6, 4.2]
#Leukocytes
kpiB
Time [days]

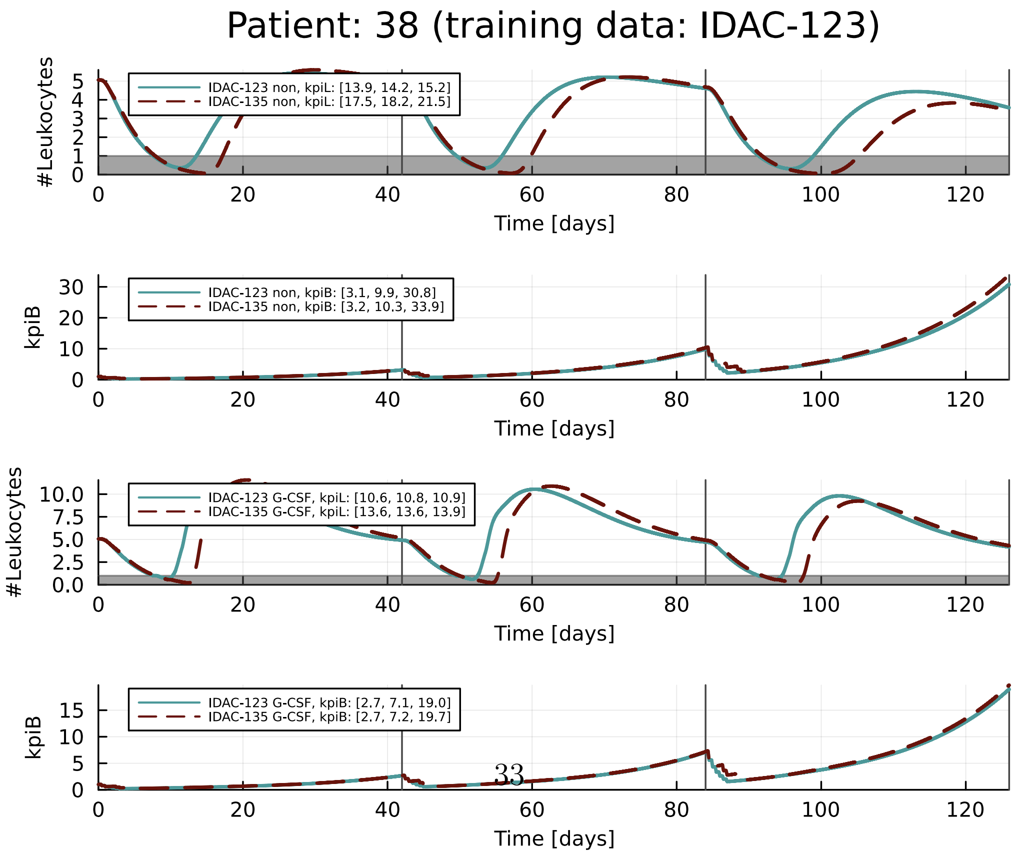
Patient: 38 (training data: IDAC-123)
IDAC-123 non, kpiL: [13.9, 14.2, 15.2]
IDAC-135 non, kpiL: [17.5, 18.2, 21.5]
IDAC-123 non, kpiB: [3.1, 9.9, 30.8]
IDAC-135 non, kpiB: [3.2, 10.3, 33.9]
IDAC-123 G-CSF, kpiL: [10.6, 10.8, 10.9]
IDAC-135 G-CSF, kpiL: [13.6, 13.6, 13.9]
IDAC-123 G-CSF, kpiB: [2.7, 7.1, 19.0]
IDAC-135 G-CSF, kpiB: [2.7, 7.2, 19.7]
#Leukocytes
kpiB
Time [days]

Patient: 39 (training data: IDAC-123)

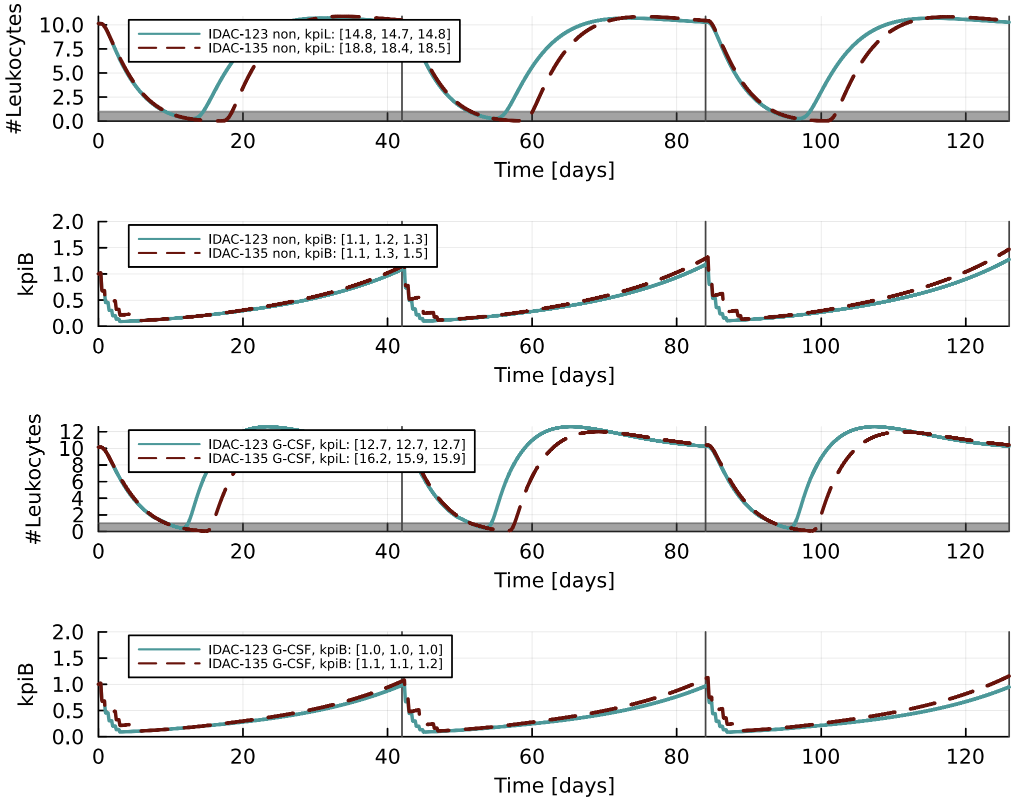

Patient: 40 (training data: IDAC-123)

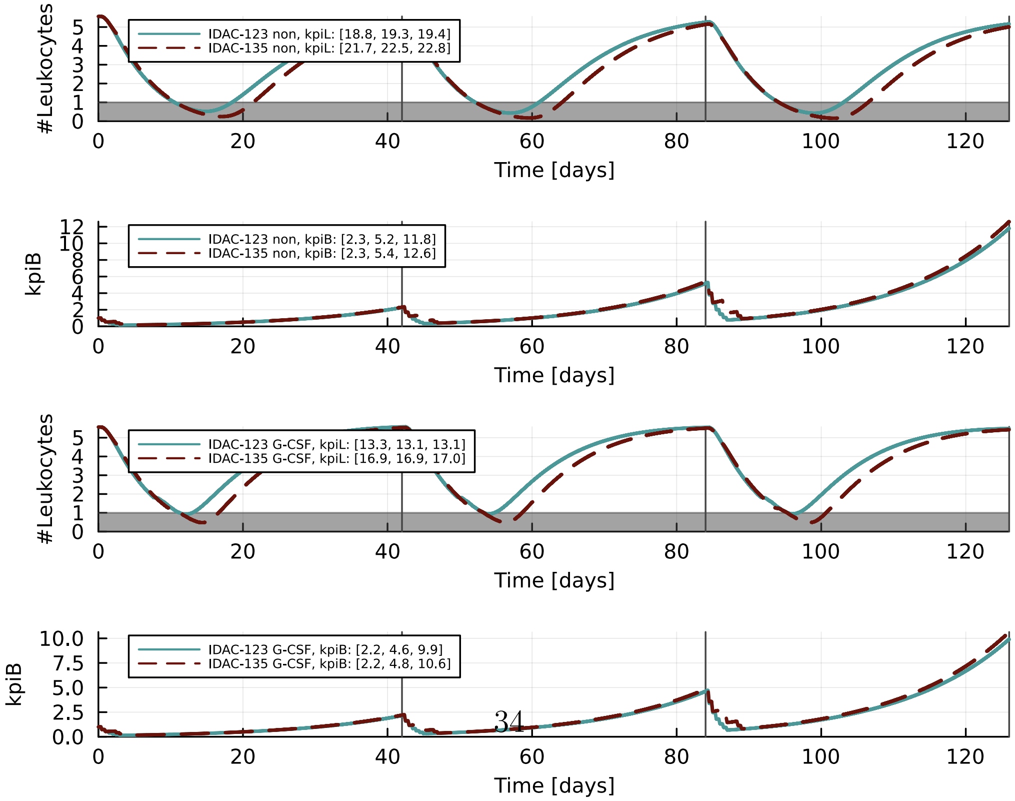

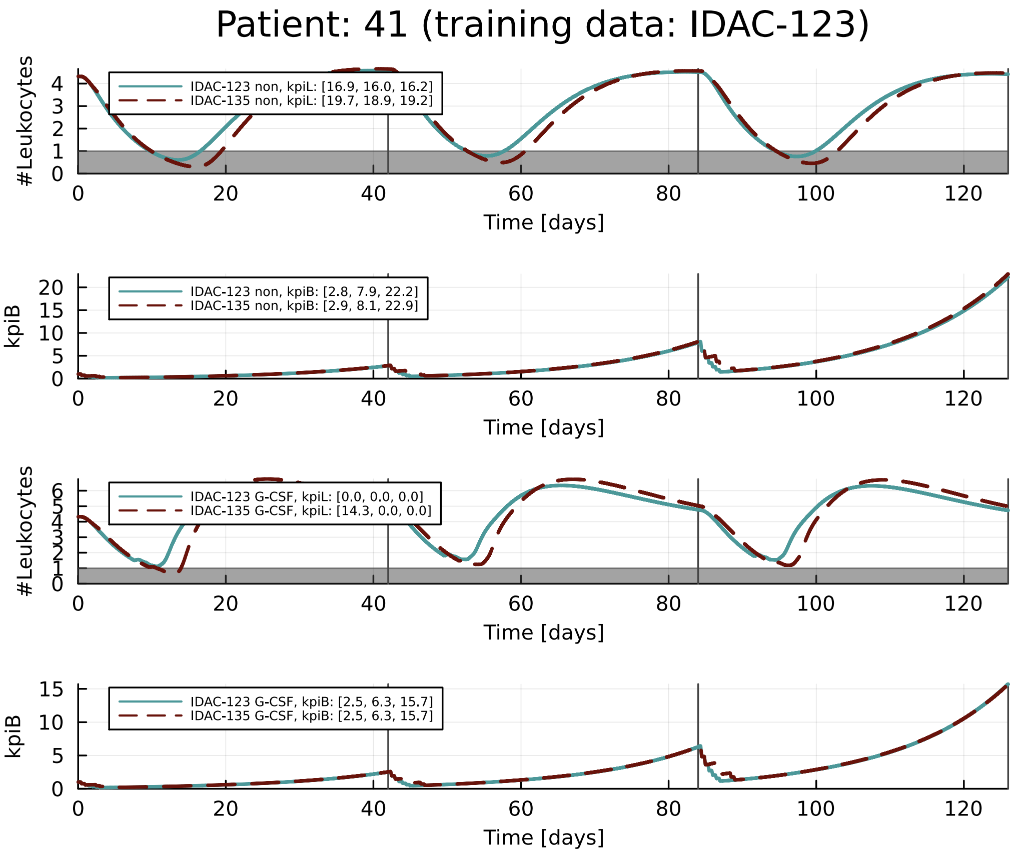
Patient: 41 (training data: IDAC-123)
IDAC-123 non, kpiL: [16.9, 16.0, 16.2]
IDAC-135 non, kpiL: [19.7, 18.9, 19.2]
IDAC-123 non, kpiB: [2.8, 7.9, 22.2]
IDAC-135 non, kpiB: [2.9, 8.1, 22.9]
IDAC-123 G-CSF, kpiL: [0.0, 0.0, 0.0]
IDAC-135 G-CSF, kpiL: [14.3, 0.0, 0.0]
IDAC-123 G-CSF, kpiB: [2.5, 6.3, 15.7]
IDAC-135 G-CSF, kpiB: [2.5, 6.3, 15.7]
#Leukocytes
kpiB
Time [days]

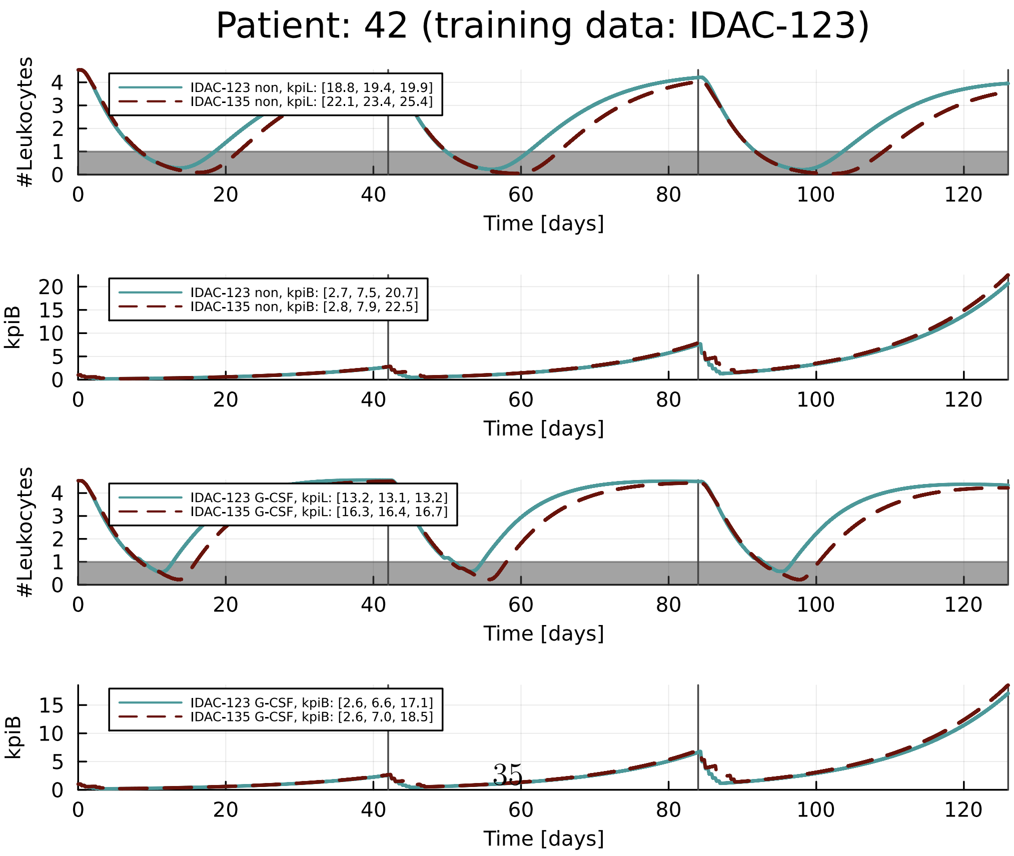
Patient: 42 (training data: IDAC-123)
IDAC-123 non, kpiL: [18.8, 19.4, 19.9]
IDAC-135 non, kpiL: [22.1, 23.4, 25.4]
IDAC-123 non, kpiB: [2.7, 7.5, 20.7]
IDAC-135 non, kpiB: [2.8, 7.9, 22.5]
IDAC-123 G-CSF, kpiL: [13.2, 13.1, 13.2]
IDAC-135 G-CSF, kpiL: [16.3, 16.4, 16.7]
IDAC-123 G-CSF, kpiB: [2.6, 6.6, 17.1]
IDAC-135 G-CSF, kpiB: [2.6, 7.0, 18.5]
#Leukocytes
kpiB
Time [days]

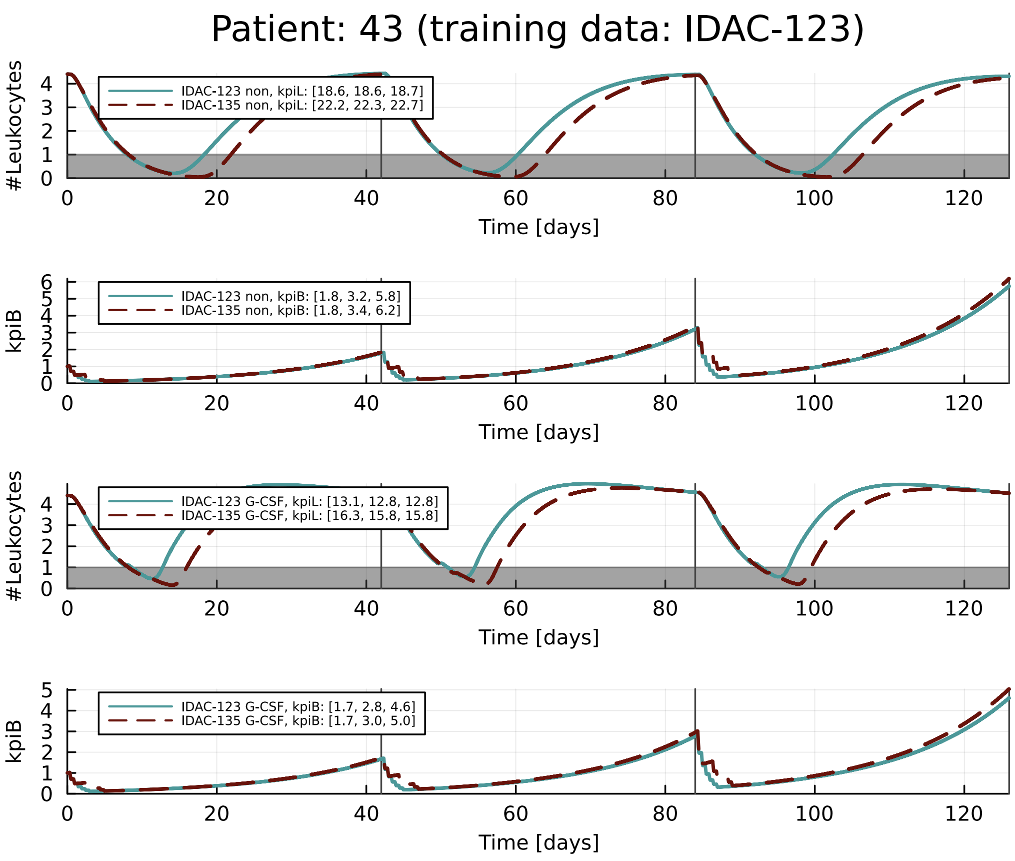
Patient: 43 (training data: IDAC-123)
IDAC-123 non, kpiL: [18.6, 18.6, 18.7]
IDAC-135 non, kpiL: [22.2, 22.3, 22.7]
#Leukocytes
Time [days]
IDAC-123 non, kpiB: [1.8, 3.2, 5.8]
IDAC-135 non, kpiB: [1.8, 3.4, 6.2]
kpiB
Time [days]
IDAC-123 G-CSF, kpiL: [13.1, 12.8, 12.8]
IDAC-135 G-CSF, kpiL: [16.3, 15.8, 15.8]
#Leukocytes
Time [days]
IDAC-123 G-CSF, kpiB: [1.7, 2.8, 4.6]
IDAC-135 G-CSF, kpiB: [1.7, 3.0, 5.0]
kpiB
Time [days]

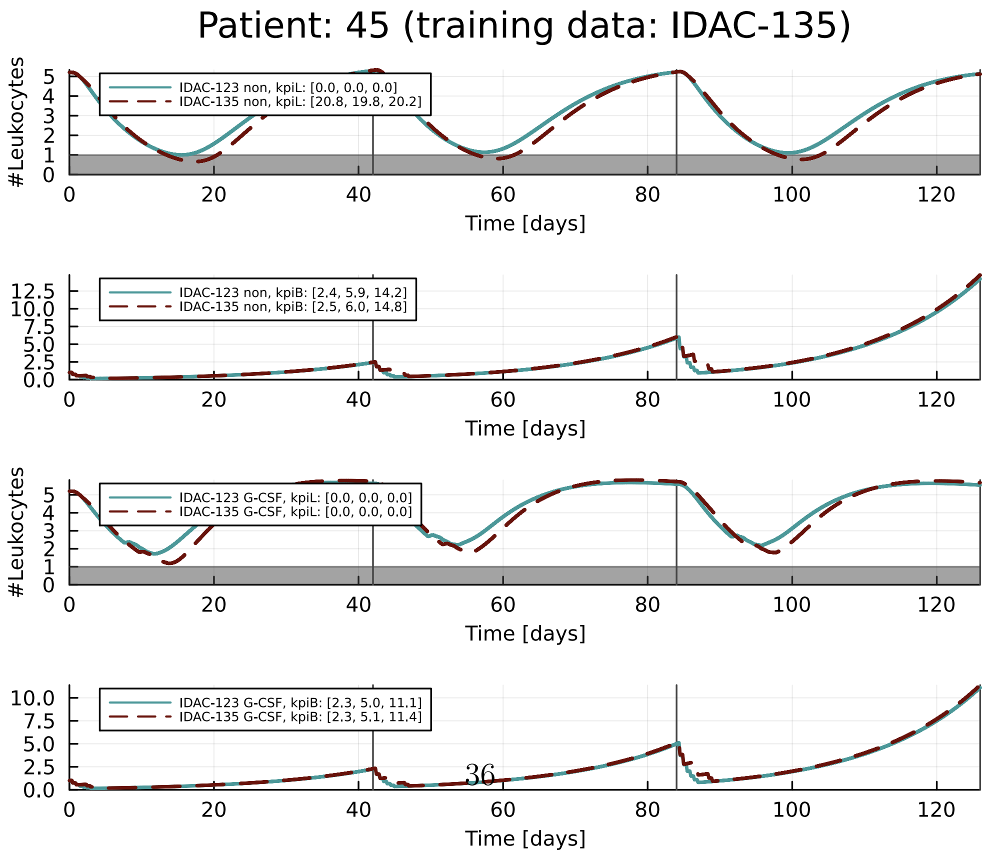
Patient: 45 (training data: IDAC-135)
IDAC-123 non, kpiL: [0.0, 0.0, 0.0]
IDAC-135 non, kpiL: [20.8, 19.8, 20.2]
#Leukocytes
Time [days]
IDAC-123 non, kpiB: [2.4, 5.9, 14.2]
IDAC-135 non, kpiB: [2.5, 6.0, 14.8]
kpiB
Time [days]
IDAC-123 G-CSF, kpiL: [0.0, 0.0, 0.0]
IDAC-135 G-CSF, kpiL: [0.0, 0.0, 0.0]
#Leukocytes
Time [days]
IDAC-123 G-CSF, kpiB: [2.3, 5.0, 11.1]
IDAC-135 G-CSF, kpiB: [2.3, 5.1, 11.4]
kpiB
Time [days]

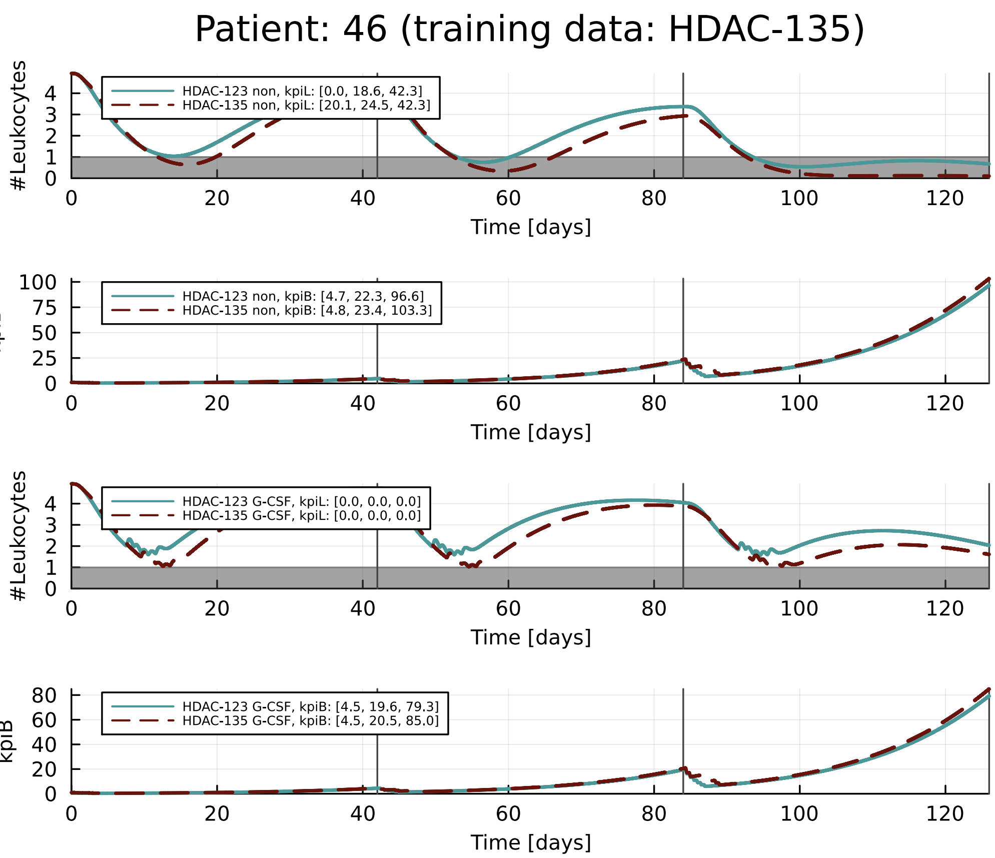
Patient: 46 (training data: HDAC-135)
#Leukocytes
HDAC-123 non, kpiL: [0.0, 18.6, 42.3]
HDAC-135 non, kpiL: [20.1, 24.5, 42.3]
Time [days]
kpiB
HDAC-123 non, kpiB: [4.7, 22.3, 96.6]
HDAC-135 non, kpiB: [4.8, 23.4, 103.3]
Time [days]
#Leukocytes
HDAC-123 G-CSF, kpiL: [0.0, 0.0, 0.0]
HDAC-135 G-CSF, kpiL: [0.0, 0.0, 0.0]
Time [days]
kpiB
HDAC-123 G-CSF, kpiB: [4.5, 19.6, 79.3]
HDAC-135 G-CSF, kpiB: [4.5, 20.5, 85.0]
Time [days]

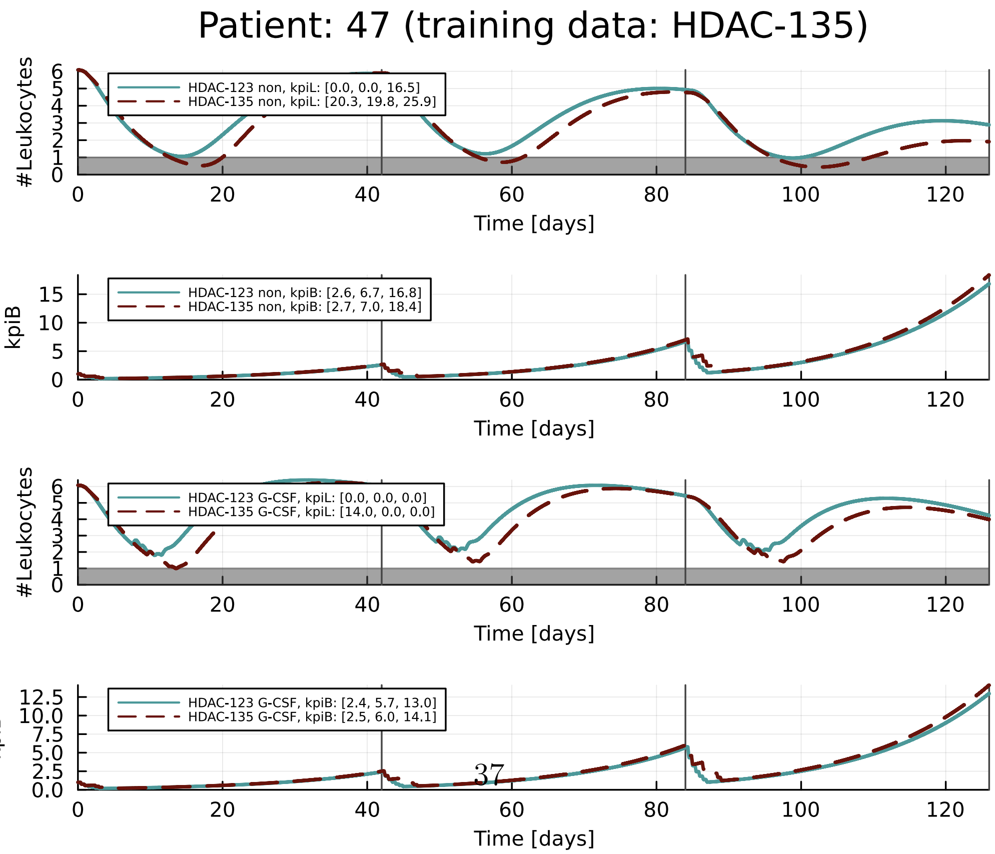
Patient: 47 (training data: HDAC-135)
#Leukocytes
HDAC-123 non, kpiL: [0.0, 0.0, 16.5]
HDAC-135 non, kpiL: [20.3, 19.8, 25.9]
Time [days]
kpiB
HDAC-123 non, kpiB: [2.6, 6.7, 16.8]
HDAC-135 non, kpiB: [2.7, 7.0, 18.4]
Time [days]
#Leukocytes
HDAC-123 G-CSF, kpiL: [0.0, 0.0, 0.0]
HDAC-135 G-CSF, kpiL: [14.0, 0.0, 0.0]
Time [days]
kpiB
HDAC-123 G-CSF, kpiB: [2.4, 5.7, 13.0]
HDAC-135 G-CSF, kpiB: [2.5, 6.0, 14.1]
Time [days]

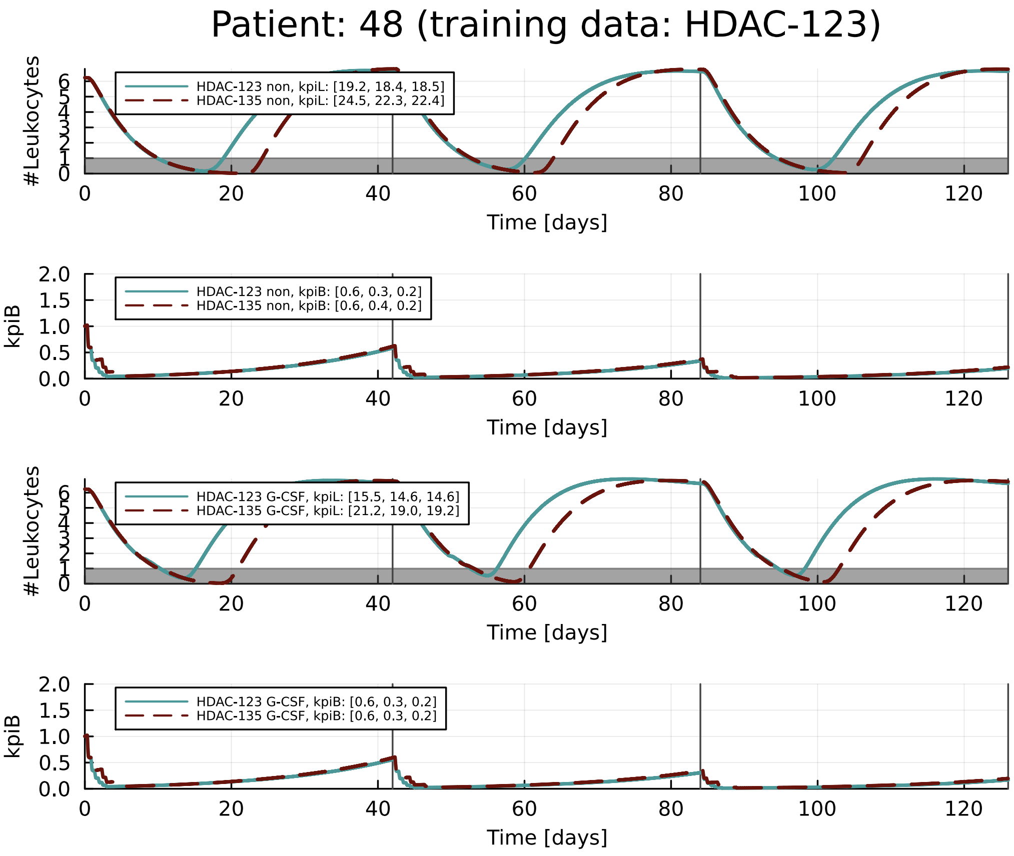
Patient: 48 (training data: HDAC-123)
HDAC-123 non, kpiL: [19.2, 18.4, 18.5]
HDAC-135 non, kpiL: [24.5, 22.3, 22.4]
#Leukocytes
Time [days]
HDAC-123 non, kpiB: [0.6, 0.3, 0.2]
HDAC-135 non, kpiB: [0.6, 0.4, 0.2]
kpiB
Time [days]
HDAC-123 G-CSF, kpiL: [15.5, 14.6, 14.6]
HDAC-135 G-CSF, kpiL: [21.2, 19.0, 19.2]
#Leukocytes
Time [days]
HDAC-123 G-CSF, kpiB: [0.6, 0.3, 0.2]
HDAC-135 G-CSF, kpiB: [0.6, 0.3, 0.2]
kpiB
Time [days]

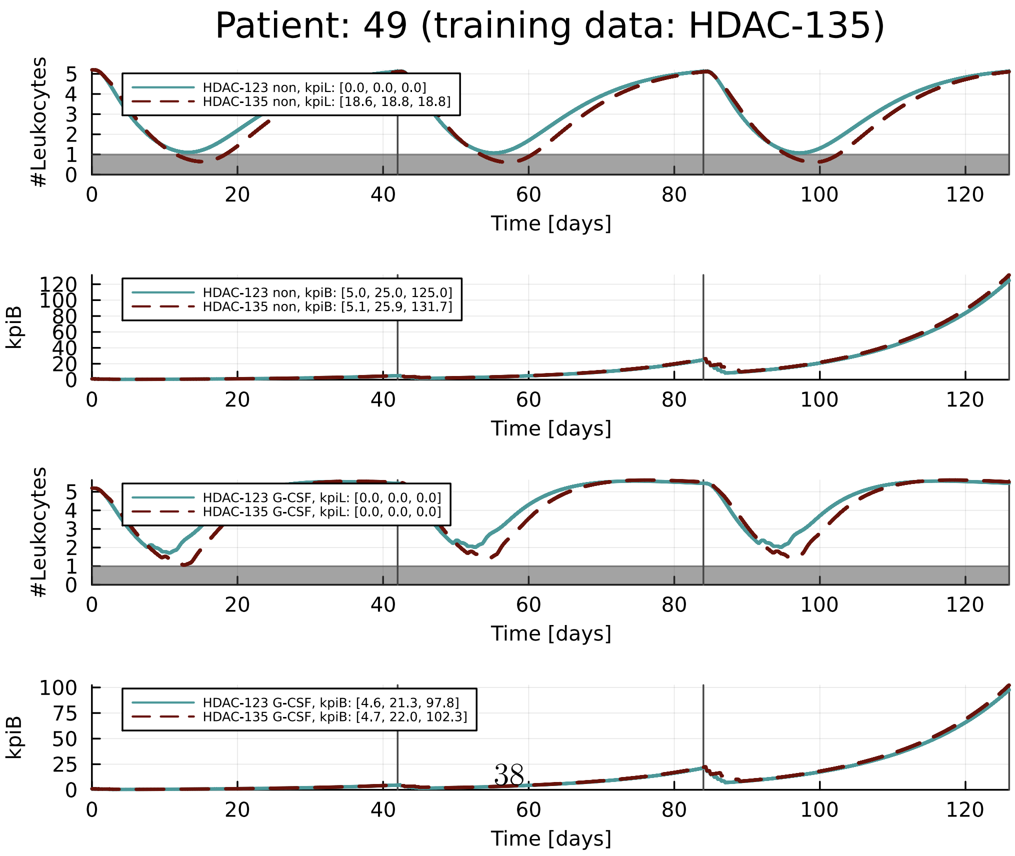
Patient: 49 (training data: HDAC-135)
HDAC-123 non, kpiL: [0.0, 0.0, 0.0]
HDAC-135 non, kpiL: [18.6, 18.8, 18.8]
#Leukocytes
Time [days]
HDAC-123 non, kpiB: [5.0, 25.0, 125.0]
HDAC-135 non, kpiB: [5.1, 25.9, 131.7]
kpiB
Time [days]
HDAC-123 G-CSF, kpiL: [0.0, 0.0, 0.0]
HDAC-135 G-CSF, kpiL: [0.0, 0.0, 0.0]
#Leukocytes
Time [days]
HDAC-123 G-CSF, kpiB: [4.6, 21.3, 97.8]
HDAC-135 G-CSF, kpiB: [4.7, 22.0, 102.3]
kpiB
Time [days]

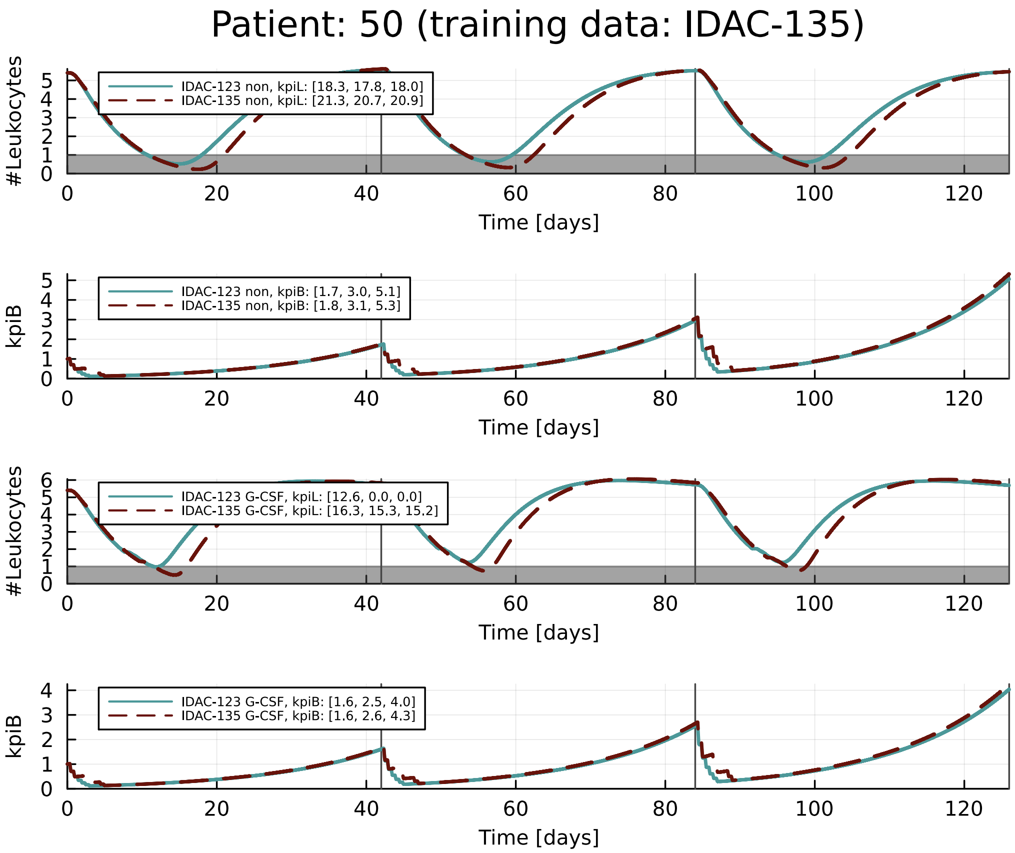
Patient: 50 (training data: IDAC-135)
IDAC-123 non, kpiL: [18.3, 17.8, 18.0]
IDAC-135 non, kpiL: [21.3, 20.7, 20.9]
IDAC-123 non, kpiB: [1.7, 3.0, 5.1]
IDAC-135 non, kpiB: [1.8, 3.1, 5.3]
IDAC-123 G-CSF, kpiL: [12.6, 0.0, 0.0]
IDAC-135 G-CSF, kpiL: [16.3, 15.3, 15.2]
IDAC-123 G-CSF, kpiB: [1.6, 2.5, 4.0]
IDAC-135 G-CSF, kpiB: [1.6, 2.6, 4.3]
#Leukocytes
kpiB
Time [days]

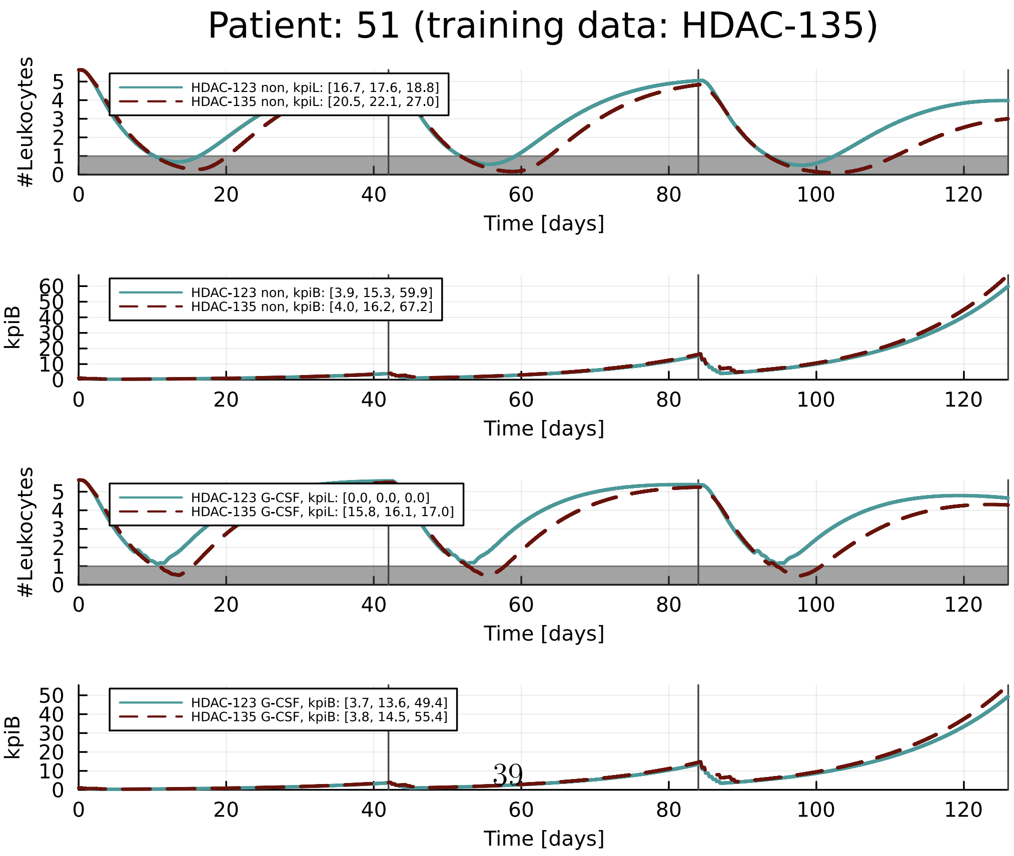
Patient: 51 (training data: HDAC-135)
HDAC-123 non, kpiL: [16.7, 17.6, 18.8]
HDAC-135 non, kpiL: [20.5, 22.1, 27.0]
HDAC-123 non, kpiB: [3.9, 15.3, 59.9]
HDAC-135 non, kpiB: [4.0, 16.2, 67.2]
HDAC-123 G-CSF, kpiL: [0.0, 0.0, 0.0]
HDAC-135 G-CSF, kpiL: [15.8, 16.1, 17.0]
HDAC-123 G-CSF, kpiB: [3.7, 13.6, 49.4]
HDAC-135 G-CSF, kpiB: [3.8, 14.5, 55.4]
#Leukocytes
kpiB
Time [days]

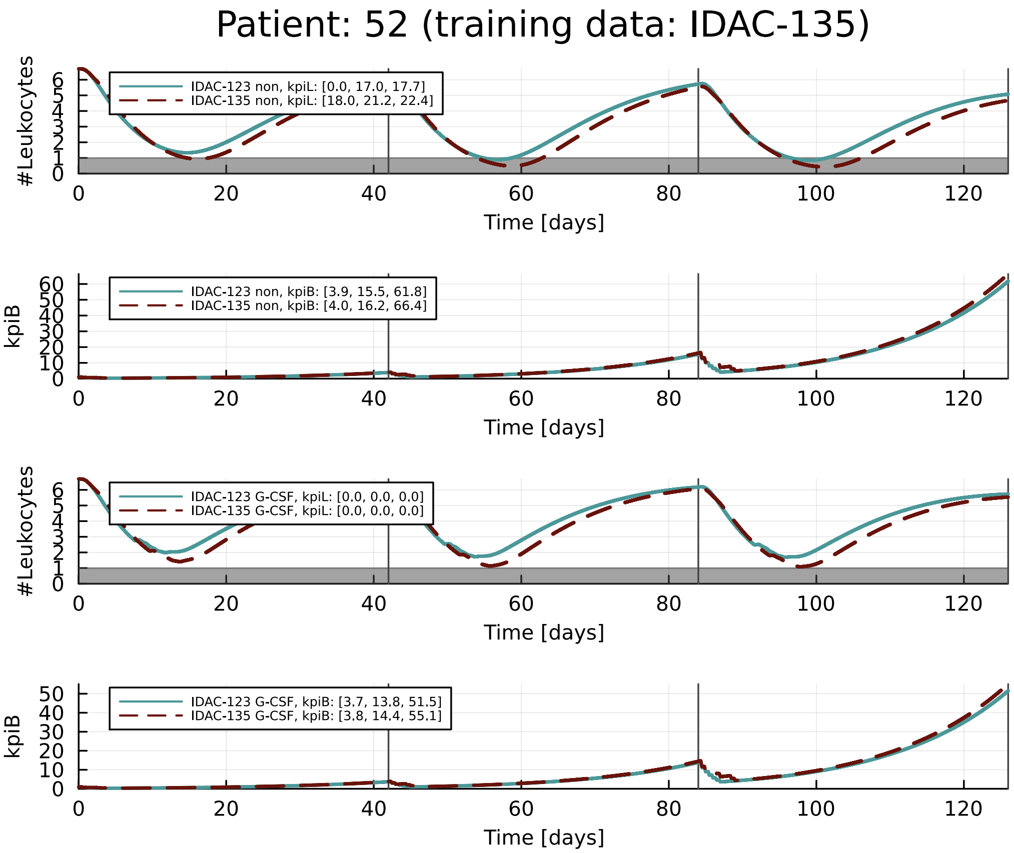
Patient: 52 (training data: IDAC-135)
IDAC-123 non, kpiL: [0.0, 17.0, 17.7]
IDAC-135 non, kpiL: [18.0, 21.2, 22.4]
#Leukocytes
Time [days]
IDAC-123 non, kpiB: [3.9, 15.5, 61.8]
IDAC-135 non, kpiB: [4.0, 16.2, 66.4]
kpiB
Time [days]
IDAC-123 G-CSF, kpiL: [0.0, 0.0, 0.0]
IDAC-135 G-CSF, kpiL: [0.0, 0.0, 0.0]
#Leukocytes
Time [days]
IDAC-123 G-CSF, kpiB: [3.7, 13.8, 51.5]
IDAC-135 G-CSF, kpiB: [3.8, 14.4, 55.1]
kpiB
Time [days]

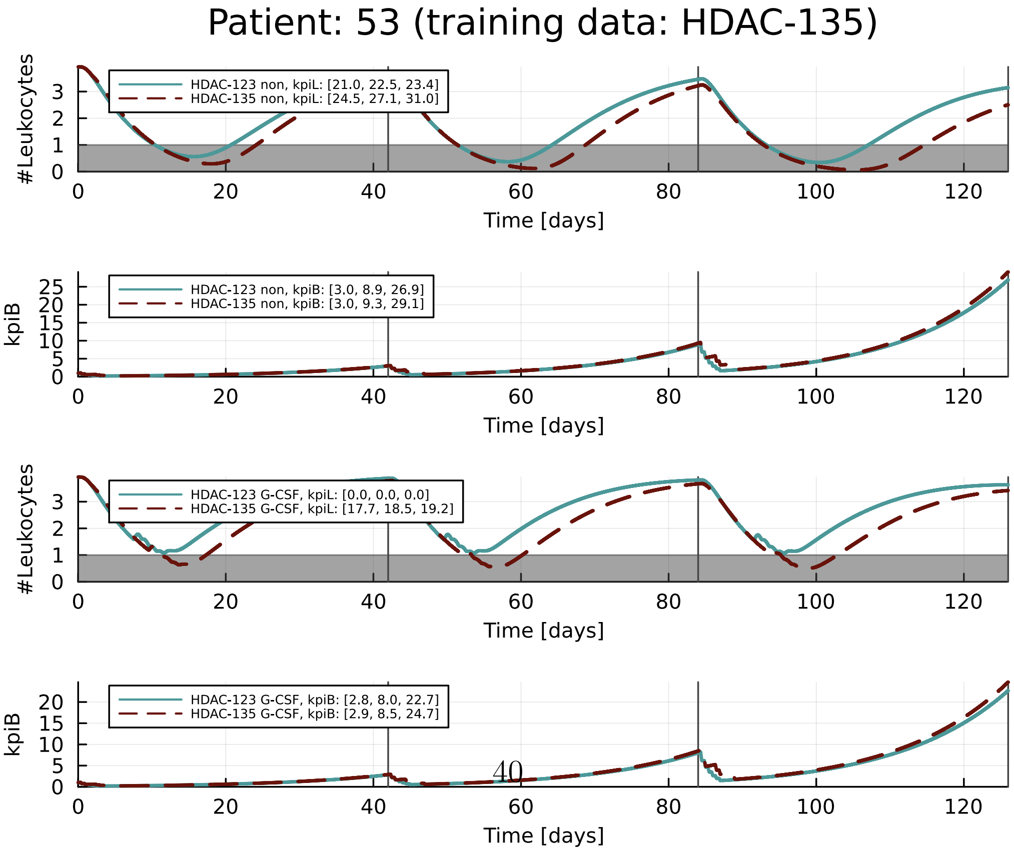
Patient: 53 (training data: HDAC-135)
HDAC-123 non, kpiL: [21.0, 22.5, 23.4]
HDAC-135 non, kpiL: [24.5, 27.1, 31.0]
#Leukocytes
Time [days]
HDAC-123 non, kpiB: [3.0, 8.9, 26.9]
HDAC-135 non, kpiB: [3.0, 9.3, 29.1]
kpiB
Time [days]
HDAC-123 G-CSF, kpiL: [0.0, 0.0, 0.0]
HDAC-135 G-CSF, kpiL: [17.7, 18.5, 19.2]
#Leukocytes
Time [days]
HDAC-123 G-CSF, kpiB: [2.8, 8.0, 22.7]
HDAC-135 G-CSF, kpiB: [2.9, 8.5, 24.7]
kpiB
Time [days]

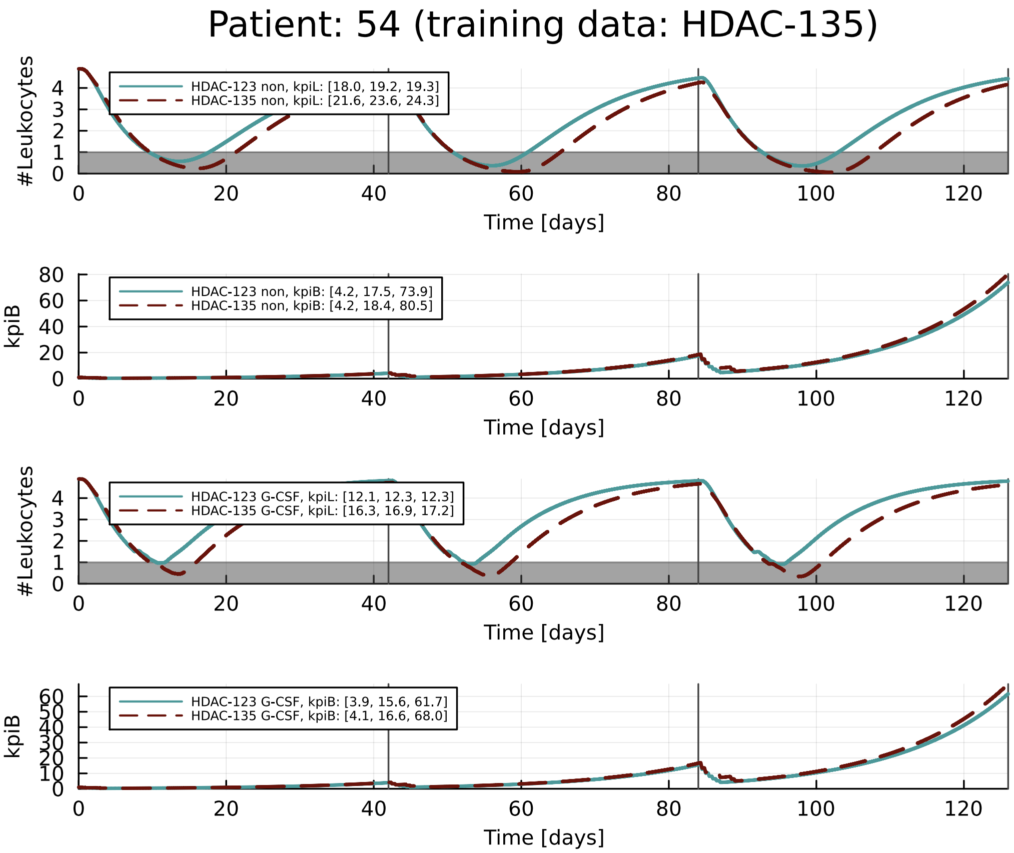
Patient: 54 (training data: HDAC-135)
HDAC-123 non, kpiL: [18.0, 19.2, 19.3]
HDAC-135 non, kpiL: [21.6, 23.6, 24.3]
#Leukocytes
Time [days]
HDAC-123 non, kpiB: [4.2, 17.5, 73.9]
HDAC-135 non, kpiB: [4.2, 18.4, 80.5]
kpiB
HDAC-123 G-CSF, kpiL: [12.1, 12.3, 12.3]
HDAC-135 G-CSF, kpiL: [16.3, 16.9, 17.2]
HDAC-123 G-CSF, kpiB: [3.9, 15.6, 61.7]
HDAC-135 G-CSF, kpiB: [4.1, 16.6, 68.0]

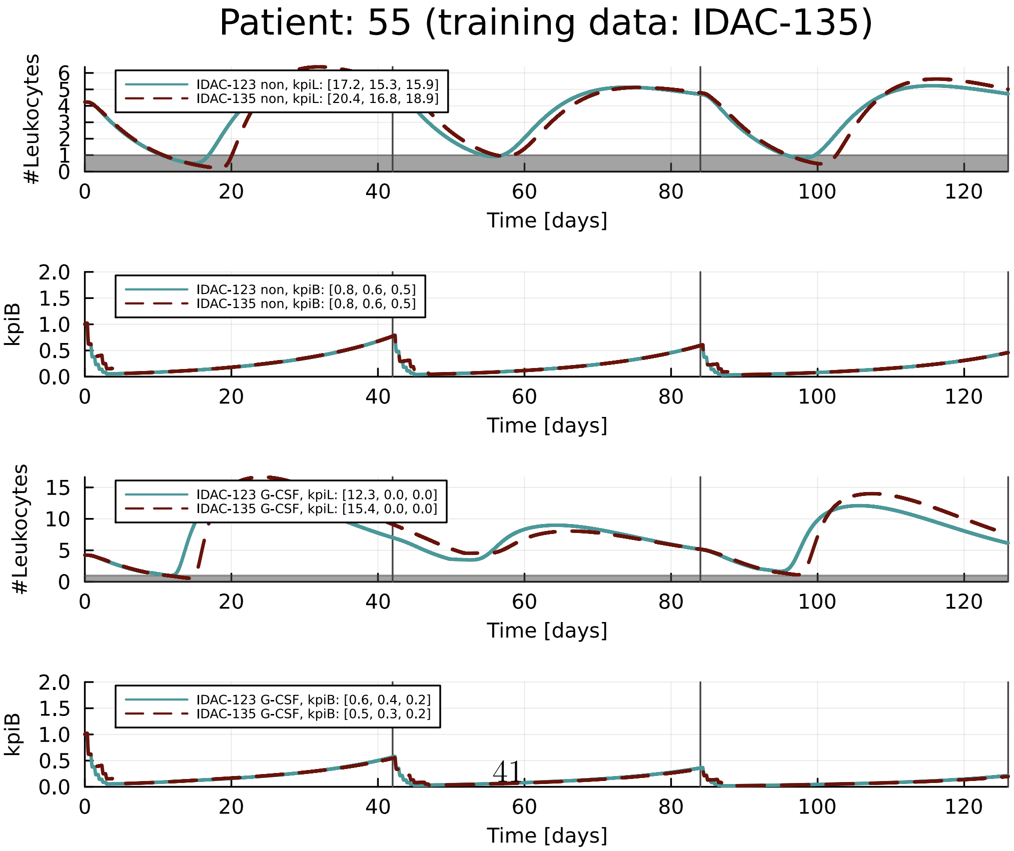
Patient: 55 (training data: IDAC-135)
IDAC-123 non, kpiL: [17.2, 15.3, 15.9]
IDAC-135 non, kpiL: [20.4, 16.8, 18.9]
#Leukocytes
Time [days]
IDAC-123 non, kpiB: [0.8, 0.6, 0.5]
IDAC-135 non, kpiB: [0.8, 0.6, 0.5]
kpiB
IDAC-123 G-CSF, kpiL: [12.3, 0.0, 0.0]
IDAC-135 G-CSF, kpiL: [15.4, 0.0, 0.0]
IDAC-123 G-CSF, kpiB: [0.6, 0.4, 0.2]
IDAC-135 G-CSF, kpiB: [0.5, 0.3, 0.2]

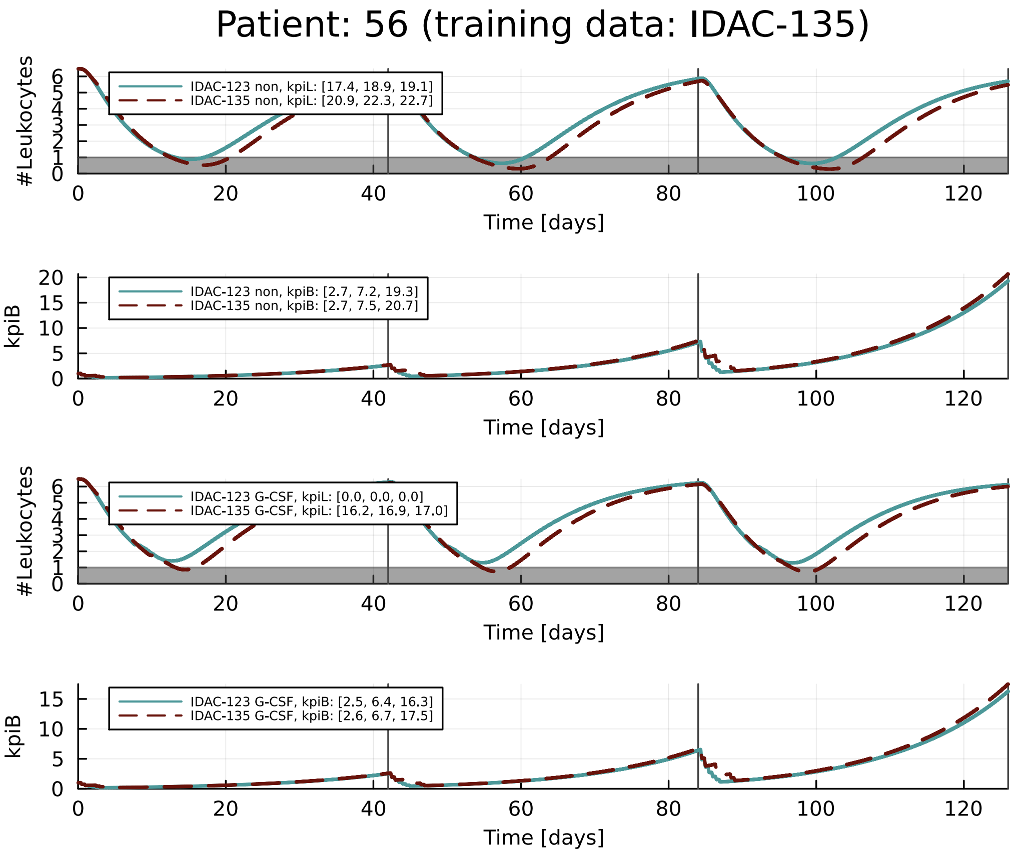
Patient: 56 (training data: IDAC-135)
IDAC-123 non, kpiL: [17.4, 18.9, 19.1]
IDAC-135 non, kpiL: [20.9, 22.3, 22.7]
IDAC-123 non, kpiB: [2.7, 7.2, 19.3]
IDAC-135 non, kpiB: [2.7, 7.5, 20.7]
IDAC-123 G-CSF, kpiL: [0.0, 0.0, 0.0]
IDAC-135 G-CSF, kpiL: [16.2, 16.9, 17.0]
IDAC-123 G-CSF, kpiB: [2.5, 6.4, 16.3]
IDAC-135 G-CSF, kpiB: [2.6, 6.7, 17.5]
#Leukocytes
kpiB
Time [days]

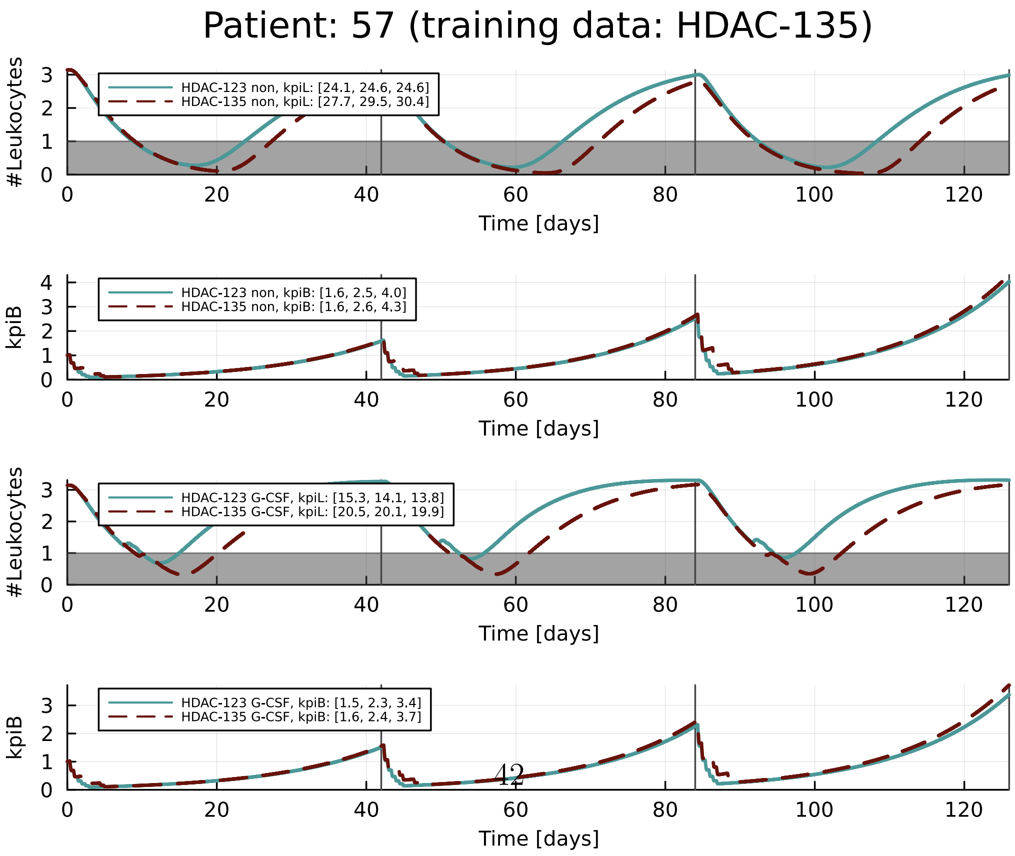
Patient: 57 (training data: HDAC-135)
HDAC-123 non, kpiL: [24.1, 24.6, 24.6]
HDAC-135 non, kpiL: [27.7, 29.5, 30.4]
HDAC-123 non, kpiB: [1.6, 2.5, 4.0]
HDAC-135 non, kpiB: [1.6, 2.6, 4.3]
HDAC-123 G-CSF, kpiL: [15.3, 14.1, 13.8]
HDAC-135 G-CSF, kpiL: [20.5, 20.1, 19.9]
HDAC-123 G-CSF, kpiB: [1.5, 2.3, 3.4]
HDAC-135 G-CSF, kpiB: [1.6, 2.4, 3.7]
#Leukocytes
kpiB
Time [days]

Patient: 58 (training data: IDAC-135)

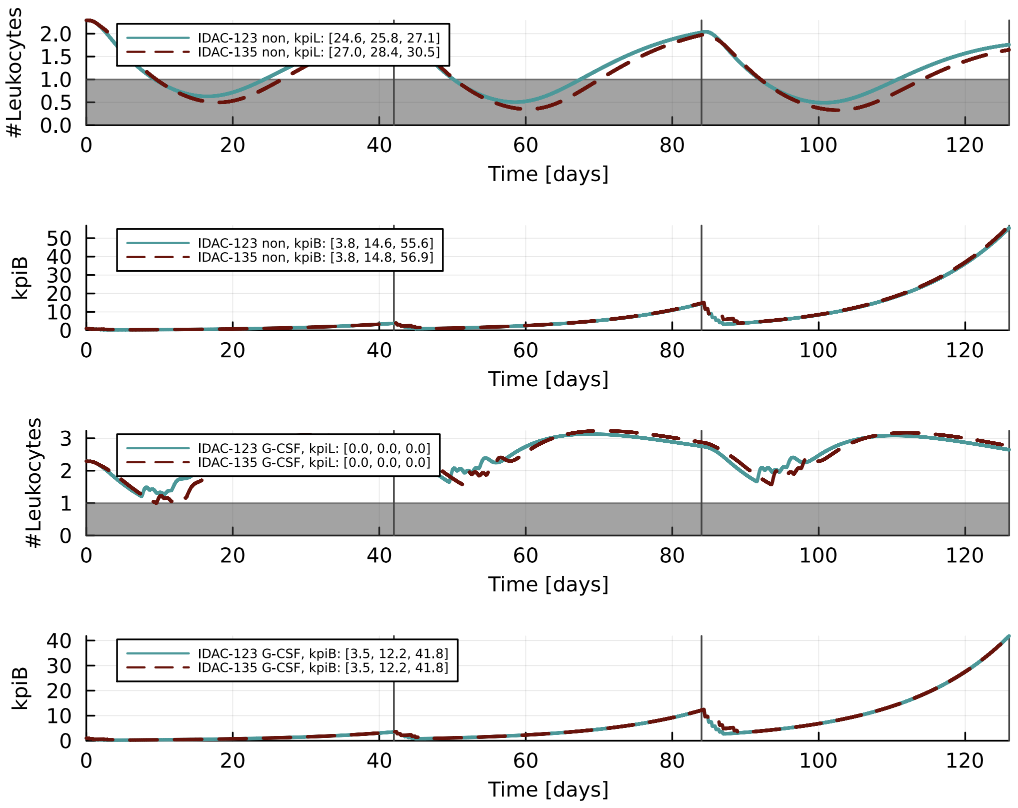

Patient: 59 (training data: IDAC-135)

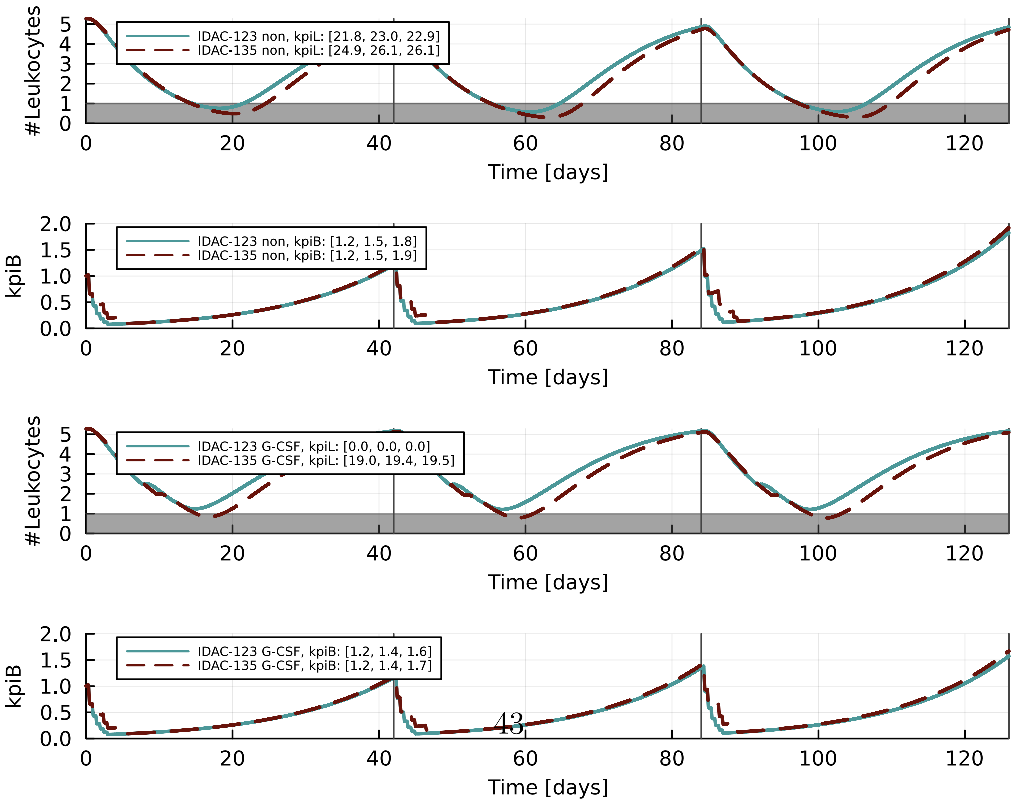

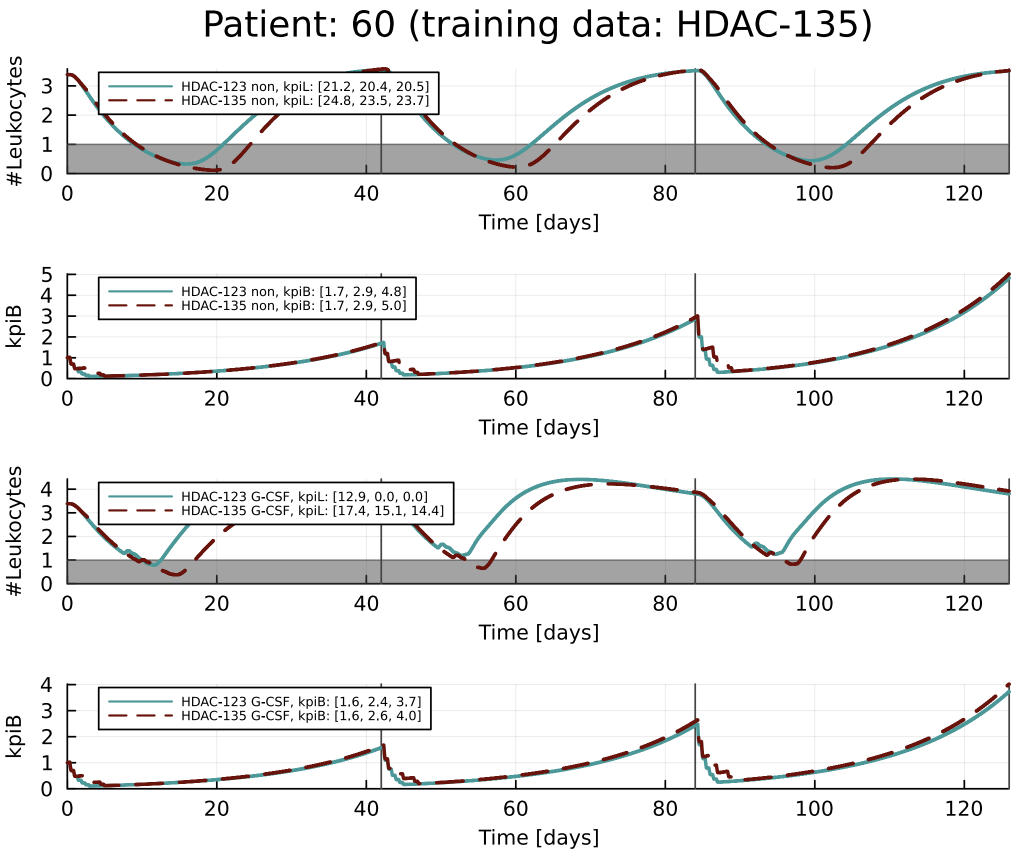
Patient: 60 (training data: HDAC-135)
HDAC-123 non, kpiL: [21.2, 20.4, 20.5]
HDAC-135 non, kpiL: [24.8, 23.5, 23.7]
#Leukocytes
Time [days]
HDAC-123 non, kpiB: [1.7, 2.9, 4.8]
HDAC-135 non, kpiB: [1.7, 2.9, 5.0]
kpiB
Time [days]
HDAC-123 G-CSF, kpiL: [12.9, 0.0, 0.0]
HDAC-135 G-CSF, kpiL: [17.4, 15.1, 14.4]
#Leukocytes
Time [days]
HDAC-123 G-CSF, kpiB: [1.6, 2.4, 3.7]
HDAC-135 G-CSF, kpiB: [1.6, 2.6, 4.0]
kpiB
Time [days]

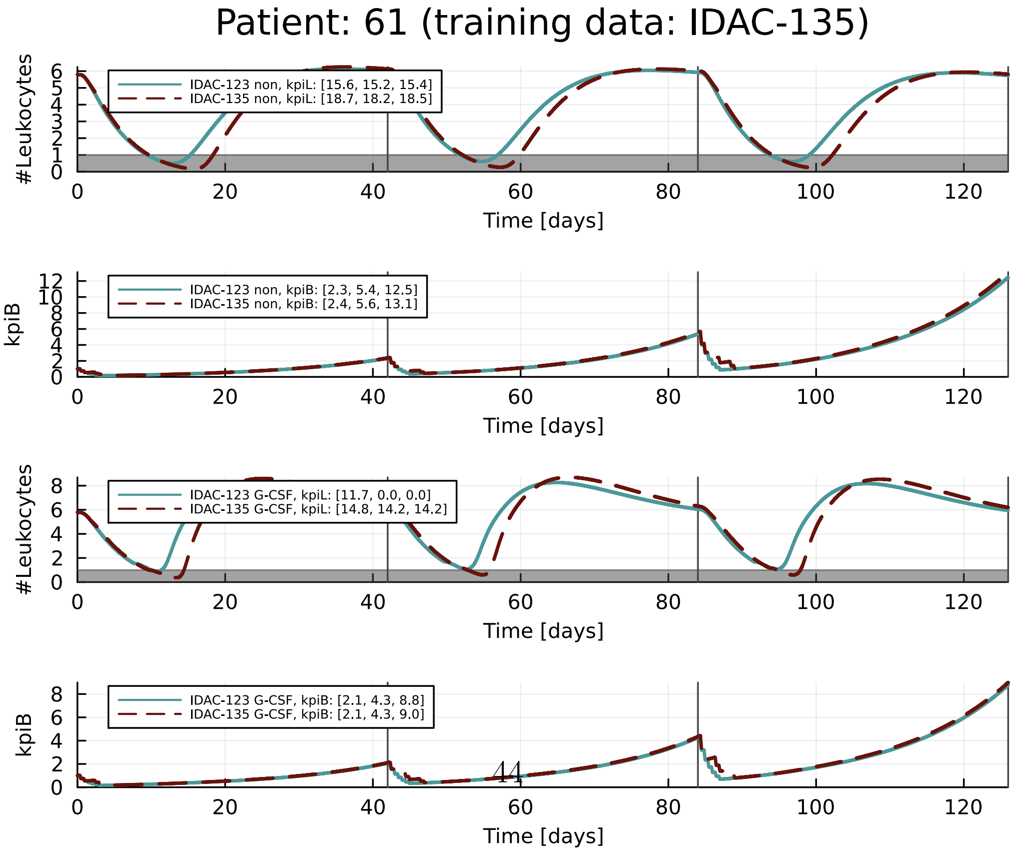
Patient: 61 (training data: IDAC-135)
IDAC-123 non, kpiL: [15.6, 15.2, 15.4]
IDAC-135 non, kpiL: [18.7, 18.2, 18.5]
#Leukocytes
Time [days]
IDAC-123 non, kpiB: [2.3, 5.4, 12.5]
IDAC-135 non, kpiB: [2.4, 5.6, 13.1]
kpiB
Time [days]
IDAC-123 G-CSF, kpiL: [11.7, 0.0, 0.0]
IDAC-135 G-CSF, kpiL: [14.8, 14.2, 14.2]
#Leukocytes
Time [days]
IDAC-123 G-CSF, kpiB: [2.1, 4.3, 8.8]
IDAC-135 G-CSF, kpiB: [2.1, 4.3, 9.0]
kpiB
Time [days]

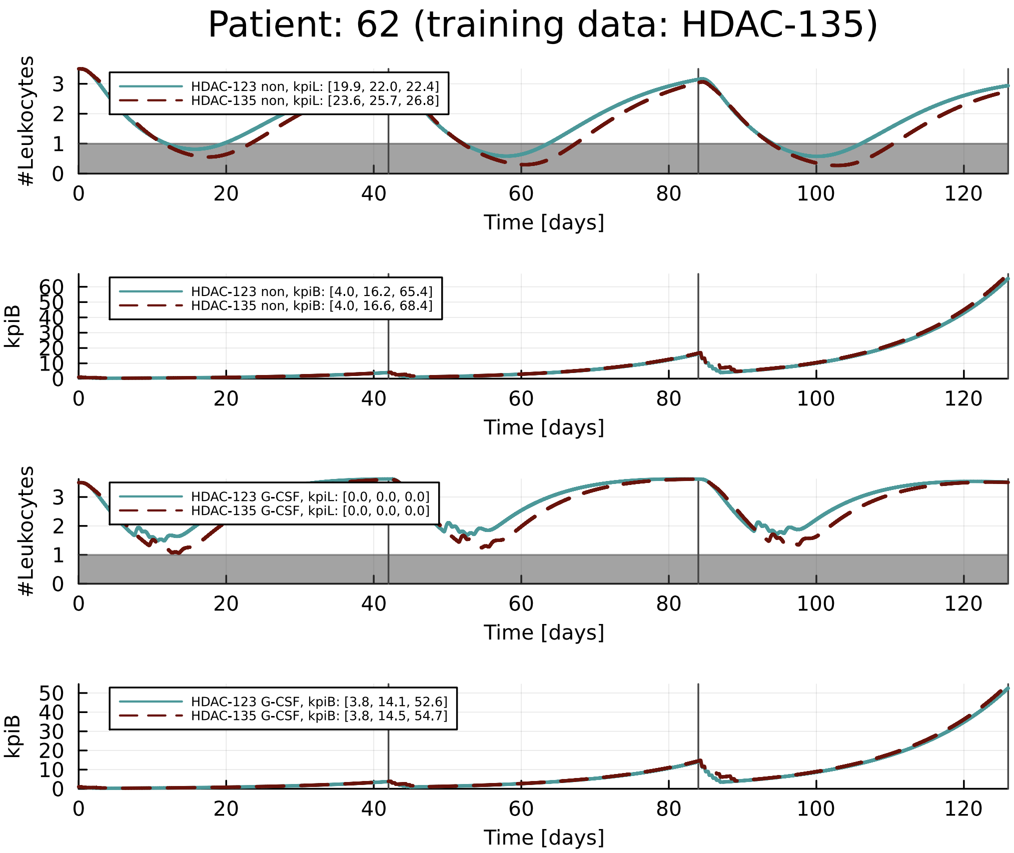
Patient: 62 (training data: HDAC-135)
HDAC-123 non, kpiL: [19.9, 22.0, 22.4]
HDAC-135 non, kpiL: [23.6, 25.7, 26.8]
HDAC-123 non, kpiB: [4.0, 16.2, 65.4]
HDAC-135 non, kpiB: [4.0, 16.6, 68.4]
HDAC-123 G-CSF, kpiL: [0.0, 0.0, 0.0]
HDAC-135 G-CSF, kpiL: [0.0, 0.0, 0.0]
HDAC-123 G-CSF, kpiB: [3.8, 14.1, 52.6]
HDAC-135 G-CSF, kpiB: [3.8, 14.5, 54.7]
#Leukocytes
kpiB
Time [days]

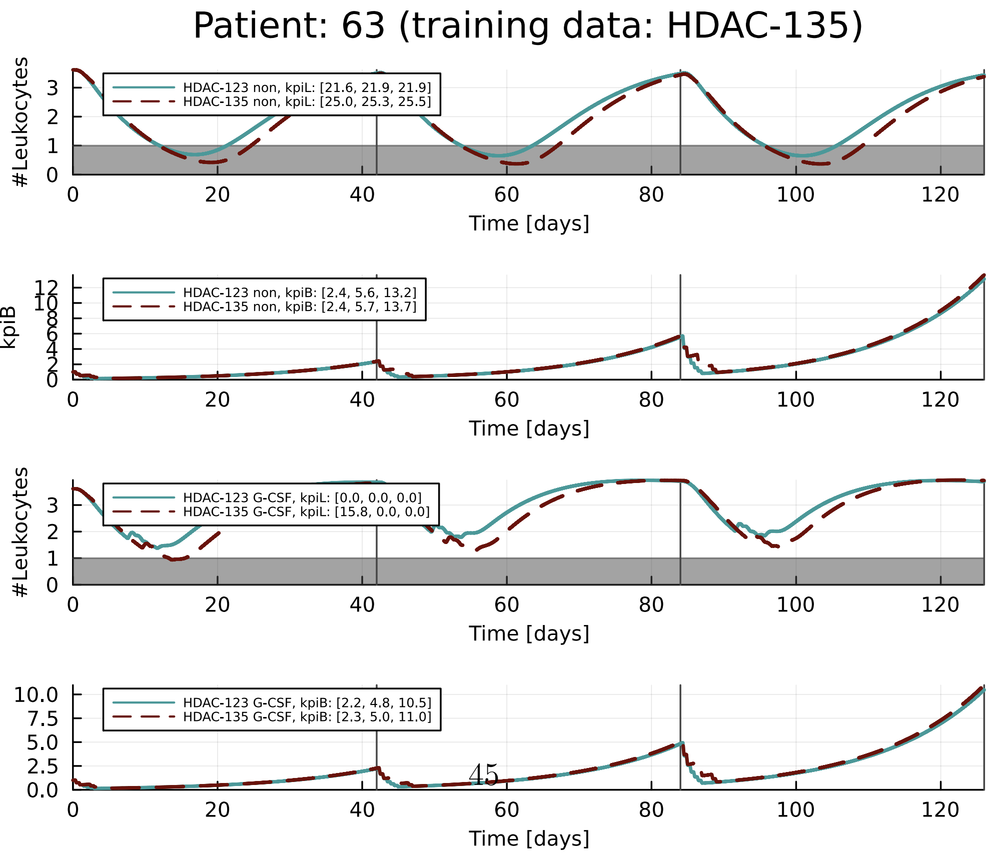
Patient: 63 (training data: HDAC-135)
HDAC-123 non, kpiL: [21.6, 21.9, 21.9]
HDAC-135 non, kpiL: [25.0, 25.3, 25.5]
HDAC-123 non, kpiB: [2.4, 5.6, 13.2]
HDAC-135 non, kpiB: [2.4, 5.7, 13.7]
HDAC-123 G-CSF, kpiL: [0.0, 0.0, 0.0]
HDAC-135 G-CSF, kpiL: [15.8, 0.0, 0.0]
HDAC-123 G-CSF, kpiB: [2.2, 4.8, 10.5]
HDAC-135 G-CSF, kpiB: [2.3, 5.0, 11.0]
#Leukocytes
kpiB
Time [days]

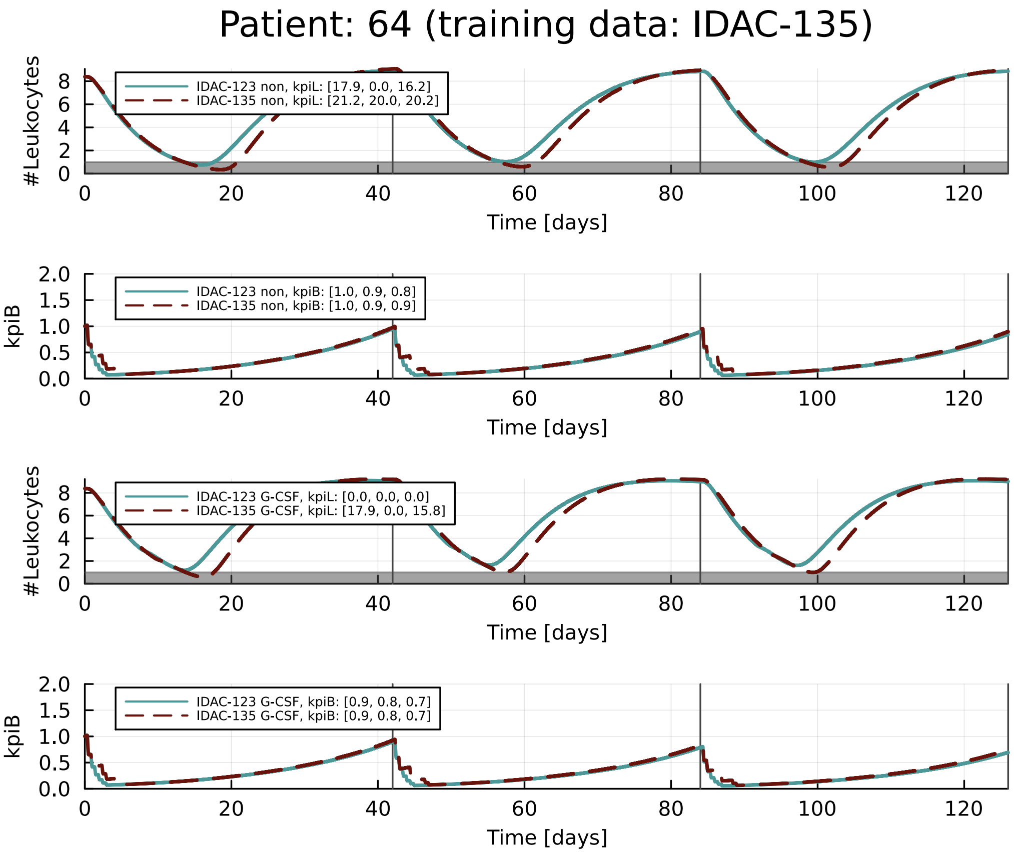
Patient: 64 (training data: IDAC-135)
IDAC-123 non, kpiL: [17.9, 0.0, 16.2]
IDAC-135 non, kpiL: [21.2, 20.0, 20.2]
#Leukocytes
Time [days]
IDAC-123 non, kpiB: [1.0, 0.9, 0.8]
IDAC-135 non, kpiB: [1.0, 0.9, 0.9]
kpiB
IDAC-123 G-CSF, kpiL: [0.0, 0.0, 0.0]
IDAC-135 G-CSF, kpiL: [17.9, 0.0, 15.8]
IDAC-123 G-CSF, kpiB: [0.9, 0.8, 0.7]
IDAC-135 G-CSF, kpiB: [0.9, 0.8, 0.7]

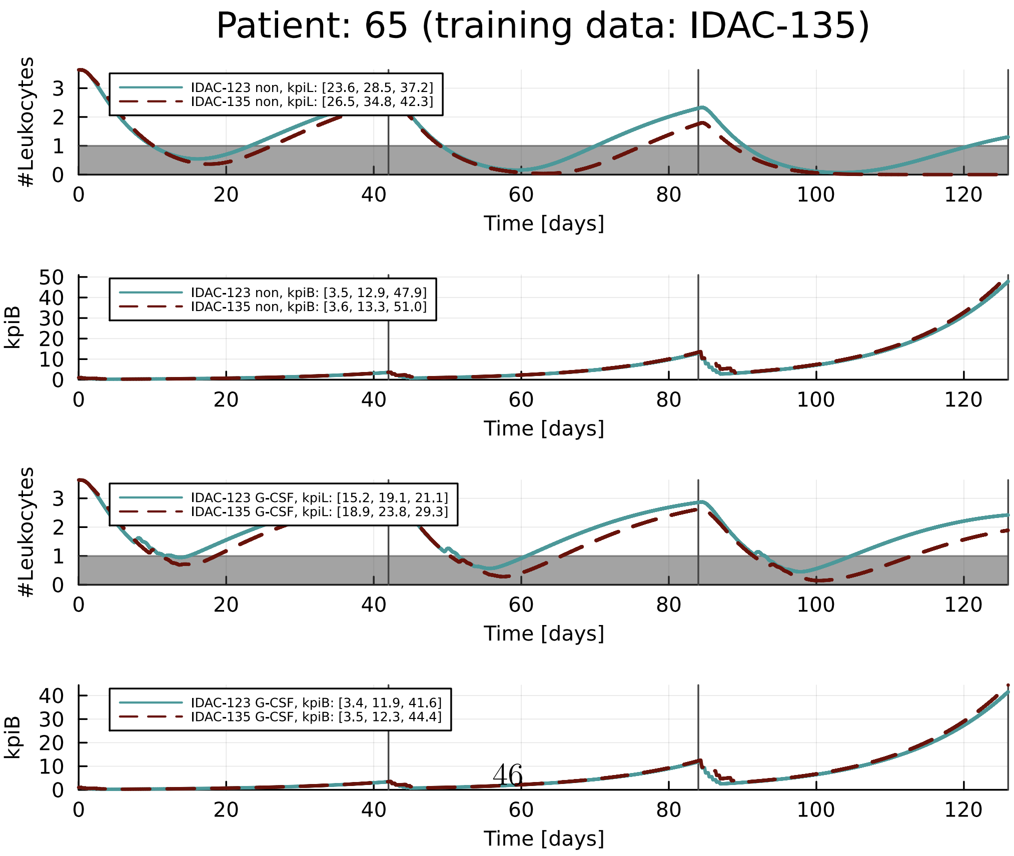
Patient: 65 (training data: IDAC-135)
IDAC-123 non, kpiL: [23.6, 28.5, 37.2]
IDAC-135 non, kpiL: [26.5, 34.8, 42.3]
#Leukocytes
Time [days]
IDAC-123 non, kpiB: [3.5, 12.9, 47.9]
IDAC-135 non, kpiB: [3.6, 13.3, 51.0]
kpiB
IDAC-123 G-CSF, kpiL: [15.2, 19.1, 21.1]
IDAC-135 G-CSF, kpiL: [18.9, 23.8, 29.3]
IDAC-123 G-CSF, kpiB: [3.4, 11.9, 41.6]
IDAC-135 G-CSF, kpiB: [3.5, 12.3, 44.4]

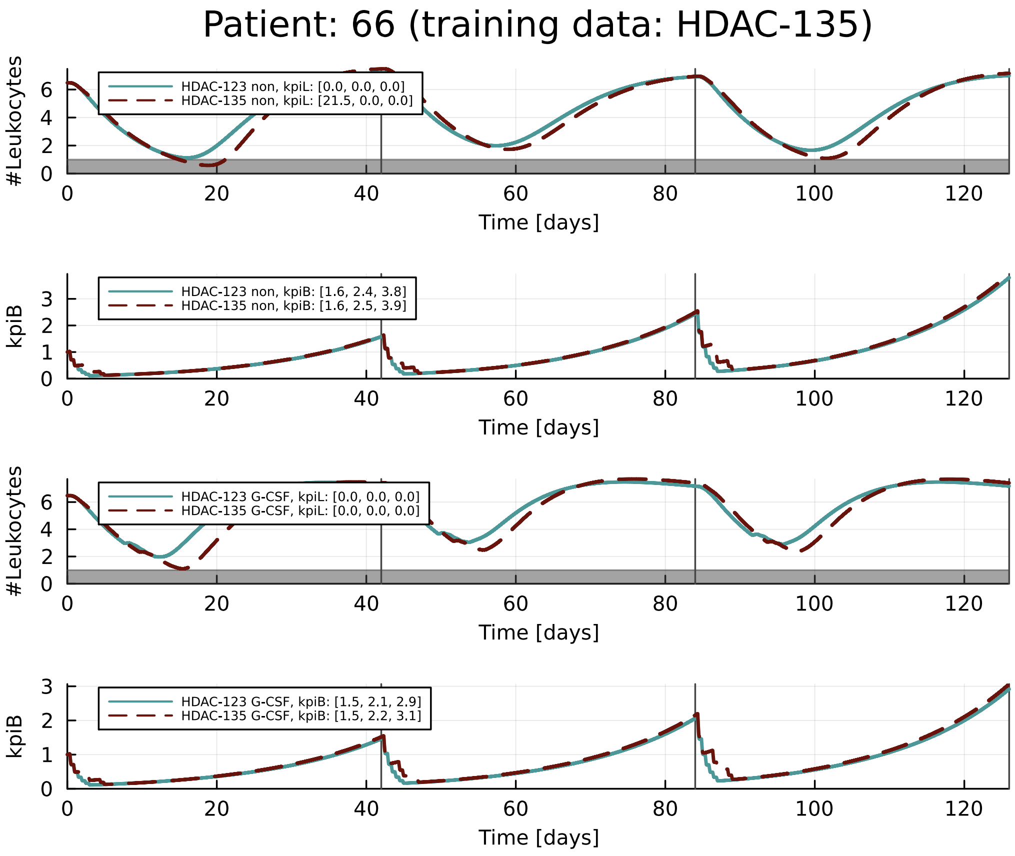
Patient: 66 (training data: HDAC-135)
HDAC-123 non, kpiL: [0.0, 0.0, 0.0]
HDAC-135 non, kpiL: [21.5, 0.0, 0.0]
#Leukocytes
Time [days]
HDAC-123 non, kpiB: [1.6, 2.4, 3.8]
HDAC-135 non, kpiB: [1.6, 2.5, 3.9]
kpiB
Time [days]
HDAC-123 G-CSF, kpiL: [0.0, 0.0, 0.0]
HDAC-135 G-CSF, kpiL: [0.0, 0.0, 0.0]
#Leukocytes
Time [days]
HDAC-123 G-CSF, kpiB: [1.5, 2.1, 2.9]
HDAC-135 G-CSF, kpiB: [1.5, 2.2, 3.1]
kpiB
Time [days]

References

[Brady and Enderling, 2019] Brady, R. and Enderling, H. (2019). Mathematical models of cancer: when to predict novel therapies, and when not to. *Bulletin of mathematical biology*, 81:3722–3731. Publisher: Springer.

[Chulián et al., 2022] Chulián, S., Martínez-Rubio, A., Rosa, M., and Pérez-García, V. M. (2022). Mathematical models of Leukaemia and its treatment: A review. *SeMA Journal*, 79(3):441–486. Publisher: Springer.

[Clairambault, 2009] Clairambault, J. (2009). Modelling physiological and pharmacological control on cell proliferation to optimise cancer treatments. *Mathematical Modelling of Natural Phenomena*, 4(3):12–67.

[Dumas et al., 2020] Dumas, P.-Y., Bertoli, S., Bérard, E., Leguay, T., Tavitian, S., Galtier, J., Alric, C., Bidet, A., Delabesse, E., Rieu, J. B., et al. (2020). Delivering hdac over 3 or 5 days as consolidation in aml impacts health care resource consumption but not outcome. *Blood Advances*, 4(16):3840–3849.

[Friberg et al., 2002] Friberg, L. E., Henningsson, A., Maas, H., Nguyen, L., and Karlsson, M. O. (2002). Model of chemotherapy-induced myelosuppression with parameter consistency across drugs. *Journal of Clinical Oncology*, 20:4713–4721.

[Jost et al., 2019] Jost, F., Schalk, E., Rinke, K., Fischer, T., and Sager, S. (2019). Mathematical models for cytarabine-derived myelosuppression in acute myeloid leukaemia. *PLoS One*, 14(7):e0204540.

[Jost et al., 2020] Jost, F., Schalk, E., Weber, D., Döhner, H., Fischer, T., and Sager, S. (2020). Model-based optimal aml consolidation treatment. *IEEE Transactions on Biomedical Engineering*, 67(12):3296–3306.

[Michor and Beal, 2015] Michor, F. and Beal, K. (2015). Improving cancer treatment via mathematical modeling: surmounting the challenges is worth the effort. *Cell*, 163(5):1059–1063. Publisher: Elsevier.

[Ortiz et al., 1993] Ortiz, J. S., Llamazares, A., and López-Vega, J. (1993). Paradoxical myelosuppression with granulocyte-stimulating factor (g-csf) and simultaneous administration chemotherapy. *Medicina clinica*, 101(7):276.

[Stiehl et al., 2018] Stiehl, T., Ho, A. D., and Marciniak-Czochra, A. (2018). Mathematical modeling of the impact of cytokine response of acute myeloid leukemia cells on patient prognosis. *Scientific Reports*, 8(1):2809. Publisher: Nature Publishing Group.

[Stiehl and Marciniak-Czochra, 2012] Stiehl, T. and Marciniak-Czochra, A. (2012). Mathematical modeling of leukemogenesis and cancer stem cell dynamics. *Mathematical Modelling of Natural Phenomena*, 7(1):166–202. Publisher: EDP Sciences.
